# Design, synthesis, and biological screening of a series of 4′-fluoro-benzotriazole-acrylonitrile derivatives as microtubule-destabilising agents (MDAs)

**DOI:** 10.1080/14756366.2022.2111680

**Published:** 2022-08-17

**Authors:** Federico Riu, Roberta Ibba, Stefano Zoroddu, Simona Sestito, Michele Lai, Sandra Piras, Luca Sanna, Valentina Bordoni, Luigi Bagella, Antonio Carta

**Affiliations:** a Department of Medicine, Surgery and Pharmacy, University of Sassari, Sassari, Italy; b Department of Biomedical Sciences, University of Sassari, Sassari, Italy; c Department of Chemical, Physical, Mathematical and Natural Sciences, University of Sassari, Sassari, Italy; d Department of Translational Medicine and New Technologies in Medicine and Surgery, Retrovirus Centre, University of Pisa, Pisa, Italy; e CISUP – Centre for Instrumentation Sharing – University of Pisa, Pisa, Italy; f Center for Biotechnology, College of Science and Technology, Sbarro Institute for Cancer Research and Molecular Medicine, Temple University, Philadelphia, PA, USA

**Keywords:** Benzotriazole-acrylonitrile, antiproliferative compounds, colchicine-binding site inhibitors, cell growth inhibition, molecular docking

## Abstract

**Introduction:** Colchicine-binding site inhibitors are some of the most interesting ligands belonging to the wider family of microtubule-destabilising agents.

**Results:** A novel series of 4′-fluoro-substituted ligands (**5–13**) was synthesised. The antiproliferative activity assays resulted in nM values for the new benzotriazole-acrylonitrile derivatives. Compound **5**, the hit compound, showed an evident blockade of HeLa cell cycle in the G2-M phase, but also a pro-apoptotic potential, and an increase of early and late apoptotic cells in HeLa and MCF-7 cell cycle analysis. Confocal microscopy analysis showed a segmented shape and a collapse of the cytoskeleton, as well as a consistent cell shrinkage after administration of **5** at 100 nM. Derivative **5** was also proved to compete with colchicine at colchicine-binding site, lowering its activity against tubulin polymerisation. In addition, co-administration of **5** and doxorubicin in drug-resistant A375 melanoma cell line highlighted a synergic potential in terms of inhibition of cell viability.

**Discussion:** The 4′-fluoro substitution of benzotriazole-acrylonitrile scaffold brought us a step forward in the optimisation process to obtain compound **5** as promising MDA antiproliferative agent at nanomolar concentration.

## Graphical Abstract

**Figure:**
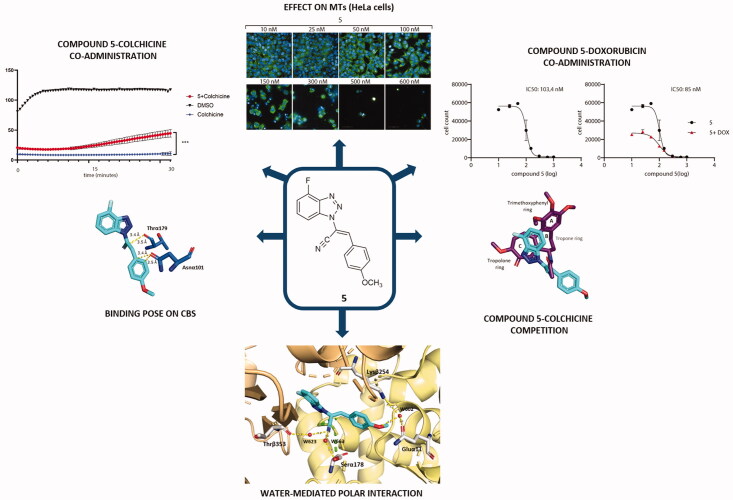

## Introduction

**Figure 1.**
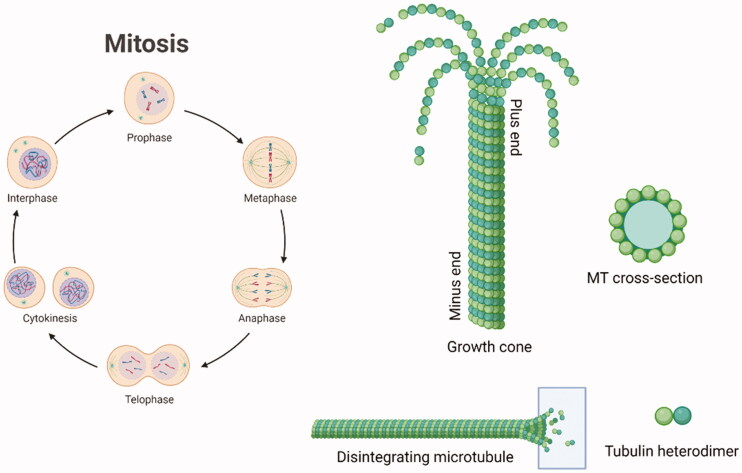
Schematic representation of the mitotic cycle. On the right, a picture of an MT growth cone, made of α,β-tubulin heterodimers. Created (and partly adapted from a template) with BioRender.com.

Cancer is one of the main clinical issues worldwide. Anticancer therapy is still a focus of academic and industrial research, which aims to improve the potency and safety of validated anticancer protocols and to create new ones.^1^ Antitumor compounds targeting the microtubule (MT) structure affect the cell skeleton and the replication process but also act on apoptosis, therefore some of them have been approved for clinical cancer treatment.^2^ Microtubules (MTs) have a fine-tuned dynamic mechanism of polymerisation and depolymerisation dealing with cell division. A single microtubule is constituted by heterodimers of α- and β-tubulin.^3–5^ Tubulin polymerisation is crucial for the creation of microtubules. It is regulated by the hydrolysis of GTP (guanosine-5′-triphosphate) in the β-portion of tubulin dimer. GTP caps stabilise the formed microtubule ends.^6^ The α- and β-hetero-polypeptides of tubulin have about 36–42% similarity to each other and each subunit consists of about 445 amino acids. The 3D structure of the α,β-tubulin heterodimer has been determined by X-ray diffraction (Protein Data Bank Identity [PDB ID]: 4O2B)^7^ and both monomers were shown to surround a GTP molecule. MT growth occurs at the plus end and the shortening at the minus end.^8^^,^^9^ Figure 1 reports a simplified representation of the mitotic cycle and the microtubule depolymerisation at the plus end.

**Figure 2.**
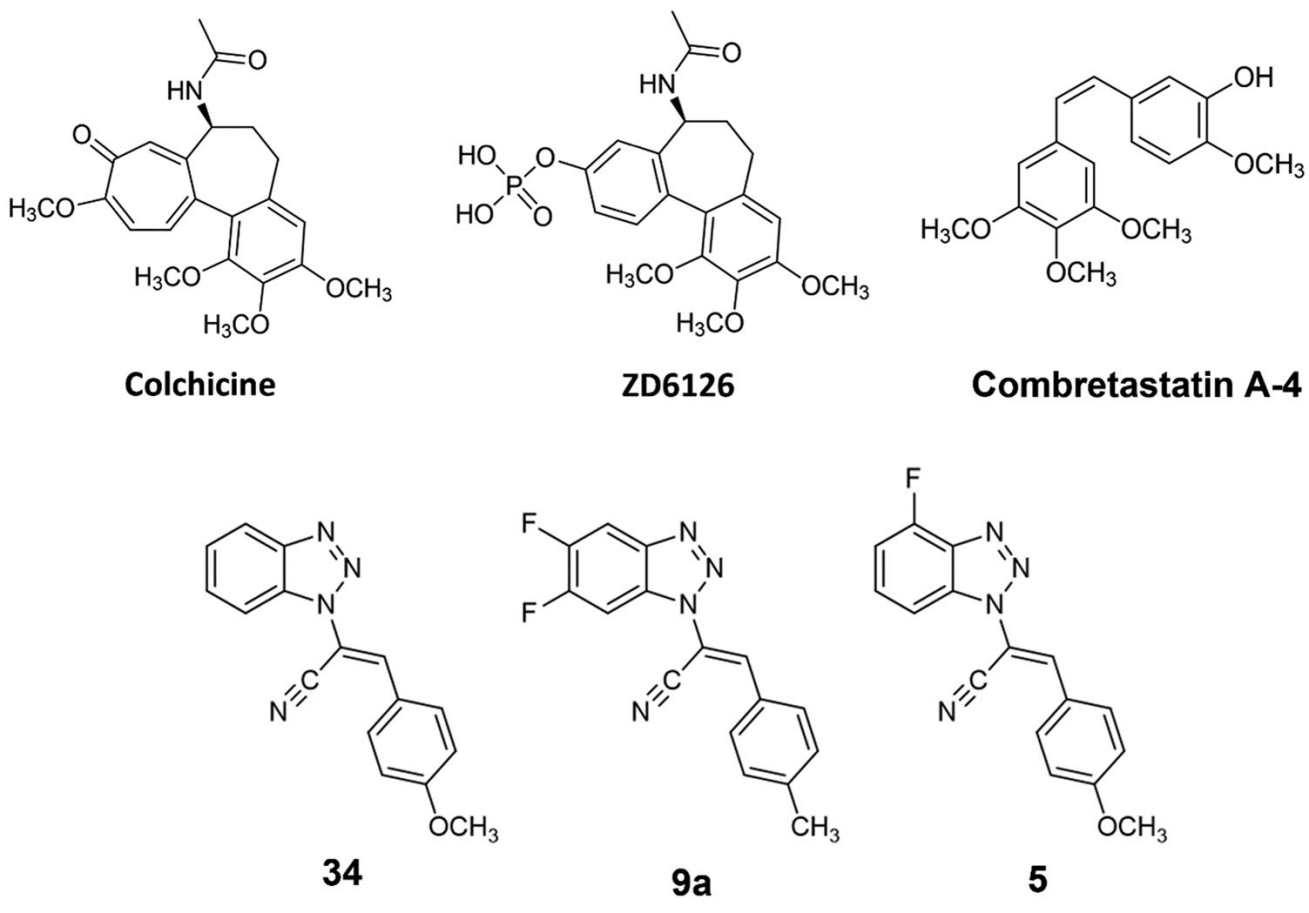
Chemical structures of colchicine and its derivative ZD6126, combretastatin A-4, our previous lead compounds **34** and **9a**, and target compound **5** of the present work.

MT-targeting drugs are commonly divided into microtubule-destabilising agents (MDAs), e.g. colchicine or vinblastine, and microtubule-stabilising agents (MSAs), such as paclitaxel or epothilones. MDAs prevent tubulin polymerisation and the consequent MT assembly; differently, MSAs promote tubulin polymerisation.^10^ Focussing on MDAs therapy, their clinical challenges include low therapeutic windows and innate or acquired drug resistance. Some tumours thought to have microtubule-independent trafficking of key oncogenic proteins seem not to respond to MDAs at all, such as renal cell carcinomas.^11^ One of the most famous MDAs is colchicine (Figure 2), an alkaloid mainly indicated as a treatment of inflammatory diseases, such as recurrent pericarditis treatment,^12^ gout and familial Mediterranean fever.^13^^,^^14^ Colchicine interacts with tubulin heterodimer in the so-called colchicine-binding site (CBS), located at the interface between α- and β-tubulin.^15^ Colchicine-tubulin interaction makes the microtubule polymerisation process energetically unfavourable and prevents the microtubule growth by sterically blocking further addition of tubulin dimers at the plus end.^16^ Colchicine was also shown to alter the mitochondrial membrane potential and release pro-apoptotic factors like caspases, leading to apoptotic cell death.^17^ Despite its multimodal activity, its therapeutic potential against cancer is restrained due to its low therapeutic index. Also, compound ZD6126, a water-soluble phosphate prodrug, initially selected as a gold alternative to colchicine (Figure 2), did not succeeded further as an anticancer agent due to cardiotoxicity and other severe side effects.^18^ Although colchicine failure, multiple efforts were made to develop clinically potent colchicine-binding site inhibitors (CBSIs). CBSIs are some of the most interesting MDAs as they target the β-subunit of tubulin in its curved and unassembled form and prevent it from adopting a microtubular straight structure.^19^^,^^20^ So far, combretastatin A-4 is arguably the most successful representative of the CBSI family.^21^^,^^22^

Many other CBISs have been synthesised in the last decades, including compound **34**,^23^ an acrylonitrile derivative developed by us and identified as CBSI through computational and experimental analysis.^23–29^ Following this direction, we decided to explore the effect of a difluoro substitution on the benzotriazole scaffold, therefore a series of 5′,6′-difluoro-benzotriazole-acrylonitrile derivatives were designed, synthesised and screened, indicating the hit compound **9a** as the most potent.^30^ The two hit compounds **34** and **9a** showed a consistent inhibition of cancer cell growth and a general blockage of the cancer cell cycle in the G2/M phase. Starting from this background, in the present work, we carried out a hit optimisation strategy on the heterocycle scaffold consisting of the mono-fluoro substitution in position C–4′ of the benzotriazole in place of the 5′6′-difluoro functionalisation previously reported, in order to reduce the molecular weight while maintaining the halogen interactions that were proved successful in the former series.

## Materials and methods

### Molecular docking

The academic program AutoDock Vina 1.1.2. was employed for the molecular docking investigation.^31^ The tubulin-colchicine crystal structure (PDB ID: 4O2B; 2.30 Å of resolution) was downloaded from the Protein Data Bank.^32^ The starting crystal structure of tubulin was processed: water molecules were removed, as well as GOL (glycerol), the buffer MES (2-(*N*-morpholino)-ethanesulfonic acid), Ca^2+^ and Mg^2+^ ions, GDP (guanosine-5′-diphosphate) and GTP. For a less time-consuming process, only the A and B chains were considered for the simulations. Protein Data Bank, Partial Charge, Atom Type (PDBQT) files of protein and ligands were obtained through PyMOL 2.3.4.^33^ and AutoDockTools (ADT).^34^ ADT Grid Box was set as 32–32-32 Å (x,y,z). Docking was performed by setting amino acids Serα178, Thrα179, Alaα180, Alaα181 flexible, considered crucial for the binding affinity of colchicine in its binding site on tubulin.^7^ The exhaustiveness was set to 64 and 10 best-predicted poses were generated for each calculation. The range of binding energy was set as ≤ 2 kcal/mol, above that of the top-ranked pose. LigPlot + v.2.2.^35^ generated 2D representations of protein-ligand interactions with a 5 Å cut-off from the ligand centre of mass. 3D figures were taken through PyMOL, with a 3000 dpi of rendering. A PC with an Intel® Core™ i7–9705H CPU @ 2.60 GHz with 8 GB RAM (operating system: Ubuntu 16.04) was employed, utilising 6 CPUs for each simulation.

### Chemistry

#### Synthesis and compounds’ characterisation

**Scheme 1.**
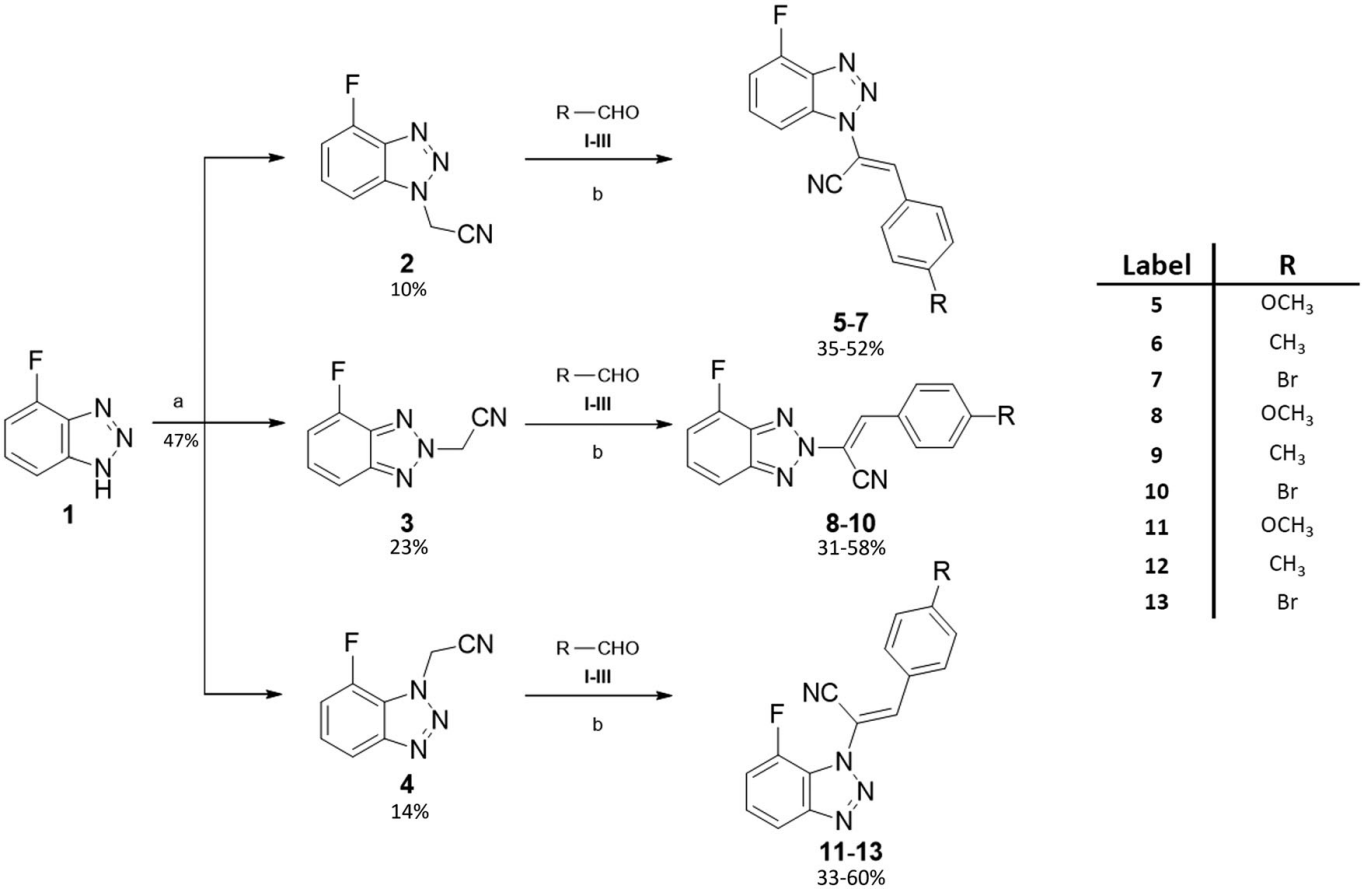
General procedure for final compounds (**5**–**13**). ^a^Reaction conditions: chloroacetonitrile/KOH (1:1.1), CH_3_CN, reflux, o/n. ^b^Reaction conditions: (A) DMA (ratio 1:1.2), CH_3_CN; (B) DIMCARB (ratio 1:1.2), CH_3_CN; (C) Cs_2_CO_3_ (ratio 1:1.2), toluene; yields:31–60%.

As reported in Scheme 1, compound **1** was alkylated to yield the acetonitrile isomers **2–4**. Final compounds **5–7**, **8–10,** and **11–13** were obtained *via* Knoevenagel condensation of intermediates **2–4** with aldehydes **I–IV**. Regarding the spectroscopic characterisation, the nuclear magnetic resonance (NMR) spectra were registered in a solution of deuterated dimethyl sulfoxide (DMSO-d_6_) and recorded with a Bruker Avance III 400 NanoBay (400 MHz). ^1^H NMR *δ_s_* (chemical shifts) are reported in parts per million (ppm) downfield from tetramethylsilane (TMS), as the internal standard. Chemical shift values are reported in ppm and coupling constants (*J*) are reported in Hertz (Hz). In some cases, D_2_O was added to assign exchangeable protons (OH, NH). Signal splitting is represented as s (singlet), d (doublet), dd (doublet of doublets), ddd (doublet of doublets of doublets), t (triplet), dt (doublet of triplets), m (multiplet), and wm (wide multiplet). ^13 ^C NMR chemical shifts are reported downfield from TMS, jmod (*J*-modulated spin-echo for X-nuclei coupled to H–1, to determine the number of attached protons) was employed. The assignment of resonances was evaluated based on relative intensity, chemical shift, and fine structure using assignment predictions by www.nmrdb.org. ESI–MS characterisation involved the preparation of each compound, dissolved in CH_3_CN for HPLC (1.0–2.0 ppm). Full mass spectra were recorded on a Q Exactive Plus Hybrid Quadrupole-Orbitrap mass spectrometer (Thermo Fisher Scientific), in positive- and negative-ion mode. The solutions were infused into the ESI chamber at a flow rate of 5.00 μl/min. The spectra were recorded in the *m/z* range 150–800 at a resolution of 140,000 and accumulated for approximately 2 min to increase the signal-to-noise ratio. Positive-ion acquisitions were conducted with the following parameters: spray voltage 2300 V, capillary temperature 250 °C, sheath gas 10 (arbitrary units), auxiliary gas 3 (arbitrary units), sweep gas 0 (arbitrary units) and probe heater temperature 50 °C. Negative-ion measurements were done as follows: spray voltage −1900 V, capillary temperature 250 °C, sheath gas 20 (arbitrary units), auxiliary gas 5 (arbitrary units), sweep gas 0 (arbitrary units), probe heater temperature 50 °C. ESI–MS spectra analysis was made by using Thermo Xcalibur 3.0.63 software (Thermo Fisher Scientific), and the Xtract tool was used to extract the deconvoluted monoisotopic masses, as an averaged value. Commercially available chemicals were purchased from Carlo Erba Reagents and Sigma Aldrich. Evaporation was performed *in vacuo* (rotating evaporator). Sodium sulphate was always used as the drying agent. Celite® 545 was used as filter agent. A mixture of PS (petroleum spirit) and EA (ethyl acetate), ratio 8:2, was employed to calculate the retention factors (R*_f_*), using Thin Layer Chromatographies (TLCs) on Merck F-254 plates. Melting points (m.p.) were measured in a Köfler hot stage in open capillaries or Digital Electrothermal melting point apparatus and were uncorrected.

### Biology

#### NCI60 in vitro screening

A first NCI60 screening was performed to assess the *in vitro* anticancer activity through the Developmental Therapeutics Program of the National Cancer Institute (NCI, Bethesda, USA). All detailed NCI reports are depicted in Table S1 and Figures S29-44, and the experimental methods are explained on the NCI website (https://dtp.cancer.gov). To summarise, a first single compound dose of 10 µM was administered to the entire panel of NCI60 cancer cell lines. If the growth percentage, 50% of growth inhibition, tumour growth inhibition and 50% of lethal concentration profile of the molecule agrees with the selection criteria, the above-mentioned derivative is administered to the entire NCI60 panel of cancer cell lines in 5 × 10-fold dilutions, from 100 µM up to 0.01 µM.

#### Cell culture

HeLa, MCF-7, and A375 cell lines were cultured in Dulbecco’s Modified Eagle’s Medium (Gibco). The medium was supplemented with 10% foetal bovine serum, 100 units/ml penicillin, 100 µg/ml streptomycin (Gibco), and 1% l-glutamine. Cells were incubated at 37 °C with 5% CO_2_ in humidified air. Every cell line was tested for mycoplasma contamination as previously described.^36^ Doxorubicin (Sigma Aldrich) were resuspended in DMSO and administered for 24 h at 2 μM.

#### Proliferation assay (XTT)

Cells were seeded at a density of 1000 cells/well in a plate-based 96-well on different sizes, population doublings, and phenotypes. Twenty-four hours after seeding, cells were treated for 48 h with different concentrations of all compounds in Scheme 1 in a range from 0.125 μM to 2 μM in a final volume of 100 μl. DMSO and compound **34** were used as negative and positive controls, respectively. After 48 h, the XTT assay was performed using the Cell Proliferation Kit II (Roche, Basel, Switzerland). 0.5 μl of XTT electron-coupling reagent and 25 μl of labelling reagent were resuspended in 74.5 μl of medium (final volume 100 μl/well) and incubated for 4 h at 37 °C. After incubation, absorbance was measured at 490 nm with a spectrophotometric plate reader (SPECTRAMax 384 PLUS) and the results obtained were used to calculate IC_50_ values. Based on the IC_50_ value, compounds **5** and **12** were selected for subsequent experiments. All experiments were performed in triplicate.

#### Cell cycle analysis

Cells were seeded at 50% confluence on 6-cm dishes. After 24 h, cells were treated with 2 µM of compounds **5** and **12** for 24 h. Afterwards, floating (necrotic and in late apoptosis cells) and adherent cells were first centrifuged at 3000 rpm for 5 min; washed in PBS and then fixed with ice-cold 70% ethanol and incubated at −20 °C overnight. Fixed cells were washed twice with cold PBS and then resuspended with 150 μl of a solution of PBS and 10 μl/test of 7-aminoactinomycin D (7-AAD; Bioscience, San Diego, CA) and incubated for 20 min at room temperature. Cell cycle assay was performed by flow cytometry using BD FACS CANTO II and collecting approximately 20,000 events for each sample. Data were analysed by BD FACS DIVA software. All experiments were performed in triplicate.

#### Annexin V assay

Annexin V assay was performed using the FITC Annexin V Apoptosis Detection Kit II (BD Pharmingen, San Jose, CA), according to the manufacturer’s protocol. After 24 h, cells were treated at concentrations of 2 µM of compounds **5** and **12**. After 24 h, floating and adherent cells were harvested, centrifuged, and washed twice with cold PBS. Annexin V 1x buffer was used to resuspend the cells. Five microliters of 7-AAD and 5 μl of V-fluorofluorine-annexin isothiocyanate were added to the cells and incubated in the dark for 15 min at room temperature. Subsequently, 200 μl of Annexin V 1× buffer was added in each sample. Flow cytometry was performed immediately using BD FACS CANTO II. 20,000 events were analysed using BD FACS DIVA software. All experiment were performed in triplicate.

#### Protein extraction and Western blot

Total proteins were extracted using lysis buffer (20 mM Tris HCl pH 8; 137 mM NaCl; 10% glycerol; 1% Nonidet P-40; 2 mM EDTA) with the addition of Protease Inhibitor Cocktail (Roche, Basel, Switzerland). Twenty micrograms of protein was dissolved in 8% SDS/PAA (Sodium Dodecyl Sulphate - PolyAcrylamide) and then transferred to a nitrocellulose membrane (GE, Healthcare, Whatman) for 1 h at 4 °C and 100 V. The nitrocellulose membrane was blocked with 5% bovine serum albumin for 1 h at room temperature. Blots were incubated overnight at 4 °C with primary anti-PARP (Cell Signalling, Boston, MA) and anti-GAPDH (Santa Cruz Biotechnology, CA) antibodies. Membranes were incubated with antibodies-conjugated secondary peroxidase and the signal was detected with Western Lightning Plus-ECL (PerkinElmer, Waltham, MA, USA).

#### Colchicine-5 competition assay

Tubulin polymerisation assay was carried out using the kit supplied by Cytoskeleton (BK006P, Cytoskeleton, Denver, CO). The assay is based on the principle that light is scattered by microtubules to an extent that fluorescence is proportional to the concentration of the microtubule polymer. The absorbance was recorded at 340 nm at 37 °C for 30 min with fixed acquisitions every 2 s. Colchicine-**5** competition assay was performed incubating equimolar (1 μM) concentrations of colchicine and compound **5** with tubulin. After 30 min, fluorescence emitted by tubulin polymerisation was recorded by reading absorbance for 30 min.

#### High-content imaging of HeLa cells and IC_50_ calculation

Imaging experiments were performed using an Operetta CLS high-content imaging device (PerkinElmer, Hamburg, Germany), and analysed with Harmony 4.6 software (PerkinElmer). To assess the tubulin integrity, we acquired HeLa cells images using 63× magnification, taking 25 fields per sample in biological and technical triplicates. Data were analysed using the following building blocks: 1—Find Nuclei, 2—Find Cytoplasm (Tubulin+). IC_50_ was obtained by counting the number of nuclei in each field. Cell dimensions were assessed using the following building blocks: 1—Find Nuclei, 2—Find Cytoplasm (Tubulin+), 3—Calculate Morphology properties (Area), as previously described.^37–39^ IC_50_s were calculated using the number of cells detected in every well as indexing parameter by Harmony 4.6 software as described here.^40^

## Experimental section

### Materials

All starting materials and the different aldehydes (**I–IV**) were commercially purchased. Compound **1** was synthesised as previously reported.^41^

#### General procedure for the synthesis of intermediates 2-(4-fluoro-1H-benzo[d][1,2,3]triazol-1-yl)acetonitrile (2), 2-(4-fluoro-2H-benzo [d][1,2,3]triazol-2-yl)acetonitrile (3) and 2-(7-fluoro-1H-benzo[d] [1,2,3]triazol-1-yl)acetonitrile (4)

Methods: Compound **1/**chloroacetonitrile/KOH (1:1:1.1), CH_3_CN, reflux, overnight. The three isomers were separated by flash chromatography (PS/EA 9/1). C_8_H_5_FN_4_; MW: 176.15. Total yield of the three isomers was 47%. Compound **2:** Yellow solid; 10% yield (0.18 g, 1.0 mmol); m.p. 73.4–75.6 °C. R*_f_* 0.19. ^1^H NMR (DMSO-d_6_): *δ* 7.82 (1H, d, ^1^*J*_H–H_= 8.4 Hz, H–7), 7.69 (1H, dt, H–6), 7.34 (1H, dd, ^1 ^*J* = 10.8 Hz, ^2 ^*J* = 8.0 Hz, H–5), 6.22 (2H, s, CH_2_). ^13 ^C NMR (DMSO-d_6_): *δ* 152.15 (C, d, ^1^*J*_C–F_= 254.0 Hz, C–F), 135.26 (C, d, ^1^*J*_C–F_= 7.0 Hz, C), 135.03 (C, d, ^1^*J*_C–F_ = 19.0 Hz, C), 129.74 (C, d, ^1^*J*_C–F_= 7.0 Hz, CH), 114.67 (C≡N), 109.41 (C, d, ^1^*J*_C–F_= 16.0 Hz, CH), 106.87 (C, d, ^1^*J*_C–F_= 5.0 Hz, CH), 35.95 (CH_2_). ESI–MS *m/z* calcd for C_8_H_5_FN_4_ 177.0571, found 176.8251 [M + H]^+^. Compound **3:** Yellow solid; 23% yield (0.45 g, 2.6 mmol); m.p. 85.2–87.1 °C; R*_f_* 0.58. ^1^H NMR (DMSO-d_6_): *δ* 7.92 (1H, d, ^1^*J*_H–H_= 8.4 Hz, H–7), 7.58 (1H, dt, H–6), 7.42 (1H, dd, ^1^*J*_H–H_= 5.0 Hz, ^2^*J*_H–H_= 2.0 Hz, H–5), 6.40 (2H, s, CH_2_). ^13 ^C NMR (DMSO-d_6_): *δ* 151.24 (C, d, ^1 ^*J* = 255.0 Hz, C–F), 146.69 (C, d, ^1^*J_C–F_*= 3.0 Hz, C), 135.17 (C, d, ^1^*J_C–F_*= 16.0 Hz, C), 127.91 (C, d, ^1^*J_C–F_*= 7.0 Hz, CH), 114.56 (C, d, ^1^*J_C–F_*= 5.0 Hz, CH), 114.09 (C≡N) 110.83 (C, d, ^1^*J_C–F_*= 16.0 Hz, CH), 44.18 (CH_2_). ESI–MS *m/z* calcd for C_8_H_5_FN_4_ 177.0571, found 177.0913 [M + H]^+^. Compound **4:** White solid; 14% yield (0.27 g, 1.5 mmol); m.p. 89.1–91.2 °C; R*_f_* 0.38. ^1^H NMR (DMSO-d_6_): *δ* 7.89 (1H, d, ^1 ^*J* = 8.4 Hz, H–7), 7.56 (1H, dt, H–6), 7.39 (1H, dd, ^1 ^*J* = 4.0 Hz, ^2 ^*J* = 1.0 Hz, H–5), 6.38 (2H, s, CH_2_). ^13 ^C NMR (DMSO-d_6_): *δ* 148.54 (C), 146.99 (C, d, C–F), 127.91 (C, d, ^1^*J_C–F_*= 6.0 Hz, CH), 122.26 (C, d, ^1^*J_C–F_*= 14.0 Hz, C), 115.98 (C, d, ^1^*J_C–F_*= 5.0 Hz, CH), 110.83 (CH–CF), 114.83 (C≡N), 113.26 (C, d, ^1^*J_C–F_*= 16.0 Hz, CH), 37.44 (CH_2_). ESI–MS *m/z* calcd for C_8_H_5_FN_4_ 177.0571, 178.0604, found 176.8253, 178.2626 [M + H]^+^.

#### General procedure for compounds (E)-2-(4-fluoro-1H-benzo[d] [1,2,3]triazol-1-yl)-3-(R)acrylonitrile (5–7), (E)-2-(4-fluoro-2H-benzo[d][1,2,3]triazol-2-yl)-3-(R)acrylonitrile (8–10), (E)-2-(7-fluoro-1H-benzo[d] [1,2,3]triazol-1-yl)-3-(R)acrylonitrile (11–13)

Compounds **5–7** were obtained via Knoevenagel condensation of **2** and the appropriate aldehydes **I–IV**. **8–10** were synthesised by reaction of **3** and **I–IV**, while intermediate **4** and the aldehydes **I–IV** led to compounds **11–13**. Reaction conditions: (A) DMA (ratio 1:1.2), CH_3_CN for **9–10** and **12**; (B) DIMCARB (ratio 1:1.2), CH_3_CN for **5**, **6**, **7**; (C) Cs_2_CO_3_ (ratio 1:1.2), toluene for **8**, **11**, **13**. Work up: (A) filtration; (B) crystallisation from EtOH; (C) flash chromatography (PS/EA 9/1).

#### (E)-2-(4-fluoro-1H-benzo[d][1,2,3]triazol-1-yl)-3-(4-methoxyphenyl) acrylonitrile (5)

Work-up procedure (B). Yellow solid; C_16_H_11_FN_4_O, MW: 294.28; 52% yield (0.23 g); m.p. 141.2–143.4 °C; R*_f_* 0.35. ^1^H NMR (400 MHz, DMSO-d_6_): *δ* 8.23 (1H, s, =CH), 8.03 (2H, d, ^1^*J*_H–H_= 8.8 Hz, H–3″,5″), 7.86 (1H, d, ^1^*J*_H–H_= 8.4 Hz, H–7′), 7.75 (1H, dt, H–6′), 7.43 (1H, dd, ^1 ^*J* = 10.4 Hz, ^2 ^*J* = 8.0 Hz, H5′), 7.21 (2H, d, ^1^*J*_H–H_= 8.4 Hz, H–2″,6″), 3.89 (3H, s, OCH_3_). ^13 ^C NMR (DMSO-d_6_): *δ* 162.48 (C), 152.27 (C, d, ^1^*J_C–F_*= 255.0 Hz, C–F), 143.01 (=CH), 135.17 (C, d, ^1^*J_C–F_*= 19.0 Hz, C), 134.57 (C, d, ^1^*J_C–F_*= 6.0 Hz, C), 131.96 (2CH), 132.43 (2CH), 130.95 (C, d, ^1^*J_C–F_*= 8.0 Hz, CH), 123.17 (C), 115.31 (2CH), 115.00 (C≡N), 105.42 (C, d, ^1^*J_C–F_* = 16.0 Hz, CH), 108.08 (C, d, ^1^*J_C–F_* = 5.0 Hz, CH), 102.99 (C), 56.12 (OCH_3_). ESI–MS *m/z* calcd for C_16_H_11_FN_4_O 295.0989, 296.1023, found 295.0989, 296.1023 [M + H]^+^.

#### (E)-2-(4-fluoro-1H-benzo[d][1,2,3]triazol-1-yl)-3-(p-tolyl)acrylonitrile (6)

Work-up procedure (C, PS/EA 7/3). Yellow solid; C_16_H_11_FN_4_, MW: 278.10; 50% yield (0.36 g); m.p. 128.1–130.3 °C; R*_f_* 0.27. ^1^H NMR (DMSO-d_6_): *δ* 8.27 (1H, s, =CH), 7.93 (2H, d, ^1^*J*_H–H_= 8.0 Hz, H–3″,5″), 7.89 (1H, d, ^1^*J*_H–H_= 8.0 Hz, H–7′), 7.75 (1H, dt, H–6′), 7.45 (2H, d, ^1^*J*_H–H_= 8.0 Hz, H–2″,6″), 7.44 (1H, m, H5′), 2.43 (3H, s, CH_3_). ^13 ^C NMR (DMSO-d_6_): *δ* 157.22 (C), 152.26 (C, d, ^1^*J_C–F_*= 255.0 Hz, C–F), 142.73 (=CH), 135.23 (C, d, ^1^*J_C–F_*= 19.0 Hz, C), 134.44 (C, d, ^1^*J_C–F_*= 6.0 Hz, C), 130.58 (C, d, ^1^*J_C–F_*= 7.0 Hz, CH), 129.90 (2CH), 129.74 (2CH), 127.65 (C), 114.11 (C≡N), 110.04 (C, d, ^1^*J_C–F_* = 16.0 Hz, CH), 107.71 (C, d, ^1^*J_C–F_*= 5.0 Hz, CH), 104.67 (C), 21.22 (OCH_3_). ESI–MS *m/z* calcd for C_16_H_11_FN_4_ 279.1040, 280.1074, found 279.1032, 280.1187 [M + H]^+^.

#### (E)-3-(4-bromophenyl)-2-(4-fluoro-1H-benzo[d][1,2,3]triazol-1-yl)acrylonitrile (7)

Work-up procedure (B). Yellow solid; C_15_H_8_BrFN_4_, MW: 343.15; 35% yield (0.20 g); m.p. 158.6–160.3 °C; R*_f_* 0.55. ^1^H NMR (DMSO-d_6_): *δ* 8.31 (1H, s, =CH), 7.94 (3H, m, H–3″,5″,7′), 7.86 (2H, d, ^1^*J*_H–H_= 8.8 Hz, H–2″,6″), 7.77 (1H, dt, H–6′), 7.45 (1H, dd, *^1^J_H–F_*= 10.6 Hz, *^2^J_H–F_*= 2.0 Hz, H–5′). ^13 ^C NMR (DMSO-d_6_): *δ* 152.25 (C, d, ^1^*J_C–F_*= 255.0 Hz, C–F), 140.53 (CH), 135.29 (C, d, ^1^*J_C–F_* = 19.0 Hz, C), 134.24 (C, d, ^1^*J_C–F_*= 6.0 Hz, C), 132.33 (2CH), 131.47 (2CH), 130.70 (C, d, ^1^*J_C–F_*= 7.0 Hz, CH), 129.76 (C), 125.56 (C), 113.66 (C≡N), 110.18 (C, d, ^1^*J_C–F_*= 16.0 Hz, CH), 107.89 (C, d, ^1^*J_C–F_*= 4.0 Hz, CH), 106.67 (C). ESI–MS *m/z* calcd for C_15_H_8_BrFN_4_ 342.9989, found 342.9972 [M + H]^+^.

#### (E)-2-(4-fluoro-2H-benzo[d][1,2,3]triazol-2-yl)-3-(4-methoxyphenyl)acrylonitrile (8)

Work-up procedure (C). Yellow powder; C_16_H_11_FN_4_O, MW: 294.28; 42% yield (0.23 g); m.p. 127.2–129.2 °C; R*_f_* 0.69. ^1^H NMR (DMSO-d_6_): *δ* 8.72 (1H, s, =CH), 8.10 (2H, d, ^1^*J*_H–H_= 8.8 Hz, H–2″,6″), 7.89 (1H, d, ^1^*J*_H–H_= 8.4 Hz, H–7′), 7.55 (1H, dt, H–6′), 7.40 (1H, dd, ^1^*J*_H–H_= 11.0 Hz, ^1^*J*_H–H_= 8.0 Hz, H–5′), 7.20 (2H, d, ^1^*J*_H–H_= 8.8 Hz, H–3″,5″), 3.89 (3H, s, OCH_3_). ^13 ^C NMR (DMSO-d_6_): *δ* 162.63 (C), 151.10 (C, d, ^1^*J_C–F_*= 256.0 Hz, C–F), 146.62 (C, d, ^1^*J_C–F_*= 3.0 Hz, C), 138.84 (CH), 135.29 (C, d, ^1^*J_C–F_*= 16.0 Hz, C), 132.37 (2CH), 128.53 (C, d, ^1^*J_C–F_*= 6.0 Hz, CH), 122.32 (C), 115.00 (2CH), 114.50 (C, d, ^1^*J_C–F_*= 5.0 Hz, CH), 113.48 (C≡N), 111.47 (C, d, ^1^*J_C–F_*= 15.0 Hz, CH), 108.70 (C), 55.66 (OCH_3_). ESI–MS *m/z* calcd for C_16_H_11_FN_4_O 295.0989, found 295.1803 [M + H]^+^.

#### (E)-2-(4-fluoro-2H-benzo[d][1,2,3]triazol-2-yl)-3-(p-tolyl)acrylonitrile (9)

Work-up procedure (A). Yellow solid; C_15_H_8_F_2_N_4_. MW: 282.07; 58% yield (0.28 g); m.p. 135.6–137.2 °C; R*_f_* 0.70. ^1^H NMR (DMSO-d_6_): *δ* 8.75 (1H, s, =CH), 7.99 (2H, d, ^1^*J*_H–H_= 8.4 Hz, H–3″,5″), 7.90 (1H, d, ^1^*J*_H–H_= 8.8 Hz, H–7′), 7.57 (1H, dt, H–6′), 7.44 (2H, d, ^1^*J*_H–H_= 7.6 Hz, H–2″,6″), 7.41 (1H, m, H–5′), 2.42 (3H, s, CH_3_). ^13 ^C NMR (DMSO-d_6_): *δ* 151.12 (C, d, ^1^*J_C–F_*= 256.0 Hz, C–F), 146.66 (C, d, ^1^*J_C–F_*= 4.0 Hz, C), 143.01 (C), 139.02 (CH), 135.40 (C, d, ^1^*J_C–F_*= 16.0 Hz, C), 130.10 (2CH), 129.97 (2CH), 128.71 (C, d, ^1^*J_C–F_*= 6.0 Hz, CH), 127.24 (C), 114.62 (C, d, ^1^*J_C–F_*= 5.0 Hz, CH), 113.09 (C≡N), 111.61 (C, d, ^1^*J_C–F_*= 16.0 Hz, CH), 110.46 (C), 21.24 (CH_3_). ESI–MS *m/z* calcd for C_15_H_8_F_2_N_4_ 283.07898, 284.08233, found 283.9893, 284.1545 [M + H]^+^.

#### (E)-3-(4-bromophenyl)-2-(4-fluoro-2H-benzo[d][1,2,3]triazol-2-yl)acrylonitrile (10)

Work-up procedure (A). Light brown solid; C_15_H_8_BrFN_4_, MW: 343.15; 31% yield (0.18 g); m.p. 137.2–138.1 °C; R*_f_* 0.71. ^1^H NMR (DMSO-d_6_): *δ* 8.79 (1H, s, =CH), 8.01 (2H, d, ^1^*J*_H–H_= 8.4 Hz, H–3″,5″), 7.91 (1H, d, ^1^*J*_H–H_= 8.8 Hz, H–7′), 7.85 (2H, d, ^1^*J*_H–H_= 8.4 Hz, H–2″,6″), 7.57 (1H, dt, H–6′), 7.42 (1H, dd, ^1^*J*_H–H_= 10.8 Hz, ^2^*J*_H–H_= 7.6 Hz, H–5′). ^13 ^C NMR (DMSO-d_6_): *δ* 151.12 (C, d, ^1^*J_C–F_*= 256.0 Hz, C–F), 146.72 (C, d, ^1^*J_C–F_*= 3.0 Hz, C), 137.63 (CH), 135.53 (C, d, ^1^*J_C–F_*= 16.0 Hz, C), 132.39 (2CH), 131.77 (2CH), 129.33 (C), 128.93 (C, d, ^1^*J_C–F_*= 6.0 Hz, CH), 125.89 (C), 114.68 (C, d, ^1^*J_C–F_*= 6.0 Hz, CH), 112.71 (C≡N), 112.0 (C), 111.79 (C, d, ^1^*J_C–F_*= 16.0 Hz, CH). ESI–MS *m/z* calcd for C_15_H_8_BrFN_4_ 344.9968, found 344.9965 [M + H]^+^.

#### (E)-2-(7-fluoro-1H-benzo[d][1,2,3]triazol-1-yl)-3-(4-methoxyphenyl)acrylonitrile (11)

Work-up procedure (C). Yellow powder; C_16_H_11_FN_4_O, MW: 294.28; 60% yield (0.25 g); m.p. 117.4–119.3 °C; R*_f_* 0.35. ^1^H NMR (DMSO-d_6_): *δ* 8.28 (1H, s, =CH), 8.04 (2H, d, ^1^*J*_H–H_= 9.2 Hz, H–3″–5″), 7.64 (2H, m, H–5′,6′), 7.27 (2H, d, ^1^*J*_H–H_= 8.8 Hz, H–2″,6″), 3.95 (OCH_3_). ^13 ^C NMR (DMSO-d_6_): *δ* 162.67 (C), 148.16 (C, d, ^1^*J_C–F_*= 16.0 Hz, C–F), 145.58 (CH), 131.90 (2CH), 126.11 (C, d, ^1^*J_C–F_*= 6.0 Hz, C), 122.39 (C), 122.09 (C, d, ^1^*J_C–F_*= 13.0 Hz, C), 116.26 (C, d, ^1^*J_C–F_*= 4.0 Hz, CH), 115.10 (2CH), 114.88 (C≡N), 114.33 (C, d, ^1^*J_C–F_*= 17.0 Hz, CH), 102.21 (C), 55.67 (OCH_3_). ESI–MS *m/z* calcd for C_16_H_11_FN_4_O 295.0989, found 295.0987 [M + H]^+^.

#### (E)-2-(7-fluoro-1H-benzo[d][1,2,3]triazol-1-yl)-3-(p-tolyl)acrylonitrile (12)

Work-up procedure (A). White powder; C_16_H_11_FN_4_, MW: 278.10; 38% yield (0.18 g); m.p. 145.3–147.9 °C; R*_f_* 0.50. ^1^H NMR (DMSO-d_6_): *δ* 8.26 (1H, s, =CH), 8.09 (1H, d, ^1^*J*_H–H_= 8.0 Hz, H–7′), 7.89 (2H, d, ^1^*J*_H–H_= 8.4 Hz, H–2″,6″), 7.60 (2H, m, H–5′,6′), 7.46 (2H, d, ^1^*J*_H–H_= 8.0 Hz, H–3″,5″), 2.43 (3H, s, CH_3_). ^13 ^C NMR (DMSO-d_6_): *δ* 148.13 (C, d, ^1^*J_C–F_*= 19.0 Hz, C–F), 145.58 (=CH), 143.18 (C), 130.09 (2CH), 129.86 (C, d, ^1^*J_C–F_*= 12.0 Hz, CH), 129.64 (2CH), 27.28 (C), 126.24 (C, d, ^1^*J_C–F_*= 6.0 Hz, CH), 122.09 (C, d, ^1^*J_C–F_*= 13.0 Hz, C), 116.25 (C, d, ^1^*J_C–F_*= 9.0 Hz, CH), 114.43 (C, d, ^1^*J_C–F_*= 22.0 Hz, CH), 114.49 (C≡N), 104.25 (C), 21.22 (OCH_3_). ESI–MS *m/z* calcd for C_16_H_11_FN_4_ 279.1040, found 279.1037 [M + H]^+^.

#### (E)-3-(4-bromophenyl)-2-(7-fluoro-1H-benzo[d][1,2,3]triazol-1-yl)acrylonitrile (13)

Work-up procedure (C). Beige powder; C_15_H_8_BrFN_4_; MW: 343.15; 33% yield: (0.19 g); m.p. 109.1–111.2 °C; R*_f_* 0.45. ^1^H NMR (DMSO-d_6_): *δ* 8.30 (1H, s, =CH), 8.10 (1H, d, ^1^*J*_H–H_= 4.0 Hz, H–7′), 7.91 (2H, d, ^1^*J*_H–H_= 8.8 Hz, H–3″,5″), 7.87 (2H, d, ^1^*J*_H–H_= 7.6 Hz, H–2″,6″), 7.56 (2H, m, H–5′,6′). ^13 ^C NMR (DMSO-d_6_): *δ* 148.15 (C, d, ^1^*J_C–F_*= 25.0 Hz, C–F), 145.53 (C), 143.77 (=CH), 132.57 (2CH), 131.34 (2CH), 129.36 (C), 126.31 (C, d, ^1^*J_C–F_*= 6.0 Hz, CH), 125.98 (C), 121.84 (C, d, ^1^*J_C–F_*= 13.0 Hz, C), 116.34 (C, d, ^1^*J_C–F_*= 5.0 Hz, CH), 114.60 (C, d, ^1^*J_C–F_*= 17.0 Hz, CH), 114.02 (C≡N), 106.08 (C). ESI–MS *m/z* calcd for C_15_H_8_BrFN_4_ 340.9832, 342.9812, found 341.1957, 342.1990 [M – H]^–^.

## Results and discussion

### Molecular modelling and drug design

**Figure 3.**
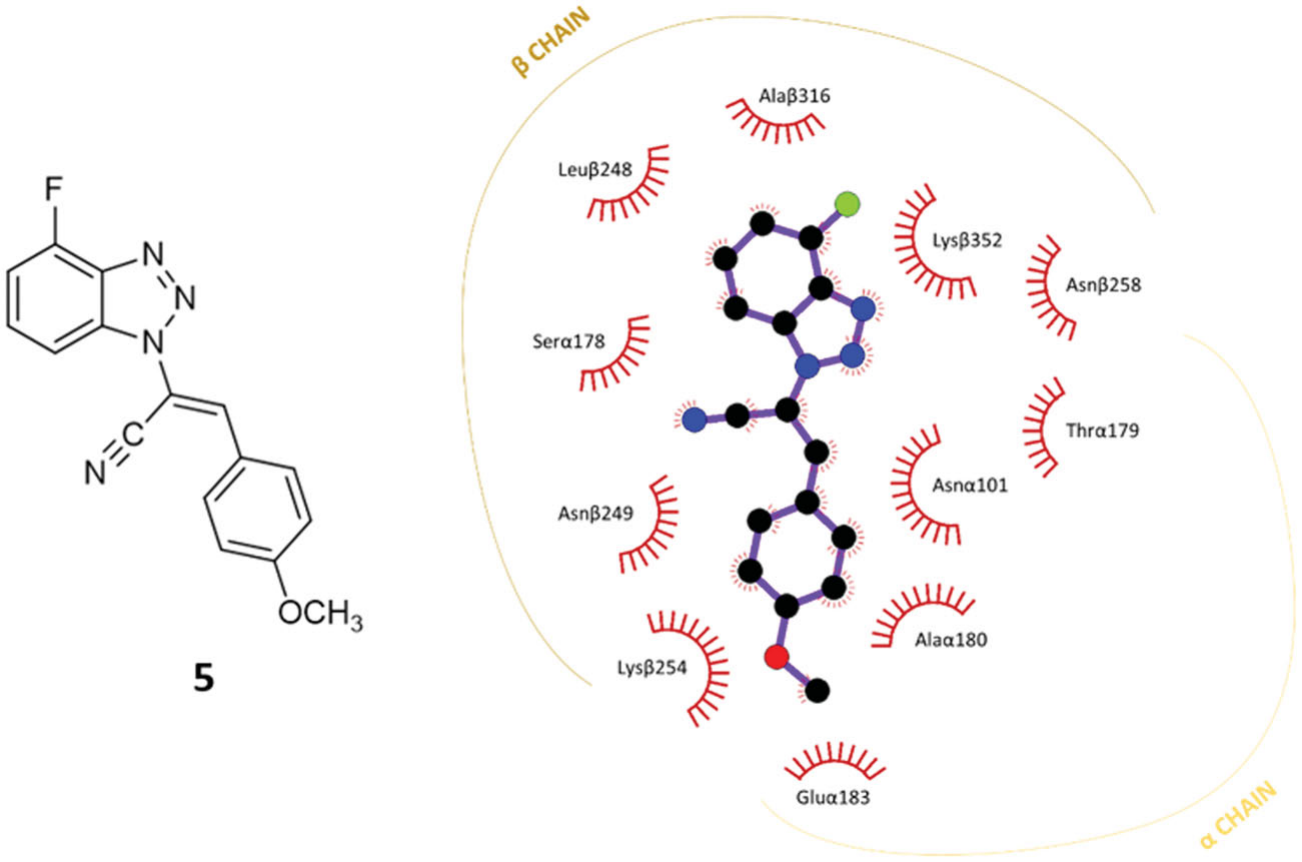
2D-representation of the best-docked pose of compound **5** and its nonpolar interactions with the amino acids (here labelled with the three-letters denomination, the belonging chain and the number of the protein secondary structure) of the binding pocket at the interface of α,β-tubulin. Picture made with LigPlot^+^.^35^ Hydrophobic interactions are depicted as hashes, for both protein and ligand atoms. Atoms are differently coloured: black (C), blue (N), green (F), and red (O).

A preliminary docking study was built on compound **5,** the 4′-fluoro parental compound of our former hit *p*-methoxy derivatives **34** and **9a** in the previously validated CBS.^30^ The docking simulation was conducted with AutoDock Vina.^31^ The top-ranked conformational pose of compound **5** revealed an affinity energy of −9.3 kcal⋅mol^−1^. It shows also notable stability in the other nine poses generated from the simulation, with average affinity energy of circa −9.2 kcal⋅mol^−1^. The re-docked best-ranked pose of colchicine had affinity energy of −9.8 kcal⋅mol^−1^,^30^ hence they have a similar predicted affinity for CBS on tubulin. Notably, it is also higher than the affinity energy of previous hits **34** and **9a**, (–6.7 and −8.6 kcal⋅mol^−1^, respectively).^30^ This result pushed us to investigate in depth the predicted interactions between **5** and the CBS. The best-predicted pose of compound **5** generates different non-covalent interactions with the exposed amino acids of the tubulin-binding site, generating a different pattern of polar and nonpolar contacts in comparison to compound **9a**. The higher contribution to the protein-ligand affinity is the hydrophobic term. As shown in Figure 3, each of the main moieties constituting the ligand backbone is involved in nonpolar contacts with amino acids on both α- and β-subunits of tubulin. The 4′-fluoro benzotriazole scaffold establishes connections mainly with β-chain amino acids: Leuβ248, Alaβ316, Lysβ352 and Asnβ258. The acrylonitrile linker in compound **5** interacts mainly with Serα178 (C≡N) and Asnα101 (=CH). The phenoxy moiety undertakes several hydrophobic interactions with aa located at the interface of the two subunits: Asnβ249, Lysβ254, Gluα183, Alaα180, Asnα101.

**Figure 4.**
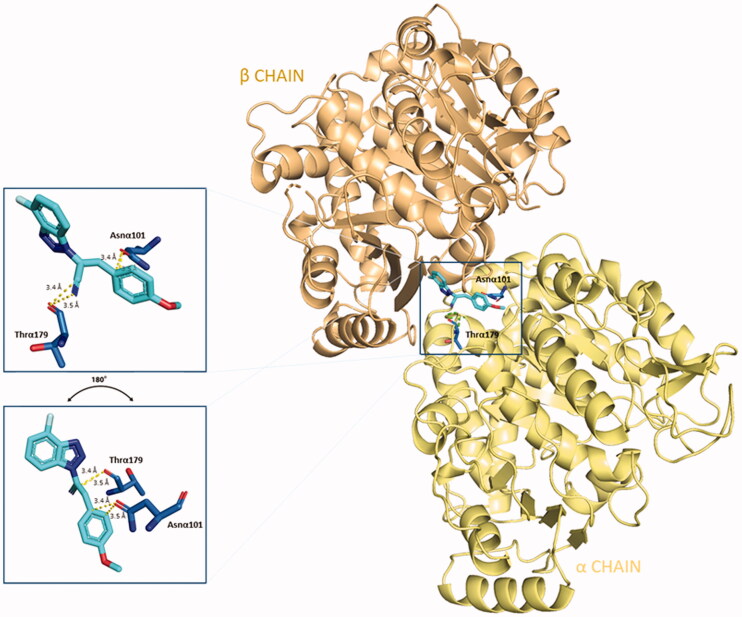
Three-dimensional representation of the binding conformation of compound **5** in CBS at the interface between α- and β-tubulin. Α-subunit is coloured in pale-yellow tint, β-one in light-orange tint. The ligand is in blue marine, interacting amino acids in blue. Yellow dashed lines consider the polar interactions, with a distance value in Å. Native 3D figures were produced via PyMOL.^33^

Apart from the consistent hydrophobic term, different polar contacts were recognised to contribute to the affinity, with α-subunit amino acids. A polar interaction is established between the cyan nitrogen of compound **5** and the carbonyl oxygen of Thrα179 (3.5 Å), while a polarised C⋅⋅⋅O interaction can be identified between the cyan carbon and the carbonyl oxygen of Thrα179. The lateral amide oxygen of Asnα101interact both with the C–1″ and C–2″ of the lateral phenoxy ring (∼ 3.5 Å both) through polar aryl π-heteroatom interactions, comparable to an amide-π stacking where the C = O points towards the phenyl ring (bond energy ∼ −2 kcal⋅mol^−1^).^42^^,^^43^ Polar interactions are depicted in Figure 4. This docking prediction gives hints for an interesting mode of binding to the CBS on tubulin.

Given the good predicted profile of binding of compound **5,** our work proceeded with the design of different analogues bearing the benzotriazole scaffold, the linked acrylonitrile chain (to each of the three triazole nitrogen atoms), and various aromatic moieties selected based on the activity profile of our previous MTAs.^30^ The final compounds are reported in Table 1. Introductory pharmacokinetic and drug-likeness properties were predicted for the newly designed series of compounds (**5**–**13**) that were subjected to a SwissADME prediction (http://www.swissadme.ch) also reported in Table 1 and the full prediction output is listed in the SM.

**Table 1.** Chemical structure of the final compounds (**5**–**13**) and pharmacokinetic and drug-likeness properties obtained by SwissADME prediction. 
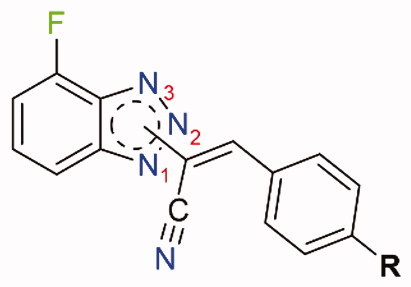

				[graphic]					
Label	Triazole substitution	R	Rotatable bonds	H-bond acceptors	Log *P*_o/w_	Solubility	GI absorption	BBB permeation	Drug-likeness
5	N-1	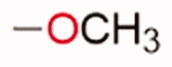	3	5	2.86	S-MS	High	Yes	Yes
6	N-1	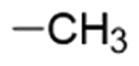	2	4	3.24	MS	High	Yes	Yes
7	N-1	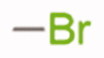	2	4	3.51	MS	High	Yes	Yes
8	N-2	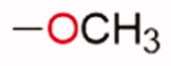	3	5	2.89	MS	High	Yes	Yes
9	N-2	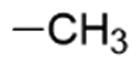	2	4	3.25	MS	High	Yes	Yes
10	N-2	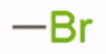	2	4	3.54	MS	High	Yes	Yes
11	N-3	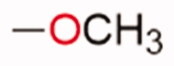	3	5	2.85	MS	High	Yes	Yes
12	N-3	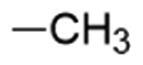	2	4	3.19	MS	High	Yes	Yes
13	N-3	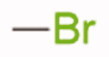	2	4	3.49	MS	High	Yes	Yes

GI: gastrointestinal; BBB: blood–brain barrier; S: soluble; MS: moderately soluble.

The designed derivatives have a molecular weight range of 278–343 g/mol, with a certain degree of rigidity (2–3 rotatable bonds). The designed molecules possess acceptor atoms for H-bonds, but not donors, as confirmed by the preliminary docking study. They showed a good profile of lipophilicity, with average Log*P* values of 2.85–3.54. All compounds are predicted as soluble or moderately soluble in water. As for the pharmacokinetic properties, in general, they are predicted to be highly absorbable from the gastrointestinal barrier and the blood–brain barrier. They seem also to be drug-like molecules, respecting Lipinski’s rule of five (0 violations). Figures S2–S4 in SM show the full SwissADME report for each compound. This hit optimisation process improved the predicted ADME properties. Comparing the ADME prediction of previous compound **9a**^30^ and new derivative **5** here reported, the latter were proved more drug-like than the parental compound. A lower predicted Log *p* values for ligand **5**, 2.86 while **9a** Log *P* was calculated at 3.53.^30^ Compound **5** is predicted to be a lead-like compound, with a Log *P*_o/w_ < 3, unlike the previous hit **9a**.

Given the satisfying virtual results obtained by docking and ADME predictions, we proceeded in synthesising this promising series of compounds.

### Chemistry

Scheme 1 depicts the synthetic route for the designed compounds **5**–**13**. The benzotriazole derivative **1** was obtained as previously reported.^41^ Compound **1** was used as a starting material to obtain three geometric isomers (**2**–**4**) bearing an acetonitrile chain on each of the three nitrogen atoms on the triazole ring. The reaction was conducted under basic conditions, by potassium hydroxide (KOH). Acetonitrile intermediates **2**–**4** were fully characterised, N-1 isomer (compound **2**) was identified through NOESY NMR spectra, while isomers N-2 and N-3 were identified by chemical shift of the CH_2_ carbon at ^13 ^C NMR spectra, see Figures S8 and S10 in the Supplemental Material. The stereochemistry of the double bond was identified as *Z* through ^1^H- and ^13 ^C NMR spectra, as already established and described.^30^ The chemical shift of the ^1^H and ^13 ^C for the CH moiety clearly distinguish if the *Z* or *E* isomer is obtained.

Following Knoevenagel condensation reactions with four different aromatic aldehydes (**I**–**IV**) gave final benzotriazolacrilonitrile compounds **5**–**7**, **8**–**10,** and **11**–**13** as single E isomer, identified by NMR, as previously described.^30^ Derivatives **5**–**7** were obtained *via* Knoevenagel condensation of **2** and the proper aldehydes **I**–**IV**. Derivatives **8**–**10** were synthesised by reaction of **2** and **I**–**IV**, while intermediate **3** and the aldehydes **I**–**IV** led to compounds **11**–**13** through the same synthetic approach. Knoevenagel reactions were conducted in three different conditions: (1) compounds **9**, **10** and **12** were yielded by using dimethylamine (DMA), as base and catalyst; (2) dimethylammonium dimethylcarbamate (DIMCARB) was employed as a catalyst to furnish derivatives **5** and **7**, and (3) compounds **8**, **11,** and **13** were obtained utilising Cs_2_CO_3_ to alkalise the reaction environment.

### Biology

#### Antiproliferative activity: NCI60 in vitro screening

As first antiproliferative activity evaluation, all synthesised compounds were subjected to the *in vitro* screening offered by the National Cancer Institute of Bethesda (USA, https://dtp.cancer.gov). The molecules were tested at the initial concentration of 10 µM on the complete panel of cancer cell lines which comprises 60 lines of solid and haematological tumours. All the derivatives with a remarkable antiproliferative activity at 10 µM, were further tested on the same panel, at five different dilutions ranging from 100 µM to 10 nM. Complete NCI60 results for compounds **5**–**13** are reported in Figures S29–S44 of the Supplemental Material section.

#### Structure-Activity relationships (SARs) report

**Figure 5.**
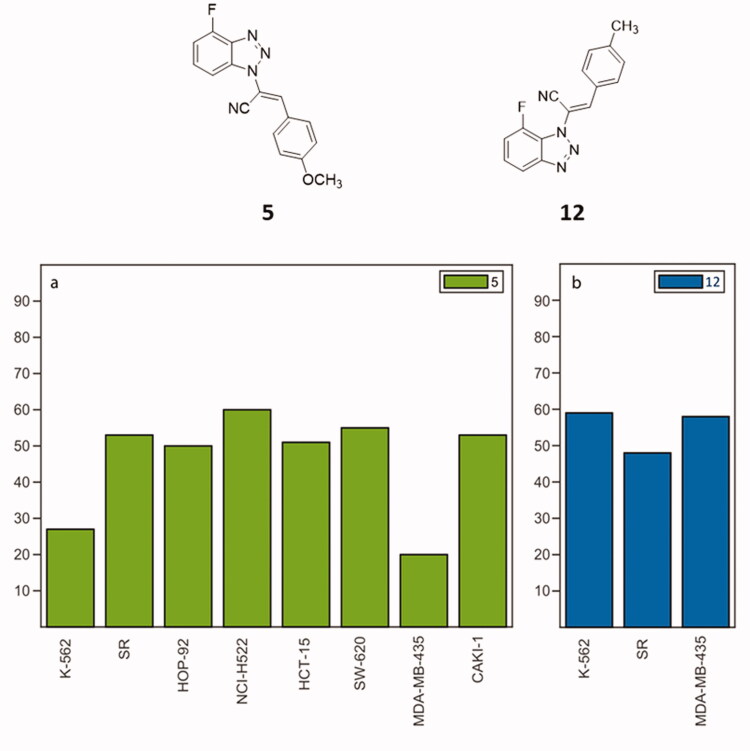
NCI60 cancer analysis on compounds **5** (a) and **12** (b). The histograms illustrate %GI of cancer cells elicited by **5** (green) and **12** (blue) at 100 nM.

Among the tested compounds, all the N2-derivatives **8**–**10** showed a low activity on the growth inhibition of most of the NCI cancer cell lines. N3-substituted molecules **11**–**13** and N1-derivatives **5**–**7**, showed appealing values of growth inhibition (GI). Considering the N3-derivatives, at 10 µM, compound **11** showed strong antiproliferative activity against all cancer cell lines, displaying GI ranging from 70 to 100%, and showing cytotoxicity against 21 of the 60 cancer cell lines. GI percentages were also calculated for nine cancer cell lines at 1 µM, showing cytotoxicity against three cell lines. Compound **13** elicited the most cytotoxic effect of this series at 10 µM with an intense cell growth inhibition on almost all the tested cell lines along with a strong cytotoxic activity on 57 out of 60 cell lines. Decreasing the concentration to 1 µM, compound **13** still inhibited twelve cancer cell lines (GI from 70 to 100%), also showing cytotoxicity against two of them. As for compound **12**, it revealed an overall growth inhibition and cytotoxic activity comparable with parental compounds **11** and **13**. At 1 µM concentration, it exerted the best antiproliferative effect against all leukaemia cell lines, while at 100 nM was proved strongly active against three cancer cell lines: K–562, SR (leukaemia) and MDA–MB–435 (melanoma) as shown in Figure 5. As concerns N1-derivatives, compounds **5**–**7** displayed a general improved antiproliferative profile compared to the N2- and N3-derivatives. Compound **7** showed percentages of cancer cell growth inhibition around 80–100% at 10 µM, with a lethal effect on 48 of the 60 cancer cell lines. At 1 µM, derivative **7** displayed a cytostatic effect on 10 different cancer cell lines, while at 100 nM, the cytostatic effect resulted in a GI of 50% on the HOP–92 NSCLC cell line. When it comes to compound **6**, at 10 µM, it displayed a GI of 80–100% involving 53 cancer cell lines of the 9 types of tumours, but also a cytotoxic effect on 24 cell lines. As for its activity at 1 µM, a < 50% reduction of GI can be identified against 52 cancer lines, particularly for leukaemia and melanoma and the cytotoxicity effect was proven against 8 cell lines. Compound **6** is also active at 100 nM on different tumour cell lines such as K-562 (leukaemia, growth percentage 40%), and MDA-MB-435 (melanoma, 20%). The *p*-OCH_3_ substituted 1-derivative **5** showed an overall proliferation inhibition ranging from 75 to 100%, with a limited cytotoxic effect. The antiproliferative effect is kept at a concentration of 1 µM: derivative **5** has a general detectable cytostatic effect on the entire NCI60 panel, also exhibiting cytotoxic effect against six lines. Remarkably, an 80% GI was registered against the K–562 leukaemia cell line when derivative **5** was administered at 100 nM concentration. The reduced GI on eight cancer cell lines induced by **5** at 100 nM, is charted in Figure 5. Given these results, compounds **5** and **12** were selected as the most effective molecules and subjected to further biological assessment.

#### Antiproliferative activity of compounds against HeLa cells

**Figure 6.**
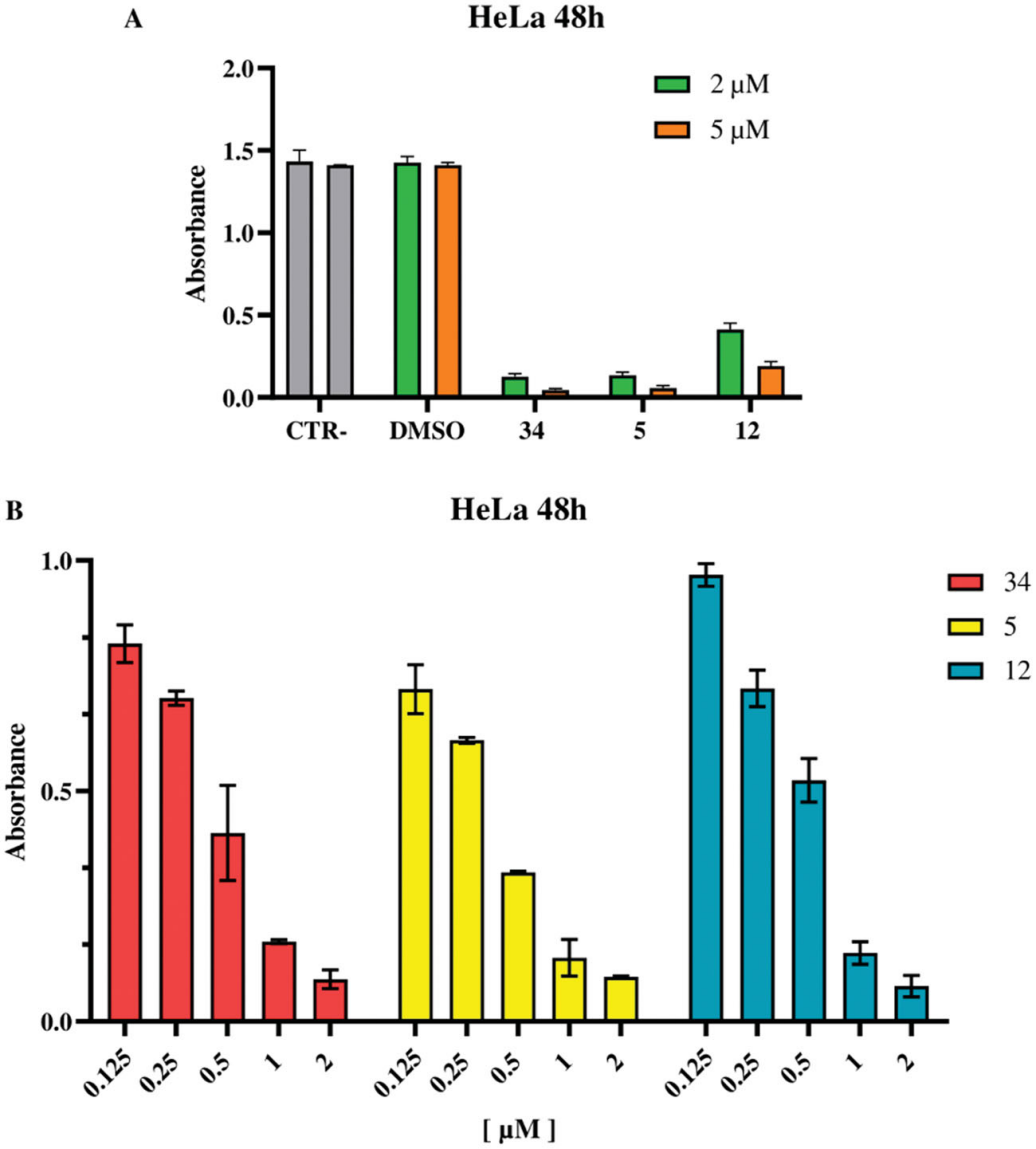
Inhibition of HeLa cell proliferation *in vitro*, as determined by the XTT assay. (A) HeLa cells were incubated with DMSO (vehicle control) and with compounds **34** (positive control) **5** and **12** at 2 and 5 μM concentrations. (B) HeLa cells were treated with compounds **34**, **5** e **12** at different concentrations in a range between 0.125–2 μM. All experiments were performed in triplicate.

Further analyses were carried out using the HeLa cell line, as previously validated.^30^ To calculate the IC_50_ values for the newly synthesised compounds **5**–**13**, an XTT assay was performed in triplicate on HeLa cells after 48 h of treatment, Table 2. Cells were treated with all compounds at different concentrations ranging from 2 to 5 μM, and DMSO was used as a control. Compounds **5** and **12** showed more pronounced antiproliferative activity and were used at concentrations of 0.125 to 2 μM, while compound **34** was used as an in-house positive control (Figure 6). A colorimetric assay was performed after 48 h of treatment, absorbance data were obtained and processed to calculate the percentage viability of the cells. A 50% reduction in live cells was detected and IC_50_ values were calculated. Compounds **5** and **12** turned out as the most promising with IC_50_ values of 0.6 μM and 0.45 μM, respectively.

**Table 2.** Cytotoxic activities of the new compounds **5**–**13** as given by IC_50_ values in HeLa cells.

Compound	IC_50_ HeLa
**5**	0.60 μM
**6**	>10 μM
**7**	>10 μM
**8**	>10 μM
**9**	>10 μM
**10**	>10 μM
**11**	>10 μM
**12**	0.45 μM
**13**	>10 μM

#### Mechanism of action of compounds 5 and 12 against HeLa and MCF-7 cells

**Figure 7.**
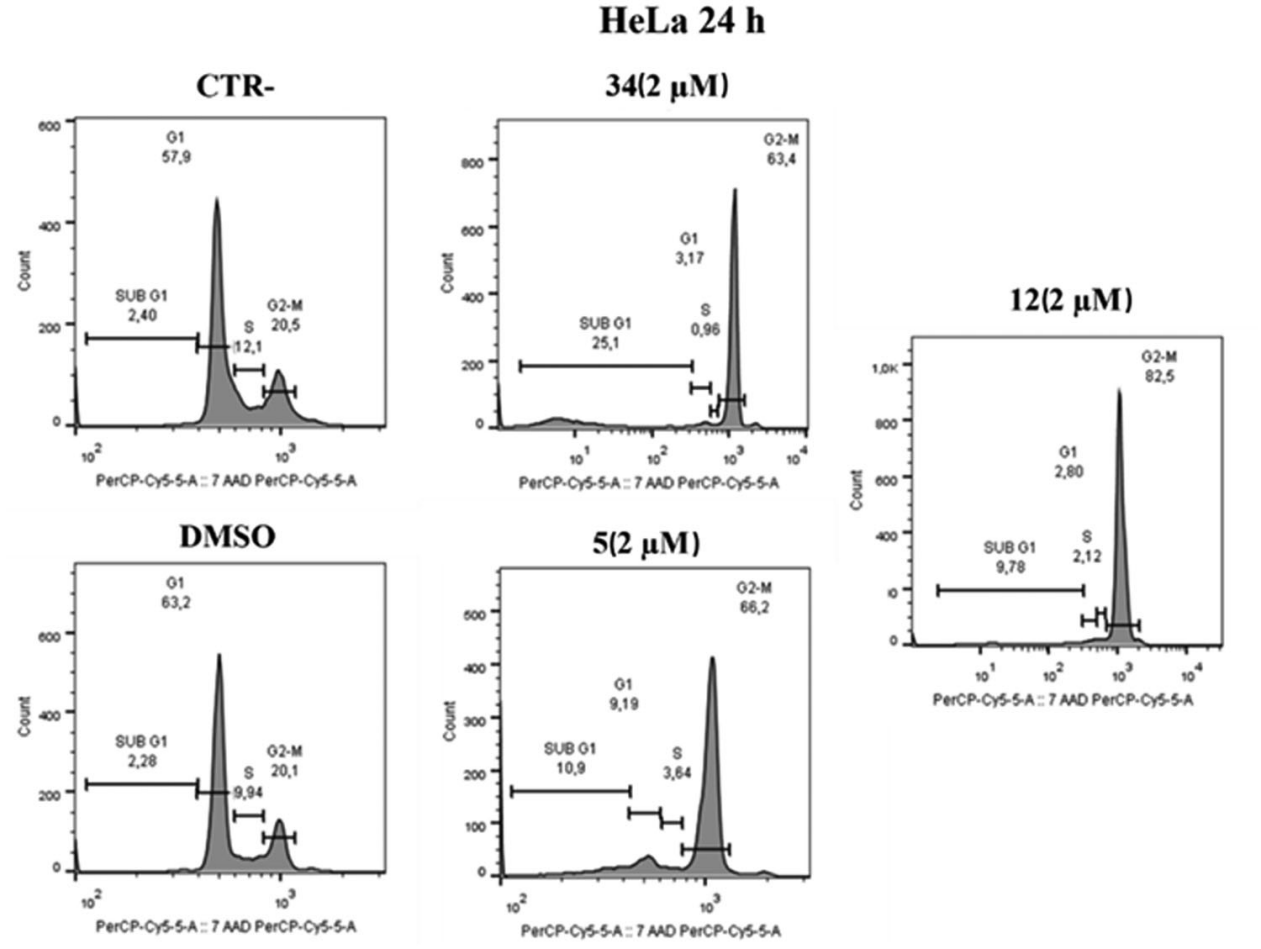
Distribution of HeLa cell in cell cycle assay. At 2 μM, both compounds **5** and **12** cause cell blockade in the G2/M phase compared to control and DMSO after 24 h. Compound **34** was used as a positive control.

To prove that compounds **5** and **12** can hijack the cell cycle, flow cytometry was performed to assess changes in DNA content after treatment on HeLa and MCF-7 cells. As shown in Figure 7, administration of compounds **5** and **12** at 2 μM over 24 h caused an increase in the number of cells in the G2/M phase in HeLa cells; our previous hit compound **34** was used as an in-house positive control. Specifically, an increase (82.5%) was observed in compound **12** treated cells compared to control (20.5%) and to DMSO (20.1%) at 2 µM concentrations. Furthermore, the behaviour is very similar to the positive control under the same conditions (63.4%). Similarly, in compound **5** treated cells, an increase (66.2%) in the G2/M phase was observed at a 2 µM concentration.

**Figure 8.**
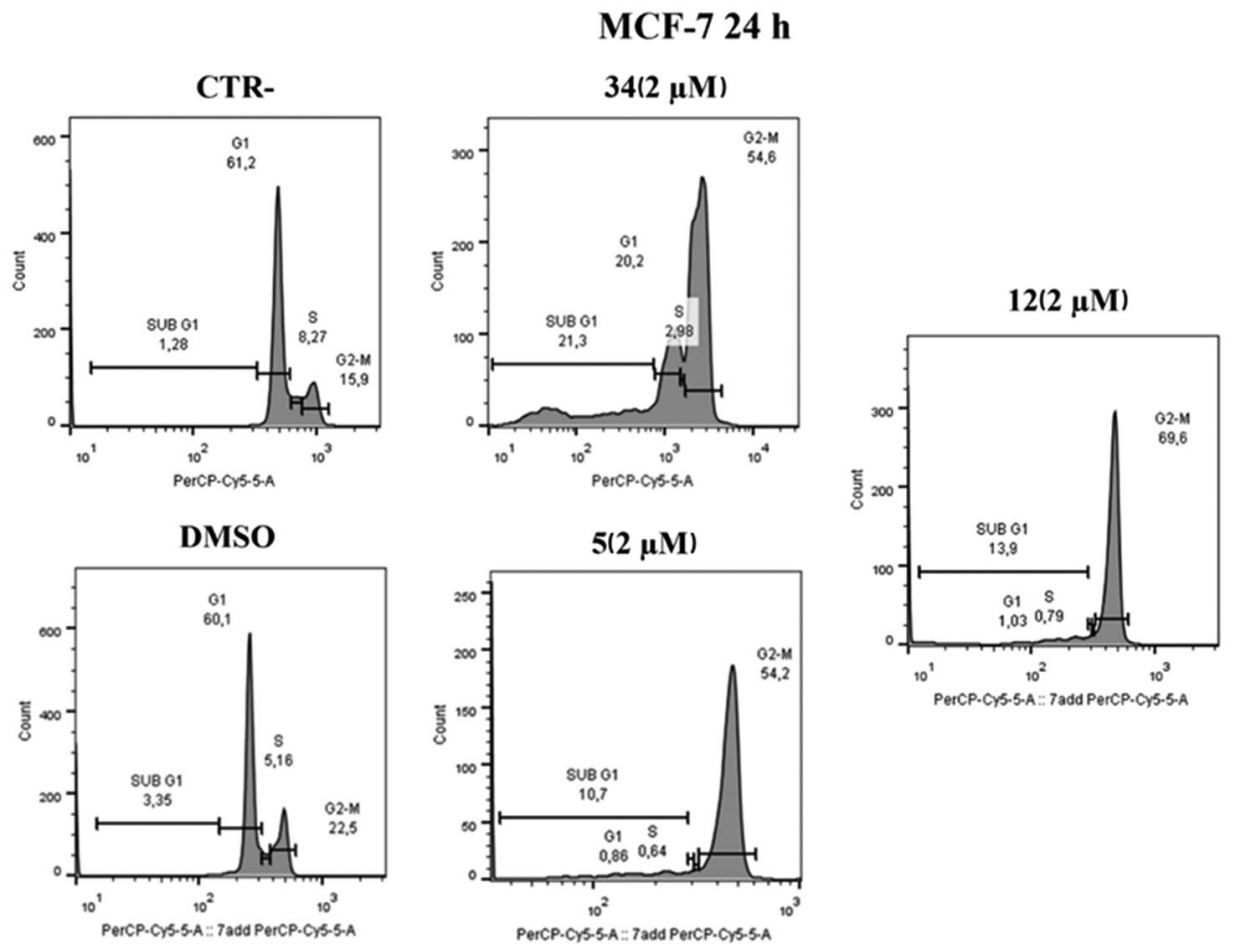
Cell cycle distribution after administration of compound **5** or **12**, at 2 μM after 24 h. At a concentration of 2 μM, compounds **5** and **12** cause cell blockage in the G2/M phase compared to control and DMSO. Compound **34** was used as a positive control.

Similar results were obtained on MCF-7 cell lines after 24 h at 2 µM. As shown in Figure 8, in MCF-7 cell lines treated with compound **5** at the 2 µM concentration, an increase in G2/M phase (54.2%) was observed, similarly to the positive control. The same effect is evident in cells treated with compound **12** where an increase of 69.6% was observed.

#### Annexin V assay uncovers apoptosis activation in HeLa and MCF-7 cell lines after treatment with compounds 5 and 12

**Figure 9.**
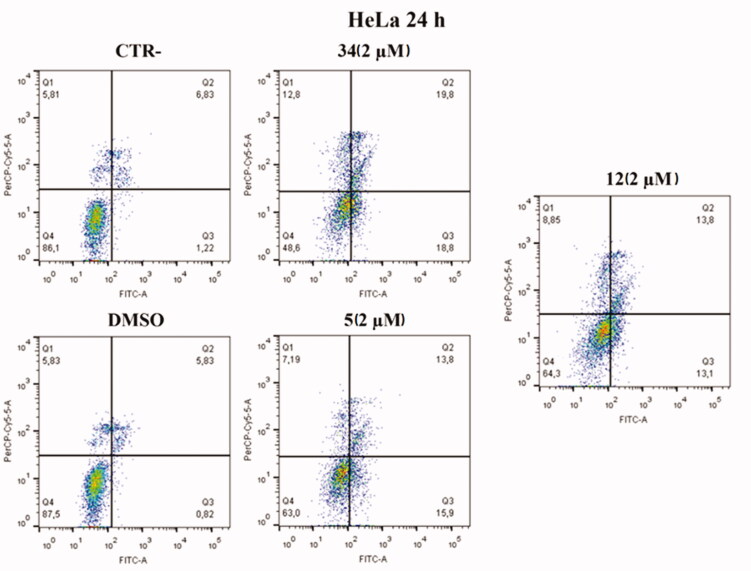
Annexin V analysis of HeLa cell lines after treatment with 2 µM of compounds **5** and **12** after 24 h. DMSO and compound **34** were used as controls. Apoptotic, necrotic, and live cells were stained with Annexin V FITC/7-AAD and then analysed by BD FACS DIVA software.

Based on cell cycle assay and XTT results, Annexin V–FITC/7-AAD double staining kit was used in flow cytofluorimetric analyses. HeLa cells were treated with compounds **5** and **12** at 2 µM concentration. After 24 h, cells were stained with both FITC and 7-AAD and analysed with BD FACS CANTO II. Compounds **5** and **12** activated apoptosis (Figure 9). After 24 h of treatment with compound **5**, the percentage of late apoptotic cells increased from 6.83% in control cells and 5.83% in cells with DMSO to 13.8% in treated cells. Besides, an increase in early apoptosis was noticeable, with compound **5** reaching rates of 15.9% compared to control and DMSO, 1.22% and 0.82%, respectively. Compound **12** caused late apoptosis in 13.8% of cells and early apoptosis in 13.1%. Both values approximate the percentage of cells in late apoptosis 19.8% and early apoptosis 18.8% caused by compound **34**, which was used as an in-house positive control.

**Figure 10.**
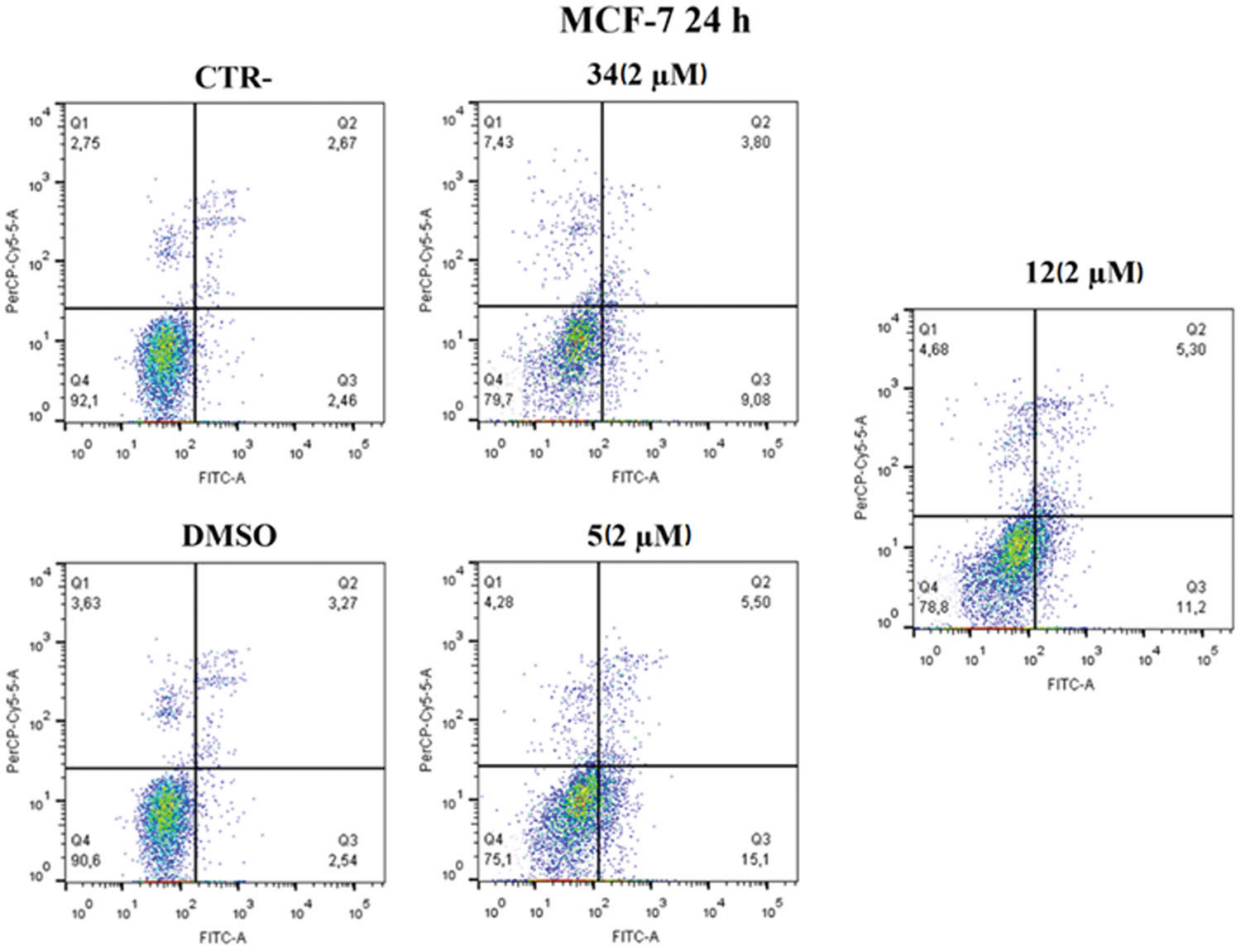
Annexin V analysis of MCF-7 cell line after administration of compounds **5** and **12** (2 µM) after 24 h. DMSO and compound **34** were used as controls. Apoptotic, necrotic, and live cells were stained with Annexin V FITC/7-AAD and then analysed by BD FACS DIVA software.

An increase in early apoptosis is observed in MCF-7 cell lines (Figure 10). The percentage of cells in early apoptosis increased by 15.1% with compound **5** and 11.2% with compound **12** compared to control and DMSO, 2.46% and 2.54% respectively. Both values are higher than the percentage of early apoptosis caused by compound **34** (9.08%) used as an in-house positive control.

#### Compounds 5 and 12 cause PARP-1 cleavage in HeLa and MCF-7 cell lines

**Figure 11.**
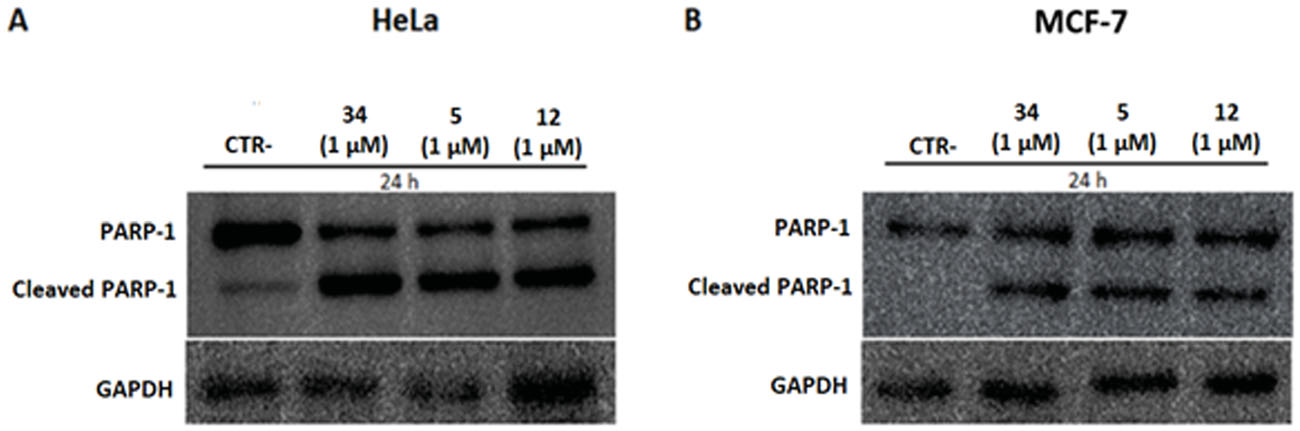
Protein levels of PARP-1 and cleavage of PARP-1 in HeLa and MCF-7 cell lines after treatment with compounds **5** and **12** at 1 µM. Caspase-mediated cleavage of PARP1indicated activation of cell death by apoptosis in both cell lines. DMSO and compound **34** were used as negative and positive controls, respectively. All results were normalised using GAPDH.

To investigate the mechanism of action of compounds **5** and **12** on HeLa and MCF-7 cells, the latter cells were treated with concentrations of 1 µM and the protein expression levels of PARP-1 and its cleaved form were measured. PARP-1 is a protein involved in the apoptotic cycle of a cell because it is the substrate of several “suicidal” proteases, such as caspases. The proteolysis of PARP-1 generates multiple cleavages in fragments, which are biomarkers to trace the proteases’ implication in cell death.^44^ Twenty-four hours after treatment, pellets were collected, and western blotting was performed. As shown in Figure 11, treatment with compounds **5** and **12** caused PARP-1 cleavage and generated an 89-kDa fragment on both cell lines, indicating the activation of the apoptotic pathway. The 89-kDa fragment of PARP-1 resulting from caspase cleavage is considered an early apoptosis hallmark, therefore these results confirm the pro-apoptotic activity of compounds **5** and **12** observed in the Annexin V assay.

When targeting tubulin and cell replication cycle, an antiproliferative but not cytotoxic activity may be desirable to target the fast-replicating tumour cells rather than normal cells. Given the comprehensively satisfying activity and the poorer cytotoxic effect, compound **5** was selected as the most promising compound and it was employed for further biological assessments to confirm the mechanism of action and to highlight its implication in different tumour pathways.

#### Compound 5 affects microtubule formation in HeLa cells

**Figure 12.**
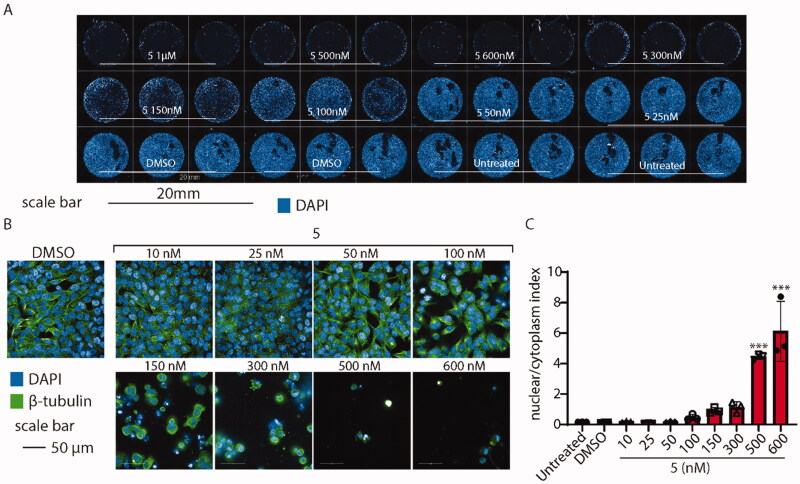
High-content confocal screening performed on HeLa cells. (A) Overview of an acquisition performed at 5×, nuclei are stained in blue (DAPI), scale bar 20 mm. (B) Exemplificative images of HeLa cells stained for β-tubulin (green) and nuclei (DAPI) treated or not with compound **5** at different concentrations. (C) Statistical analysis of nuclear/cytoplasm area of HeLa cells shown in B, performed using one-way ANOVA. Data are expressed as mean ± SD *N* = 3, α = 0.5 ***p < .0001.

To probe whether microtubules might be affected by the administration of compound **5**, we observed their structure on HeLa cells exposed to various concentrations of the drug for 24 h. Then, we analysed cells using high-content confocal microscopy. As shown in Figure 12(A,B), compound **5** affects the shape and vitality of cells when administered above 100 nM. To better elucidate the cytoskeleton displacement, we tested whether compound **5** might change the ratio between the nuclear/cytoplasm dimension, segmenting the shape of every cell detected by the screening. As expected, the analysis revealed that, when compound **5** is administered above 100 nM, the cytoskeleton collapses leading to cell shrinkage, increasing the nuclear/cytoplasm ratio by ∼ 6 times (Figure 12(C)).

#### Co-administration of compound 5-doxorubicin: compound 5 increases cytotoxicity of doxorubicin

**Figure 13.**
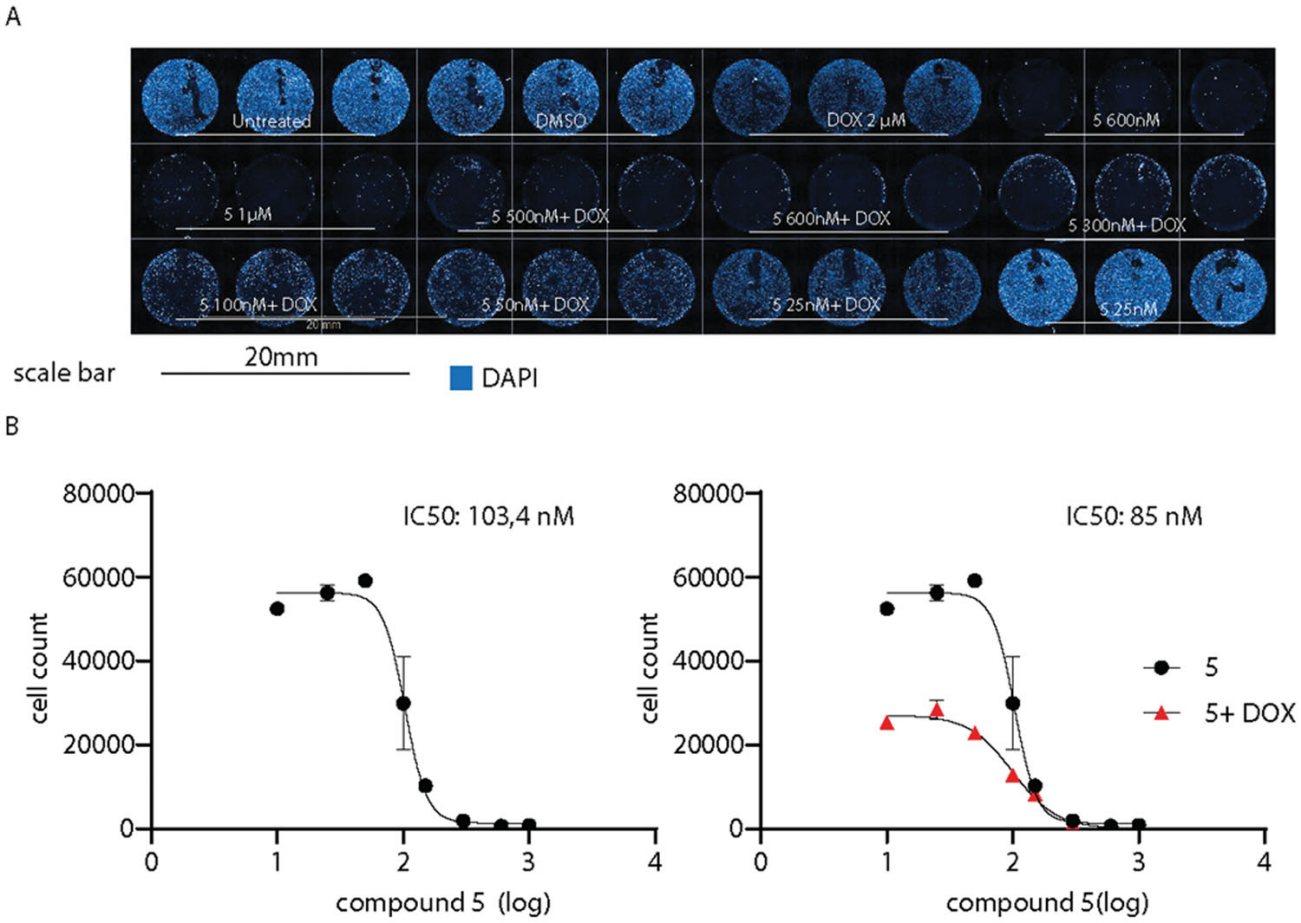
Administration of compound **5** decreases A375 melanoma cells viability. (A) Overview of high-content screening performed on A375 cells treated or not with compound **5** and doxorubicin. (B) IC_50_ of the sole compound **5** (black) or in association with doxorubicin (red). IC_50_ values were calculated by counting the number of nuclei in every experimental condition. Compound **5** IC_50_ when co-administered with doxorubicin is decreased from 103.4 to 85 nM.

Then, it was determined to investigate whether the administration of compound **5** might enhance the antitumor activity of canonical antineoplastic drugs. Therefore, co-administration with doxorubicin was probed, as a well-established drug used to treat a wide variety of cancers including leukaemia, breast, lung, and ovary cancer. The co-administration of derivative **5** plus the standard drug were tested on A375, a cell line derived from an aggressive melanoma with an invasive phenotype resistant to clinical doxorubicin treatments.^37^ A375 cells were exposed to doxorubicin alone at 2 µM for 24 h, a concentration that resembles the plasmatic concentration of the drug during high-dose chemotherapies.^45^ As shown in Figure 13(A,B), while doxorubicin alone did not affect A375 cells when administered alone, the co-administration with compound **5** dramatically affects A375 cell viability, decreasing compound **5** IC_50_ from 103.4 to 85 nM.

#### Binding site confirmation

##### Compound 5 interferes with tubulin assembly and competes with colchicine binding

**Figure 14.**
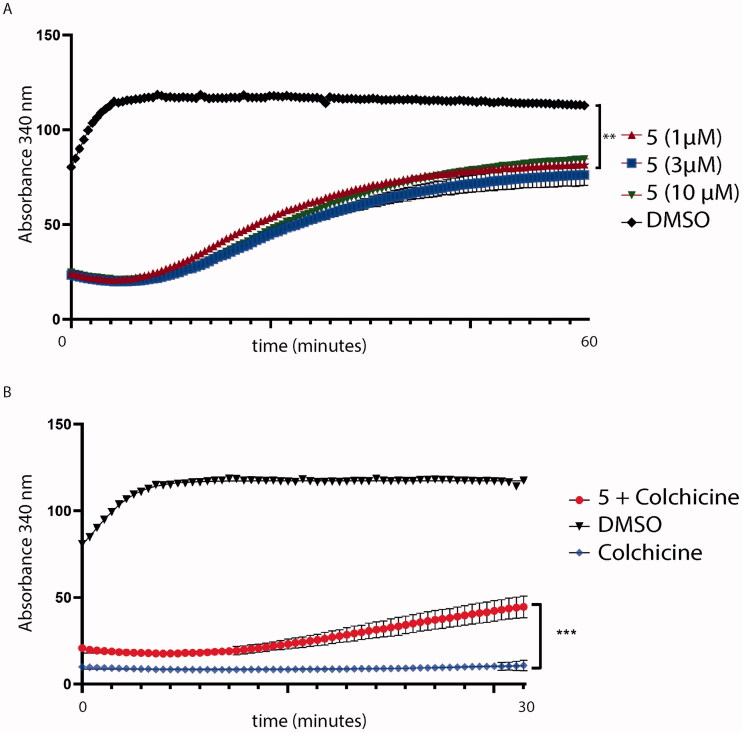
(A) Tubulin polymerisation assay. Tubulin was pre-incubated with compound **5** at different concentrations. Then, tubulin assembly was assessed by measuring absorbance every 2 s for 60 min at 340 nm. Compound **5** (red lines) decreases tubulin polymerisation at every concentration tested (1, 3 and 10 μM); (B) Colchicine-competition assay. Tubulin was pre-incubated with an equimolar concentration of colchicine with compound **5** at 1 μM. Then, tubulin polymerisation was assessed by measuring absorbance every 2 s for a total of 30 min. Statistical analyses were performed using a one-sample T test and expressed as mean ± SD, ***p ≤ .001.

To confirm the mechanism of action, compound **5** was assessed to prove whether it might interfere with tubulin assembly decreasing tubulin polymerisation. As shown in Figure 14(A), compound **5** affects tubulin polymerisation, if compared to tubulin treated with the sole DMSO. Then, to understand if compound **5** might bind tubulin in the CBS, we assessed a colchicine competition assay, as reported in Figure 14(B). As previously predicted by docking simulation, compound **5**, administered at equimolar concentration with colchicine, decreases colchicine effectiveness on tubulin polymerisation (*p* < .001 red curve, Figure 14(B)). Colchicine and DMSO were used as control.

##### Compound 5 is predicted to compete with colchicine at the interface of α- and β-tubulin

**Figure 15.**
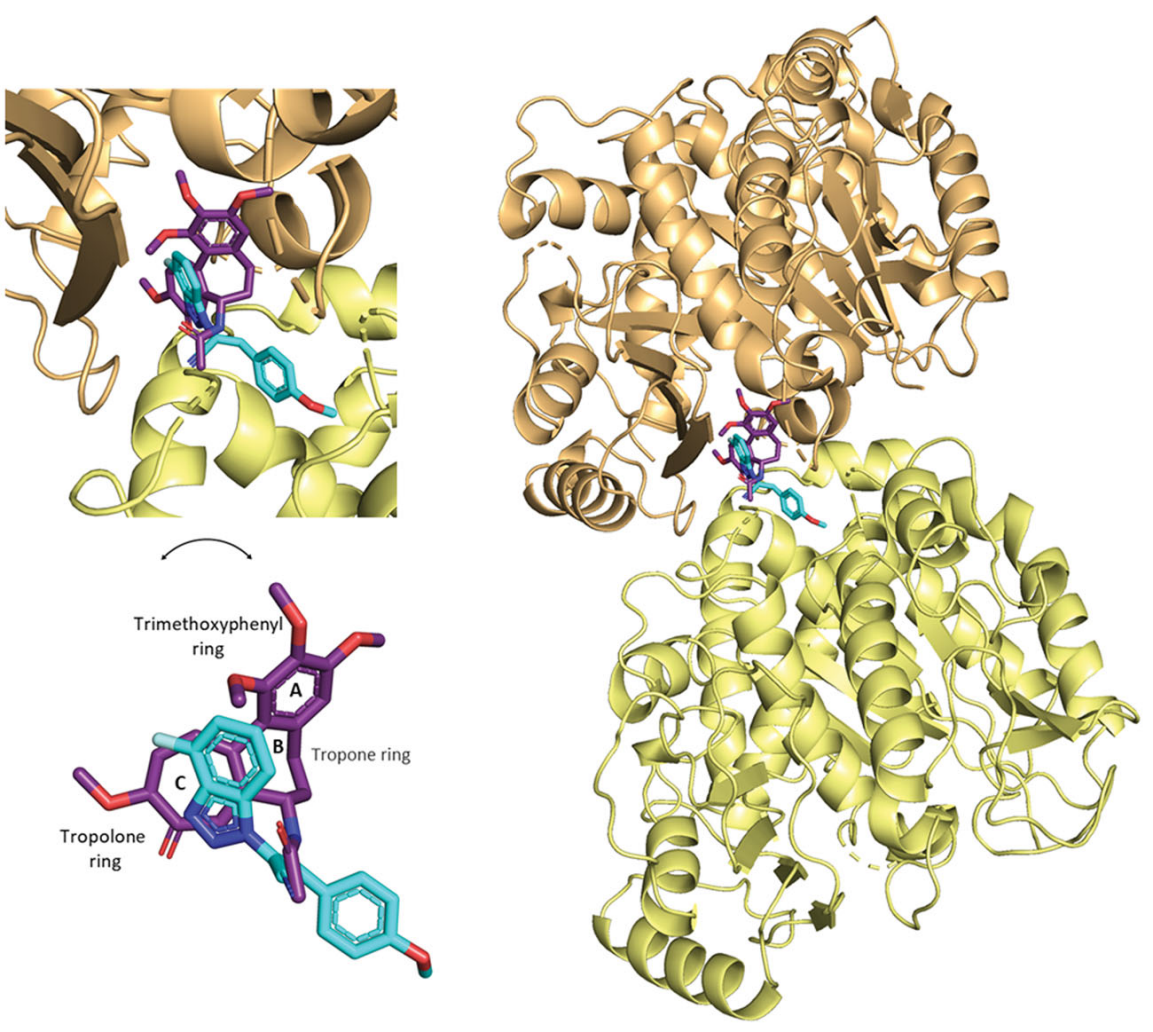
Superimposition of the predicted conformation of compound **5** (coloured in blue marine) and the crystal structure of colchicine (in violet-purple, PDB ID: 4O2B).

The binding conformation and interactions of compound **5** were already displayed before. Here, the crystal structure of colchicine in its binding pocket on tubulin (PDB ID: 4O2B), was superimposed with the best-predicted pose of compound **5** (Figure 15), as described in Section 3.1, to highlight the different binding in the same spatial environment and to support the experimental results gained from the colchicine-compound **5** competition assay, previously reported.

By the superimposition of the two structures, they clearly occupy the same specific portion at the interface of α- and β-tubulin. The 4′-fluoro benzotriazole moiety is predicted to sit in the specific space occupied by the crystal conformation of the tropolone ring of colchicine. The cyan group of the acrylonitrile linker directs in the same way as the amide group linked to the tropone ring. The *p*-OCH_3_ phenyl structure does not overlap with any moiety of colchicine, but points towards the interface-exposed part of the α-chain, interacting with its amino acids, as already shown.

This prediction shows the two structures bind with a good affinity profile, confirming the experimental competition between the two tubulin inhibitors and the binding to CBS on tubulin.

##### Compound 5 is predicted to stabilise its binding in CBS by solvent-mediated interactions

**Figure 16.**
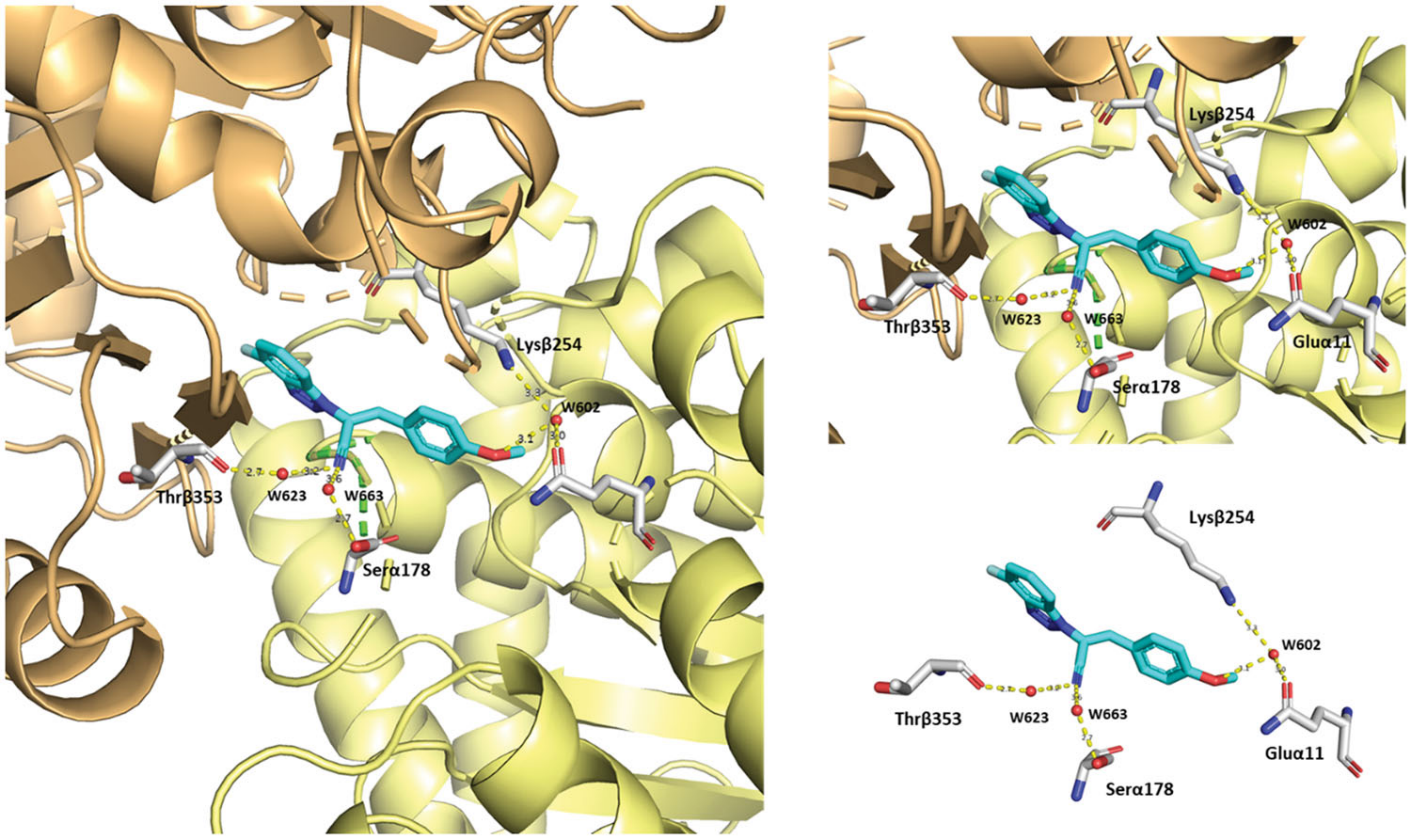
Representation of the solvent-mediated polar interactions established by compound **5** (blue marine). Water molecules (W623–W663–W602) are simplified by red spheres.

Compound **5** is predicted to establish several polar interactions with the exposed amino acids of CBS on tubulin (from both α- and β-tubulin). Some of them are mediated by water molecules. Notably, the major contribution in terms of water bridges is provided by the C≡N group and the methoxy oxygen in the ligand, see Figure 16. The cyan group contacts Thrβ353 and Serα178 via two water bridges. The methoxy oxygen reaches both Lysβ254 and Gluα11 with the W602 water molecule. These additional polar interactions contribute to building the predicted unique binding mode of compound **5** to α- and β-tubulin interface.

## Conclusions

As a background for this work, CBSI compounds **34** and **9a** emerged for their remarkable antiproliferative activity and their effect on the cancer cell. In this paper, a single fluorine atom was introduced in C–4′ of benzotriazole scaffold, a position not investigated before, and a series of fluorinate compounds bearing the acrylonitrile chain on N–1,2,3 on the benzotriazole ring (**5**–**13**) was designed, *in silico* validated and synthesised. The synthetised compounds were assessed through a preliminary *in vitro* screening on a panel of 60 cell lines of 9 tumours. Compounds **5** and **12** were selected for further biological investigations, as they inhibited cancer cell growth on several lines at 100 nM. Further proliferative assay performed on Hela cells, showed IC_50_ values of 0.6 and 0.45 µM for compounds **5** and **12**, respectively. Flow cytometry assay on both HeLa and MCF-7 cells confirmed these molecules interfere with the cell cycle showing a consistent blockade of cells in the G2/M phase. Pro-apoptotic activity of **5** and **12** was proved through the Annexin V assay resulting in an increase of early and late apoptotic cells, both in HeLa and MCF-7 cell lines. The pro-apoptotic activity of compounds **5** and **12** was confirmed by WB-analysis of PARP-1 and cleaved PARP-1,. Globally, the collected results suggested compound **5** as the most promising compound. It was further investigate for cytoskeleton collapse leading to severe cell shrinkage observed through a high-content screening and also for the competition with colchicine at the CBS on tubulin, showing a decreases the effectiveness of colchicine on the inhibition of tubulin polymerisation proving the interaction with the same binding site. To validate the latter result, the best-docked pose of compound **5** was superimposed with the crystal structure of colchicine. They showed to bind in the same specific portion of the CBS, but they did not show overlapping chemical portions between themselves, suggesting that compound **5** may undertake a different and novel pattern of interactions. Finally, co-administration of **5** and doxorubicin was assessed on the A375 cell line, a doxorubicin-resistant melanoma cell line. As expected, doxorubicin is not effective alone, while the co-administration exerts a synergic activity lowering the **5** IC_50_ value from 103 to 85 nM. In conclusion, the 4′-fluoro substitution of benzotriazole-acrylonitrile scaffold brought us a step forward in the optimisation process on previous hits **34** and **9a**; among the new derivatives synthesised, compound **5** was proved to be a good antiproliferative agent acting as promising MDA at nanomolar concentration.

## Supplementary Material

Supplemental Material
