# Supplementary material for: Design, synthesis, and biological screening of a series of 4′-fluoro-benzotriazole-acrylonitrile derivatives as microtubule-destabilising agents (MDAs)
Source: J Enzyme Inhib Med Chem. 2022 Aug 17;37(1):2223–40. doi: 10.1080/14756366.2022.2111680 (PMC9397482; doi:10.1080/14756366.2022.2111680)
Supplement: Supplemental Material [file IENZ_A_2111680_SM8735.pdf]

# Supplemental Material

# Design, synthesis and biological screening of a series of 4'-fluoro-benzotriazole-acrylonitrile derivatives as microtubule-destabilizing agents (MDAs).

Federico Riu[a], Roberta Ibba[a,*], Stefano Zoroddu[b], Simona Sestito[c], Michele Lai[d,e], Sandra Piras[a], Luca Sanna[b], Valentina Bordoni[b], Luigi Bagella[b,f] and Antonio Carta[a]

[a] *Department of Medicine, Surgery and Pharmacy, University of Sassari, via Muroni 23/a, 07100, Sassari, Italy.*

[b] *Department of Biomedical Sciences, University of Sassari, viale San Pietro 43/b, 07100, Sassari, Italy.*

[c] *Department of Chemical, Physical, Mathematical and Natural Sciences, University of Sassari, via Vienna 2, 07100, Sassari, Italy*

[d] *Retrovirus Centre, Department of Translational Medicine and New Technologies in Medicine and Surgery, University of Pisa, Strada Statale del Brennero, 2, Pisa, Italy.*

[e] *CISUP – Centre for Instrumentation Sharing – University of Pisa, Lungarno Pacinotti 43, Pisa, Italy.*

[f] *Sbarro Institute for Cancer Research and Molecular Medicine, Center for Biotechnology, College of Science and Technology, Temple University, Philadelphia, PA, 19122, USA.*

** Corresponding author: ribba@uniss.it*

Table of Contents:

(1) Compound 12 docks at the interface between a- and b-tubulin……………………..2

(2) ADME predictions (SwissADME)…………………………………………...…….3-5

(3) $^{1}H$ and $^{13}C$ NMR characterization…………………………………….………..…..6-29

(4) NCI60 *in vitro* screening…………………………………………………..…….30-46

**Compound 12 docks at the interface between α- and β-tubulin**

Given the broad spectrum of antiproliferative activity shown for compound **12** on the NCI60 screening, its predicted mode of binding in the CBS was investigated. The estimated affinity energy for the top-ranked pose of compound **12** is –8.8 kcal·mol$^{-1}$, with average affinity energy for the 10 top-predicted poses of –8.4 kcal·mol$^{-1}$ and a ΔRMSD of ~ 2 Å between the poses. The polar term is dominated by the fluorine bond promoted by the fluorine bond on C–4' of the benzotriazole scaffold with the amide $NH_2$ of Asnβ258 (~ 3.2 Å length). The hydrophobic term is assured mainly by the β–chain, but also by some α–subunit amino acids. The fluorine-free portion of the benzotriazole backbone interacts with Alaβ250, Leuβ255 and Metβ259. Metβ259, together with Lysβ352, interacts with the acrylonitrile linker. The higher contribution for the hydrophobic term is displayed by the *p*-$CH_3$ phenyl moiety, contacting Lysβ352, Thrα179, Serα178, Alaα180 and Leuβ248.

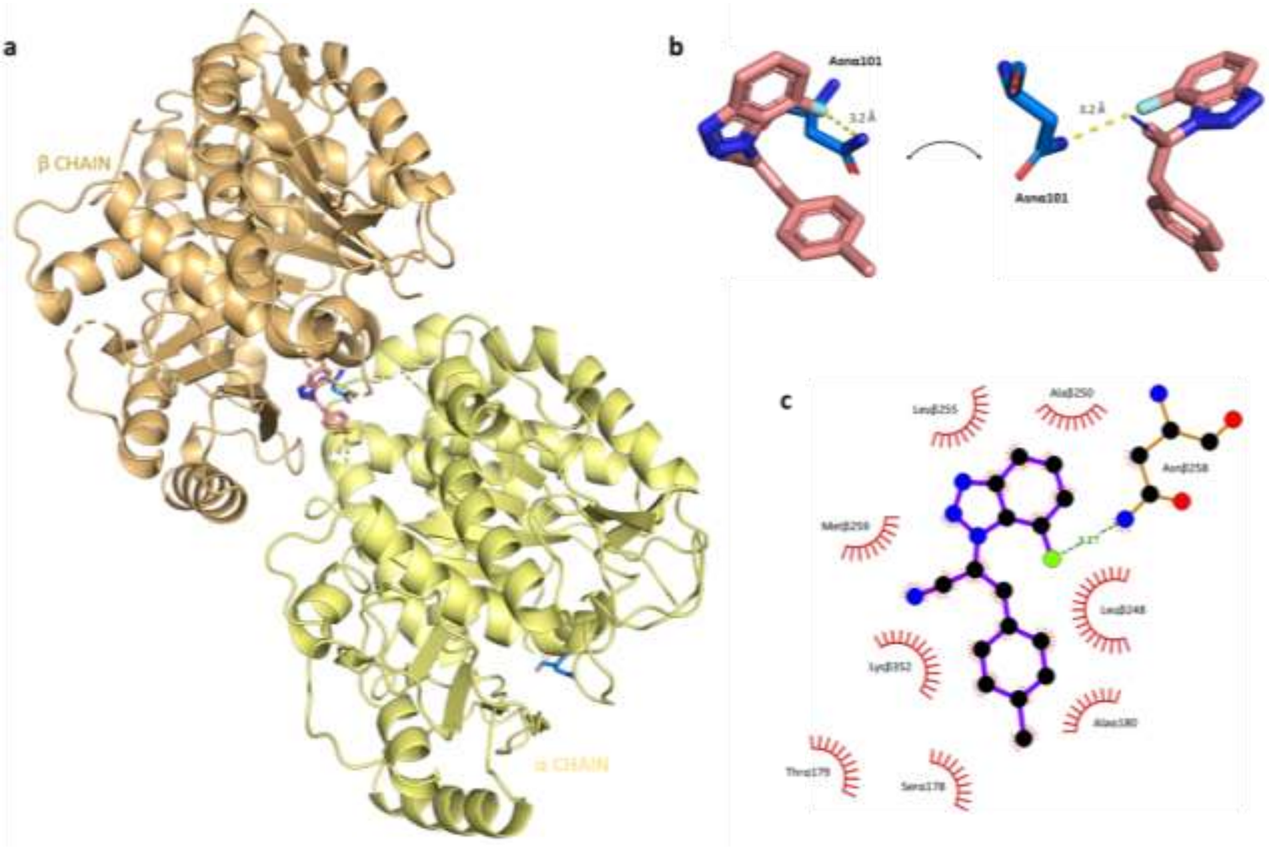


Figure S1. a) Conformational pose of compound **12** (colored in pink), and b) a close-up of the protein and its fluorine bond with Asnα101, also in a reverse side to appreciate it better. Native pdb files were processed via PyMOL. c) 2D-representation of the polar and nonpolar interactions with α− and β−subunits amino acids. Figure obtained through LigPlot$^{+}$. Protein chains and amino acids are reported as in Figure 4. H-bonds are depicted as green dashes as red hash marks.

## ADME predictions (SwissADME)

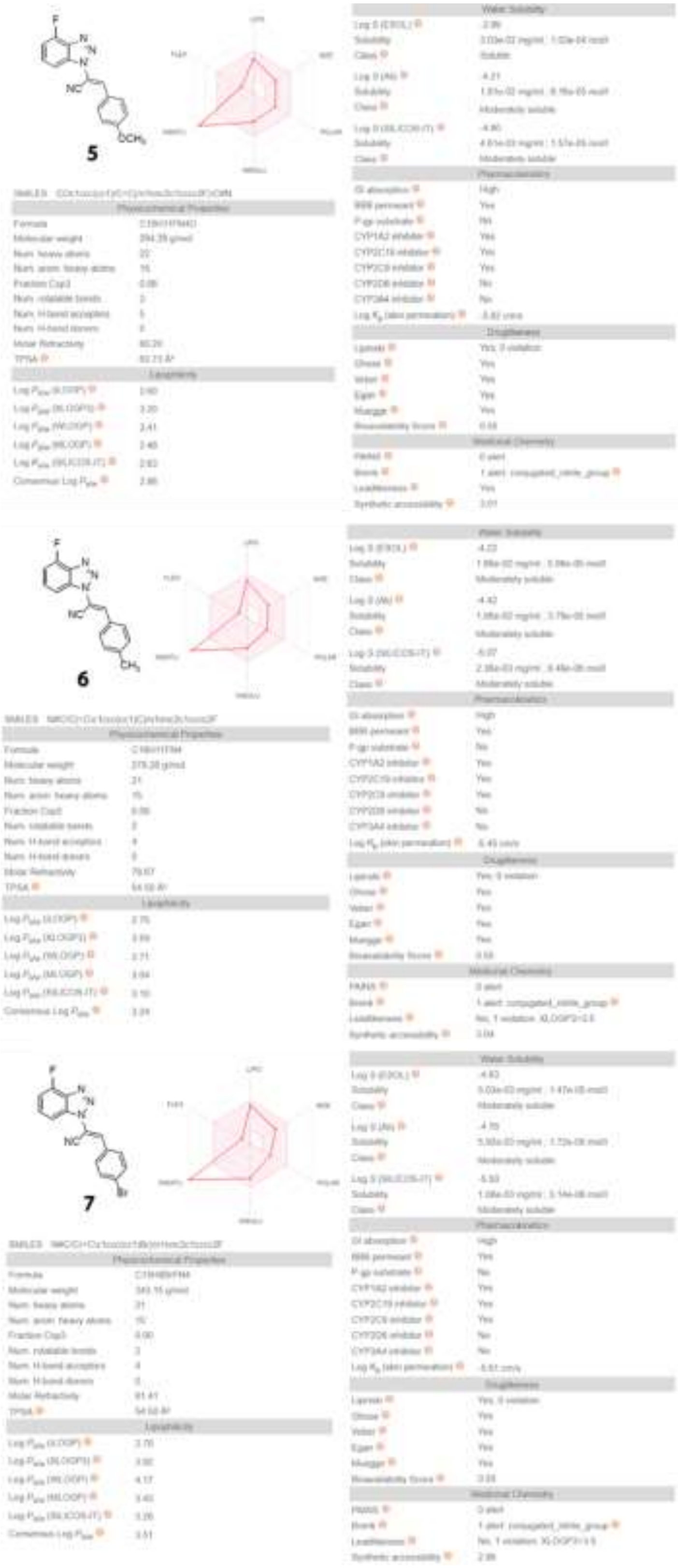


Figure S2. SwissADME prediction for physicochemical, lipophilicity/solubility, pharmacokinetic, drug and leadlikeness properties for compounds **5**-**7**.

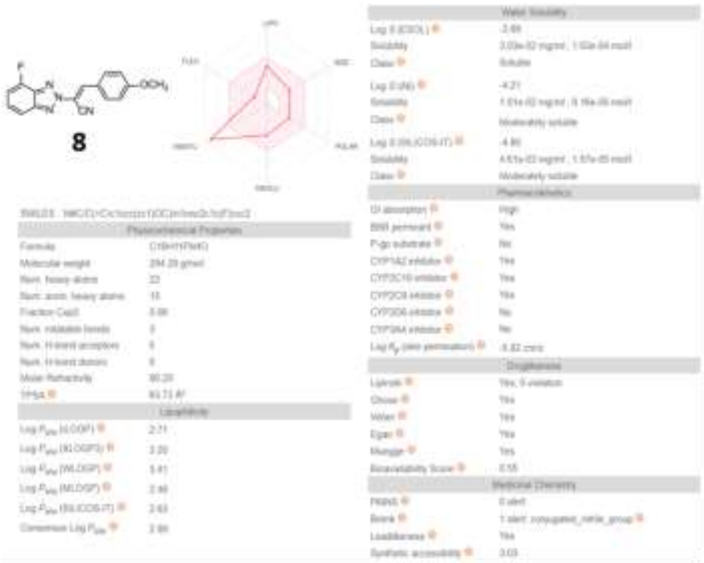


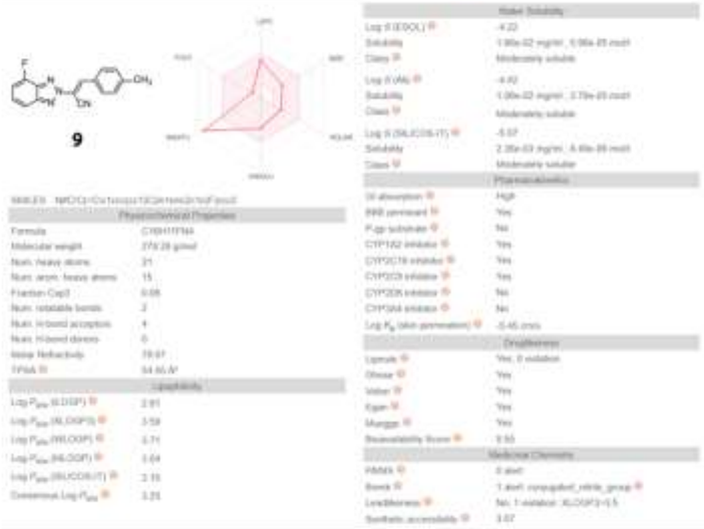


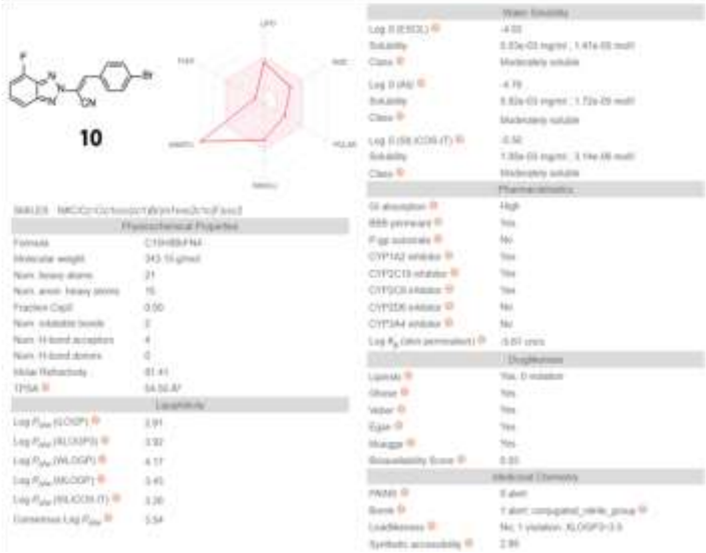


Figure S3. SwissADME prediction for physicochemical, lipophilicity/solubility, pharmacokinetic, drug and leadlikeness properties for compounds **8-10**.

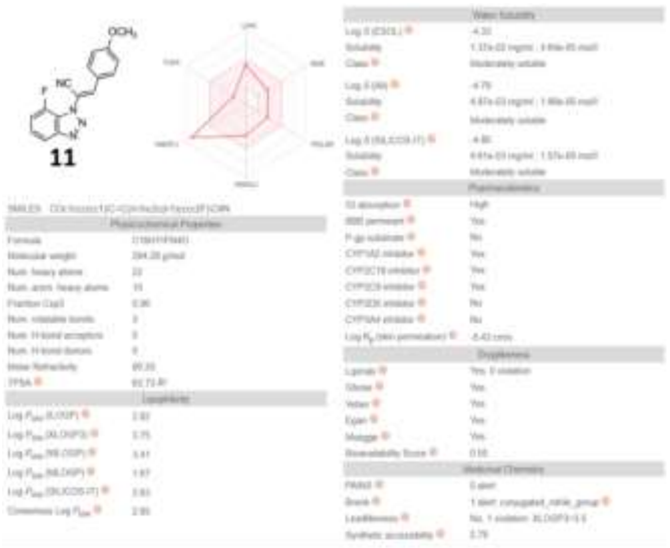


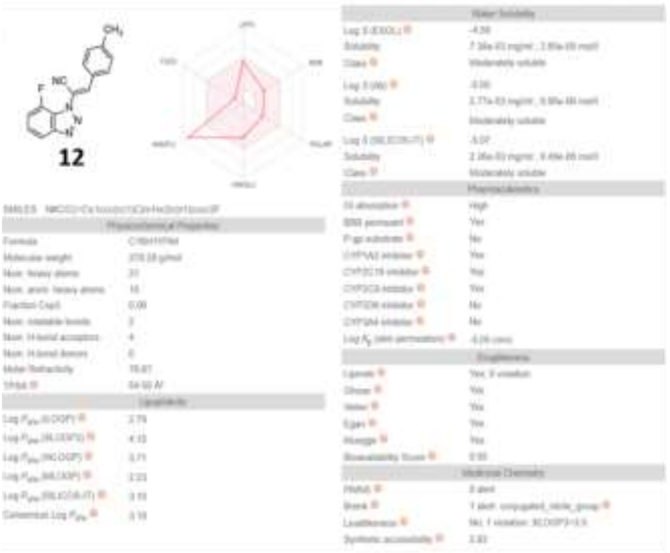


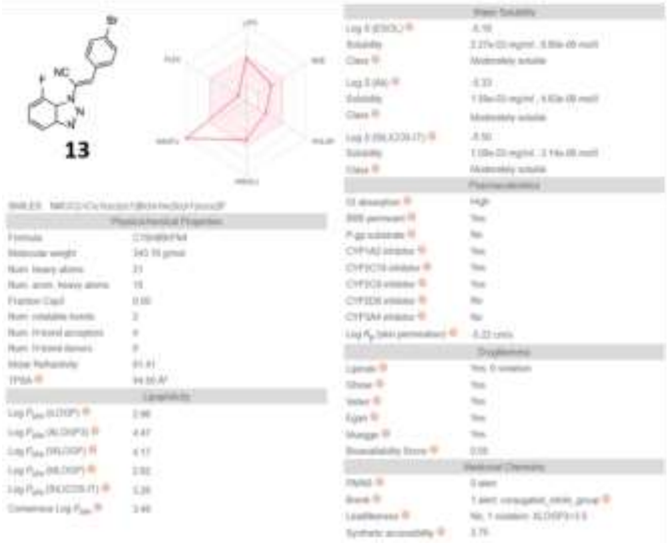


Figure S4. SwissADME prediction for physicochemical, lipophilicity/solubility, pharmacokinetic, drug and leadlikeness properties for compounds **11-13**.

**$^{1}$H and $^{13}$C NMR characterization**

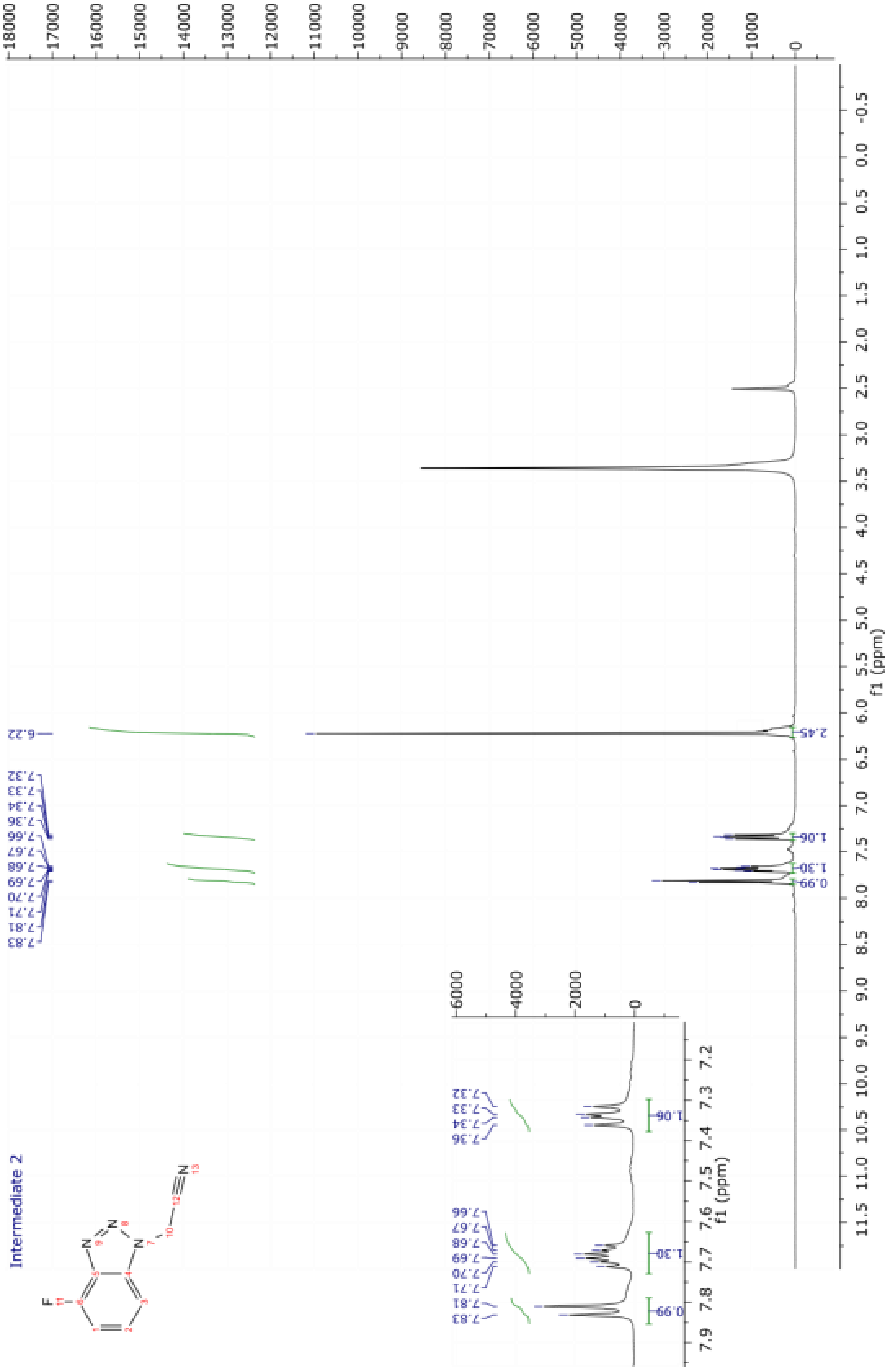


Figure S5. $^{1}$H-NMR spectrum for compound **2**.

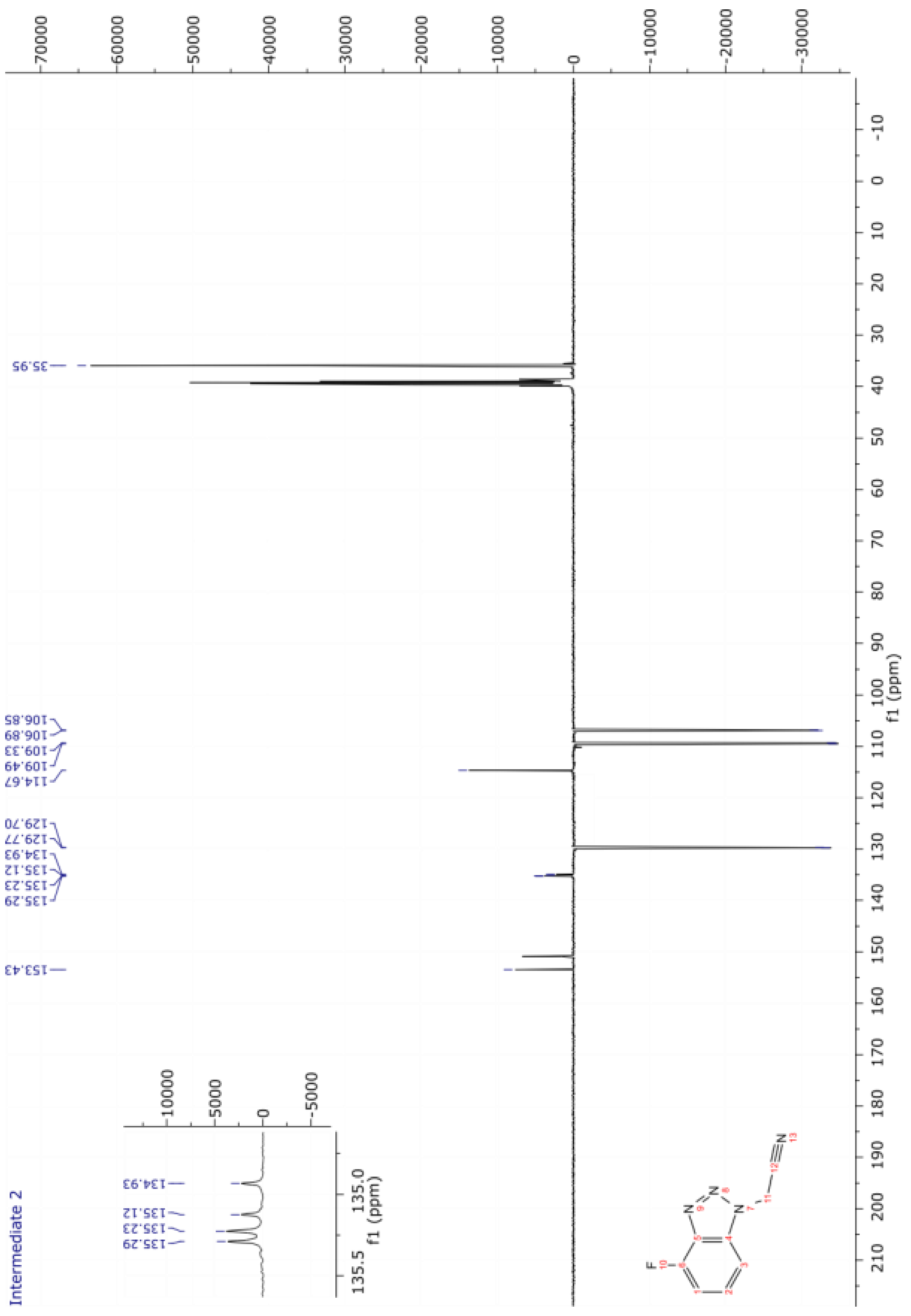


Figure S6. $^{13}$C-NMR spectrum for compound **2**.

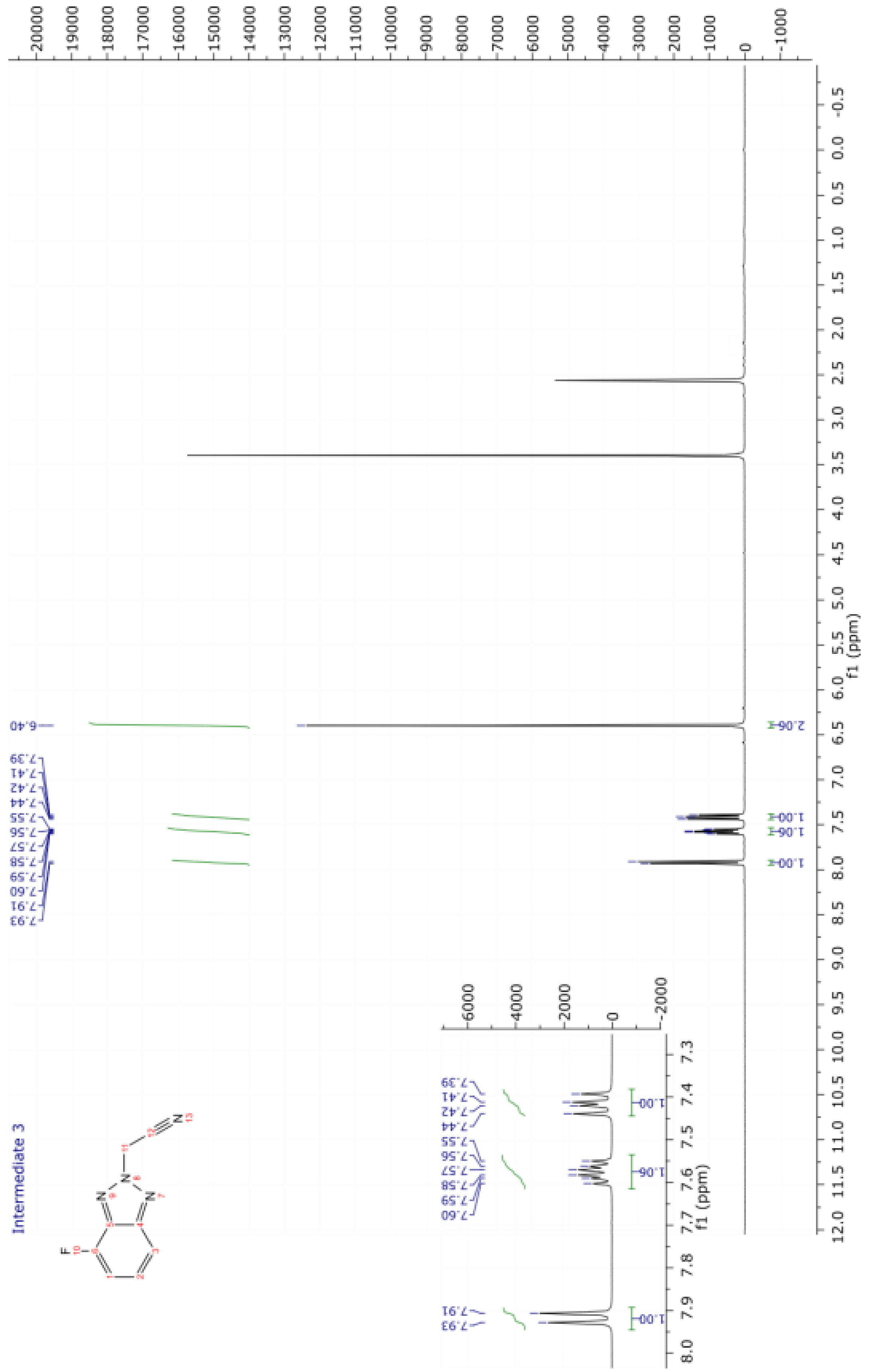


Figure S7. $^{1}$H-NMR spectrum for compound **3**.

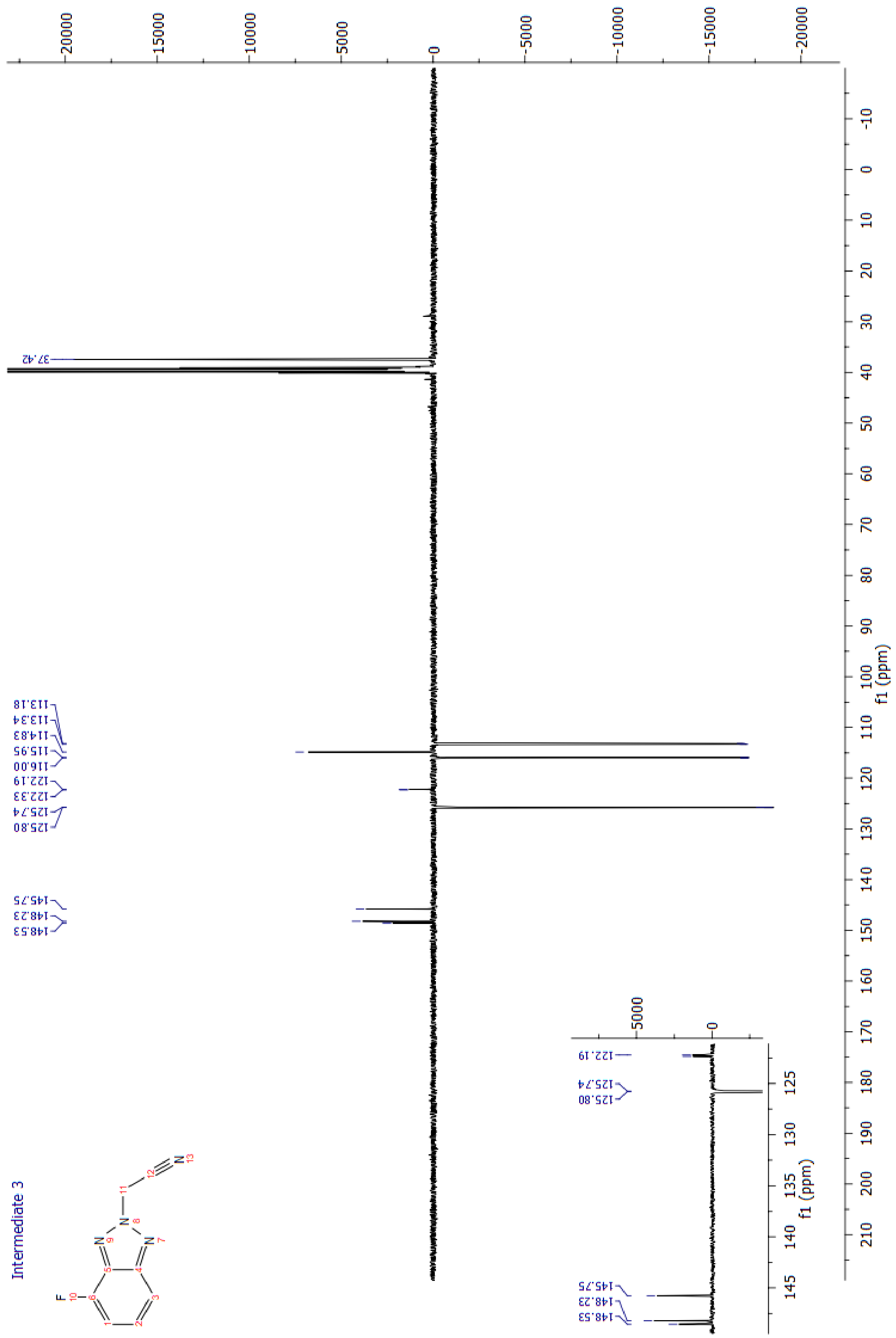


Figure S8. $^{1}$H-NMR spectrum for compound **3**.

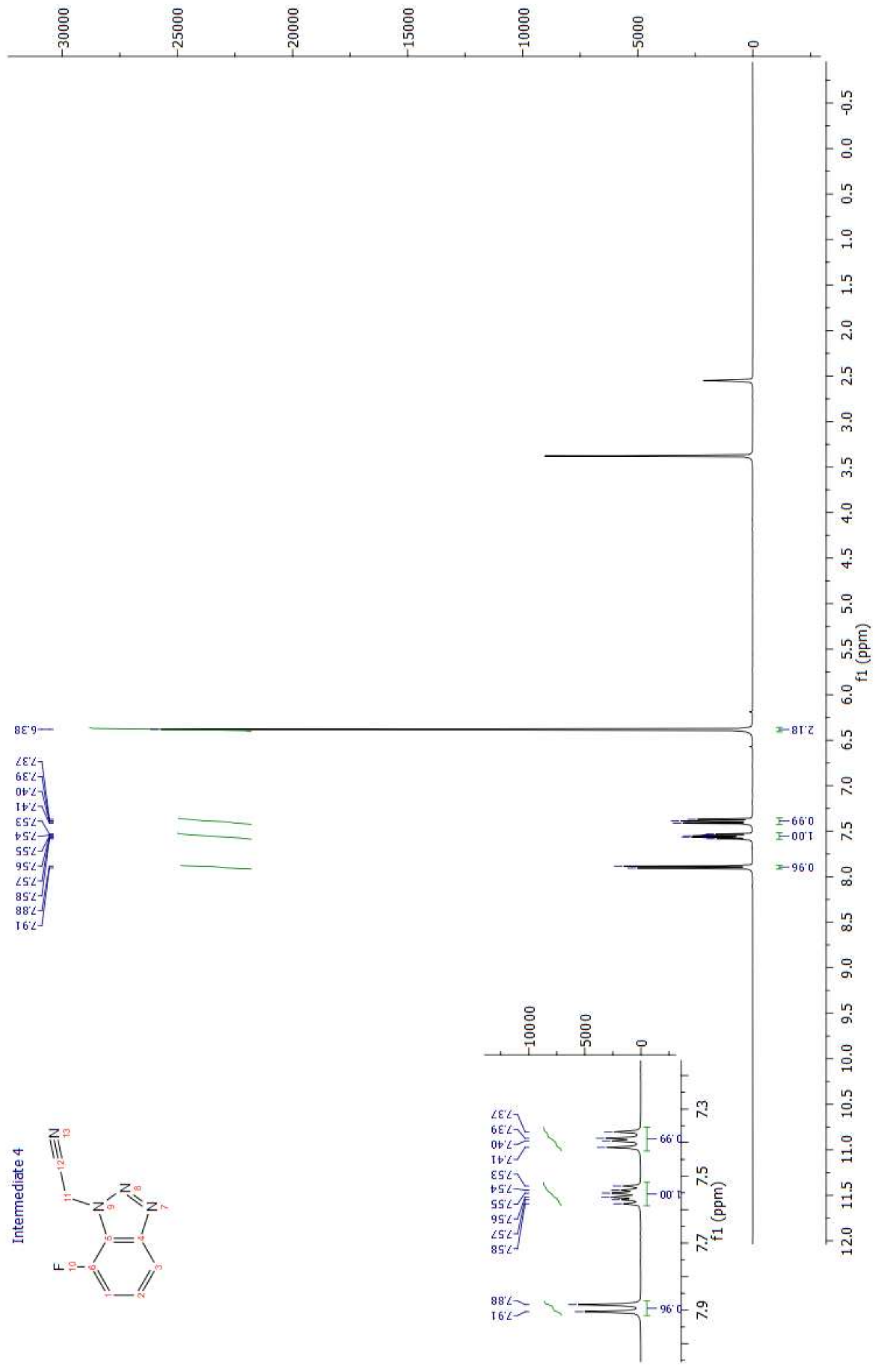


Figure S9. $^{1}$H-NMR spectrum for compound **4**.

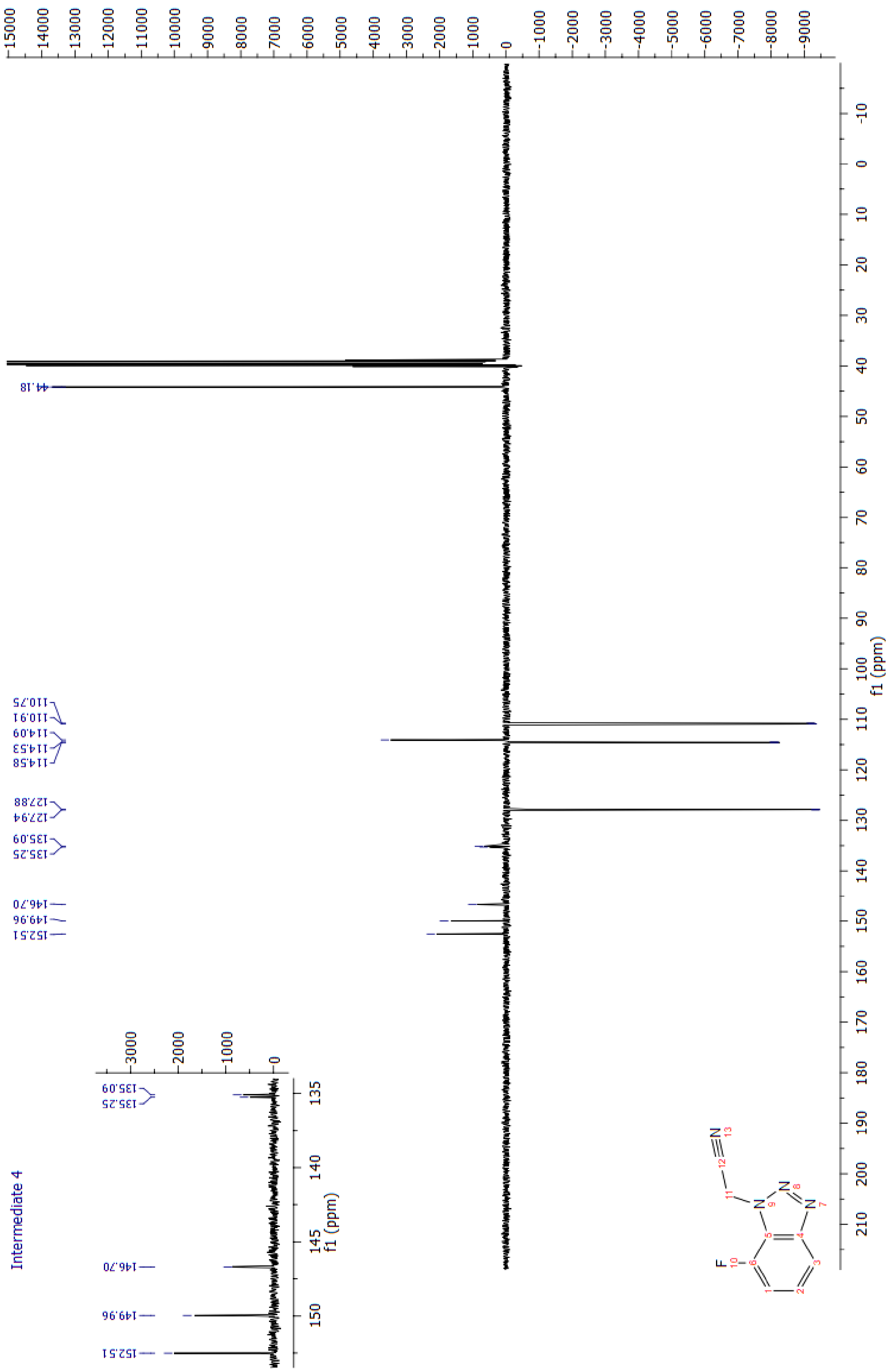


Figure S10. $^{13}$C-NMR spectrum for compound **4**.

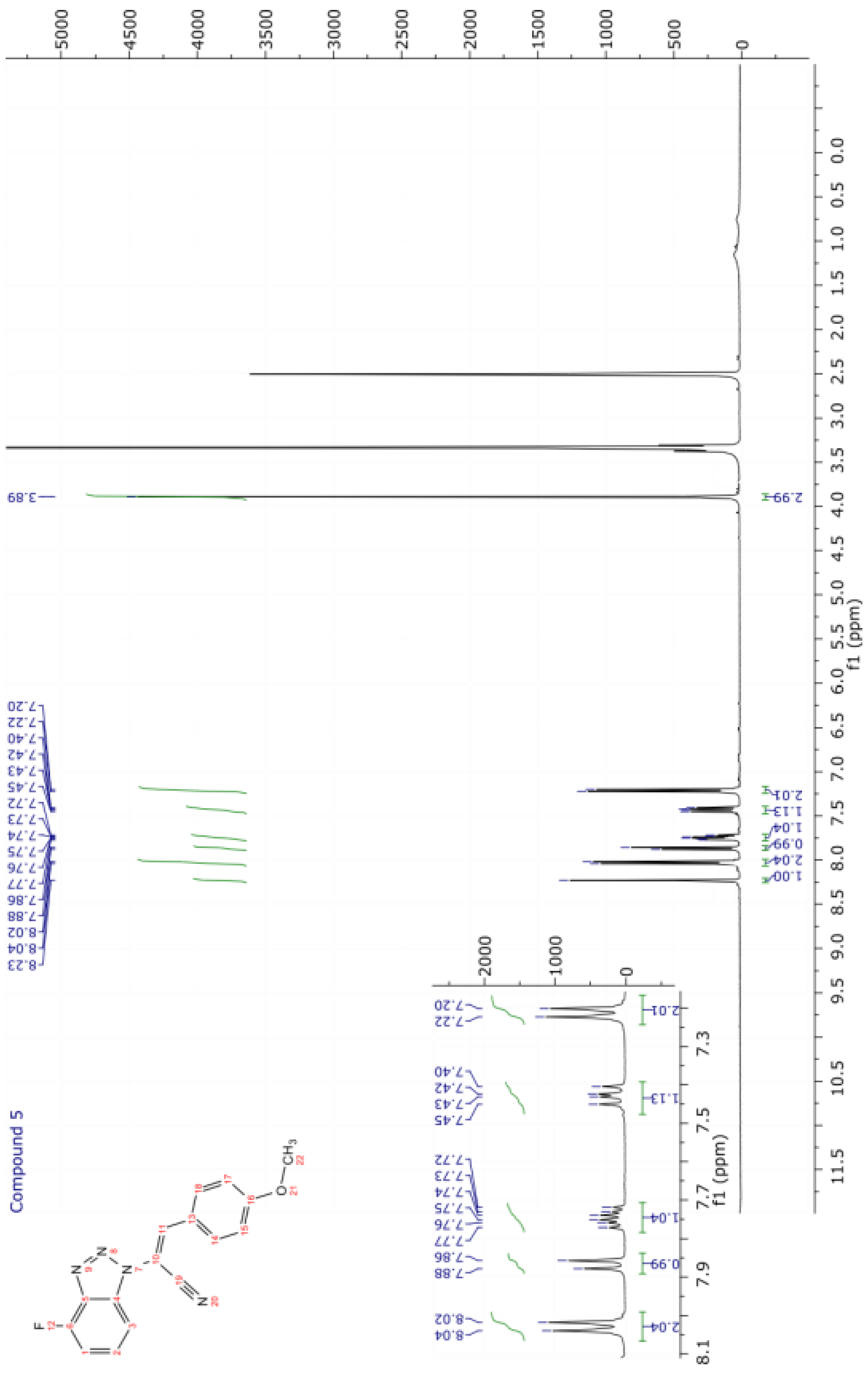


Figure S11. $^{1}$H-NMR spectrum of compound **5**.

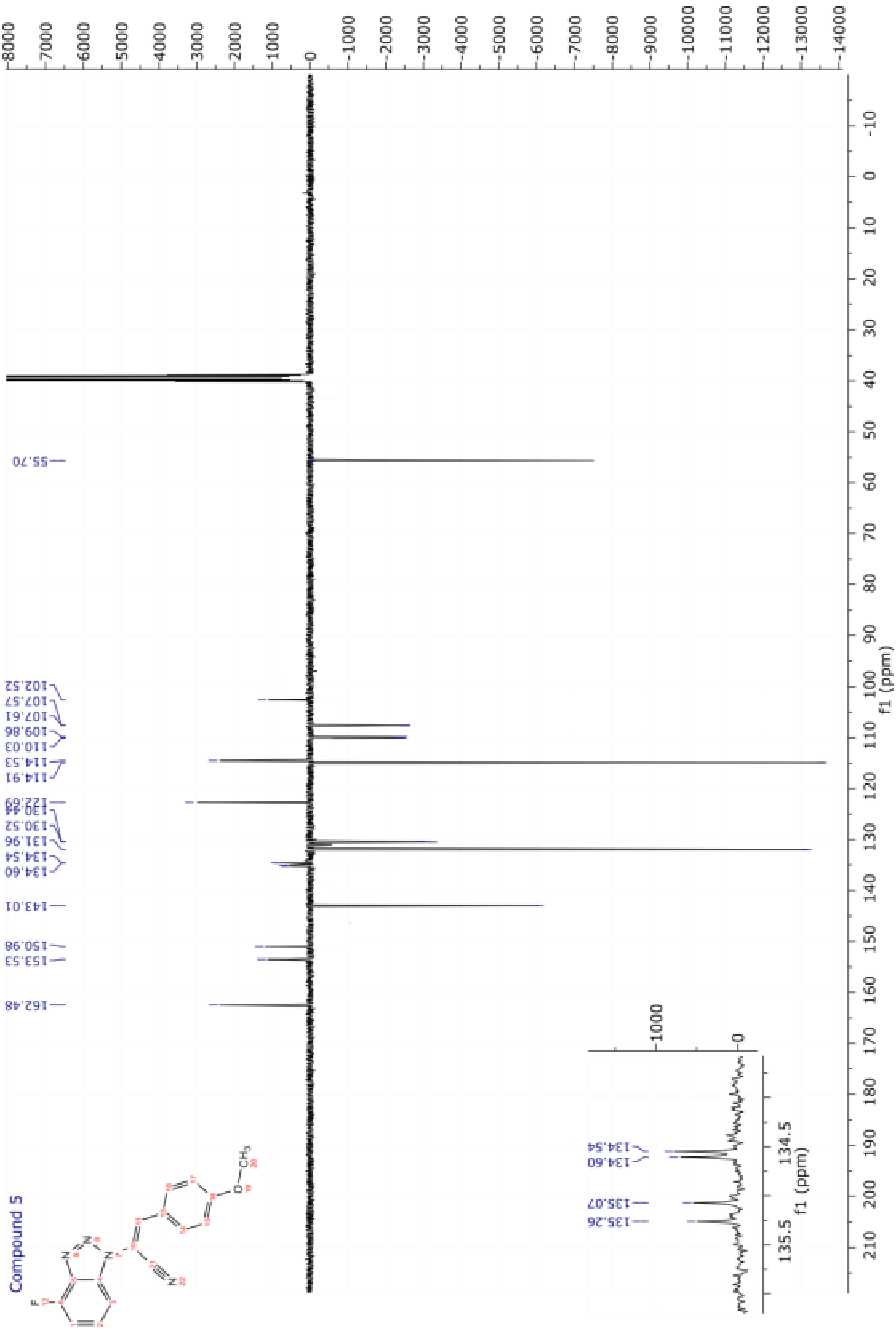


Figure S12. $^{13}$C-NMR spectrum of compound **5**.

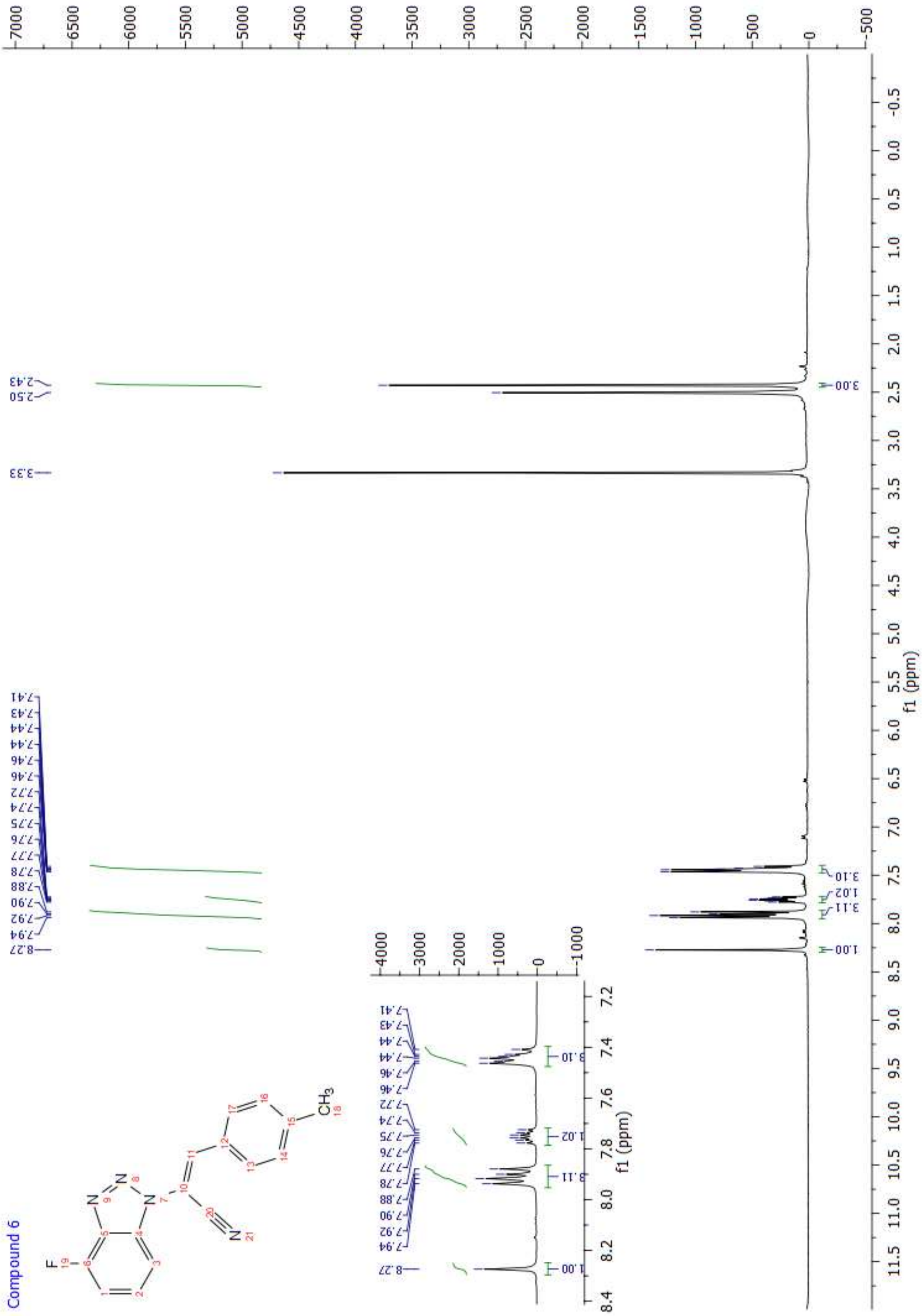


Figure S13. ¹H-NMR spectrum of compound **6**.

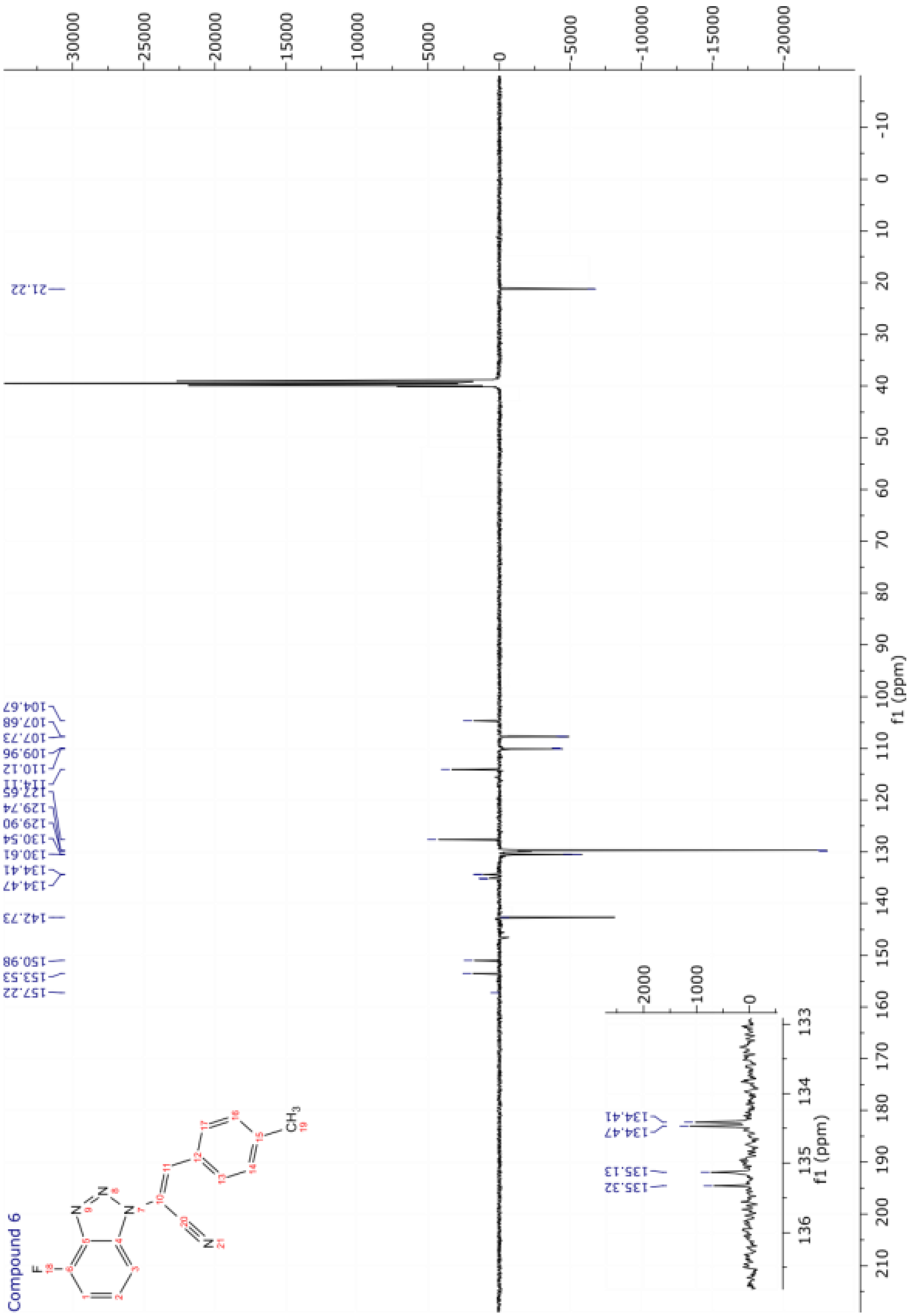


Figure S14. $^{13}$C-NMR spectrum of compound **6**.

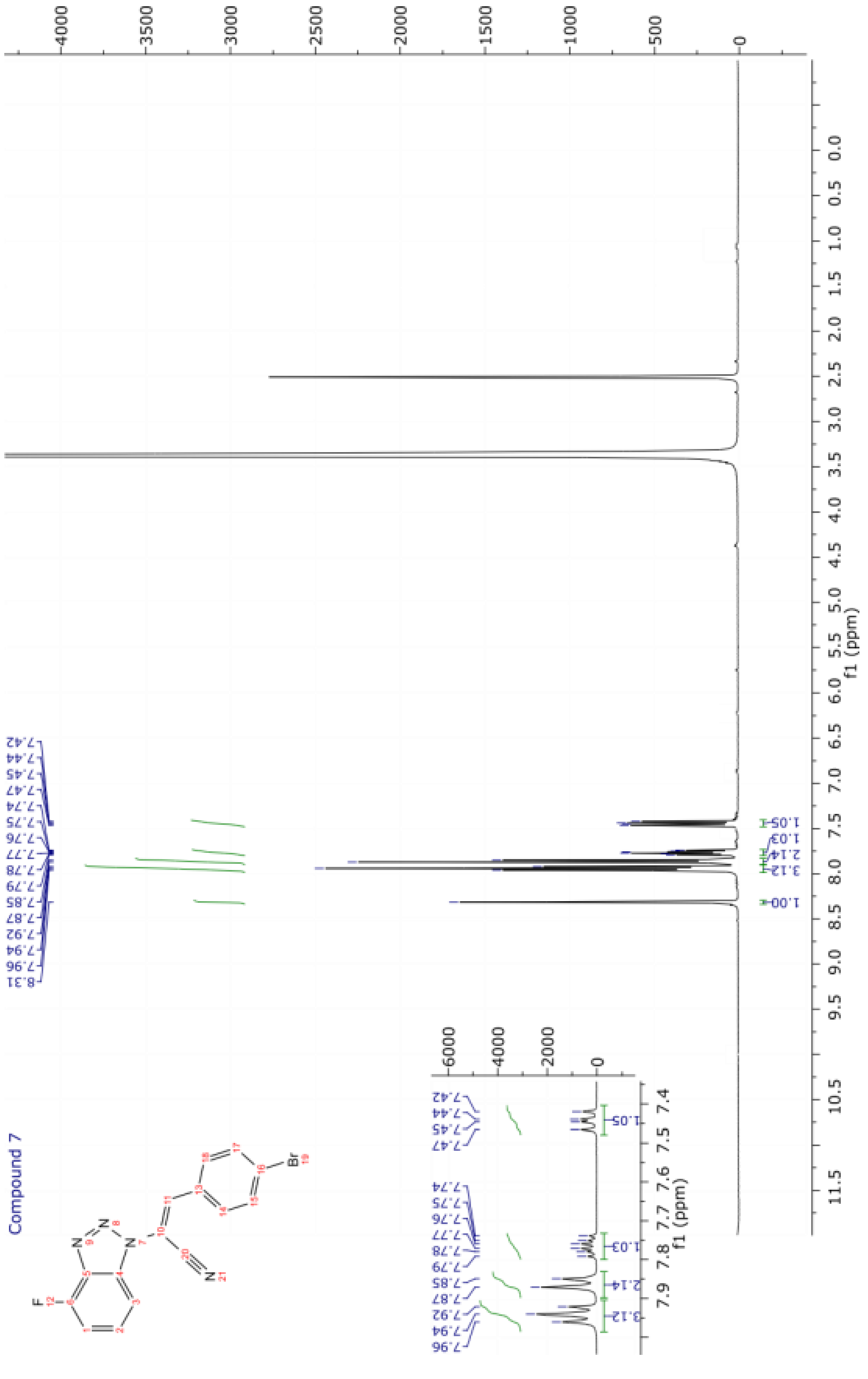


Figure S15. $^{1}$H-NMR spectrum of compound **7**.

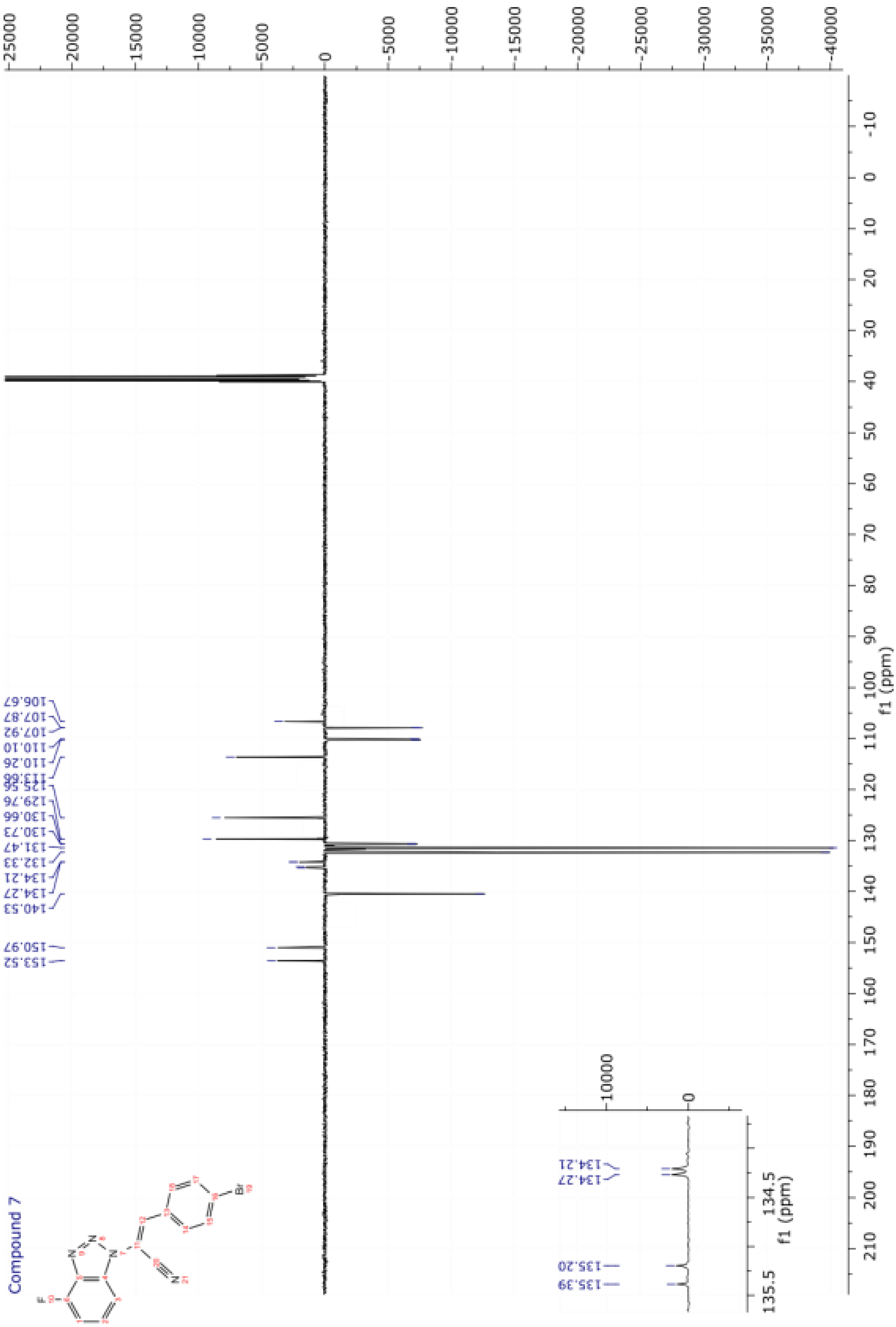


Figure S16. $^{13}$C-NMR spectrum of compound **7**.

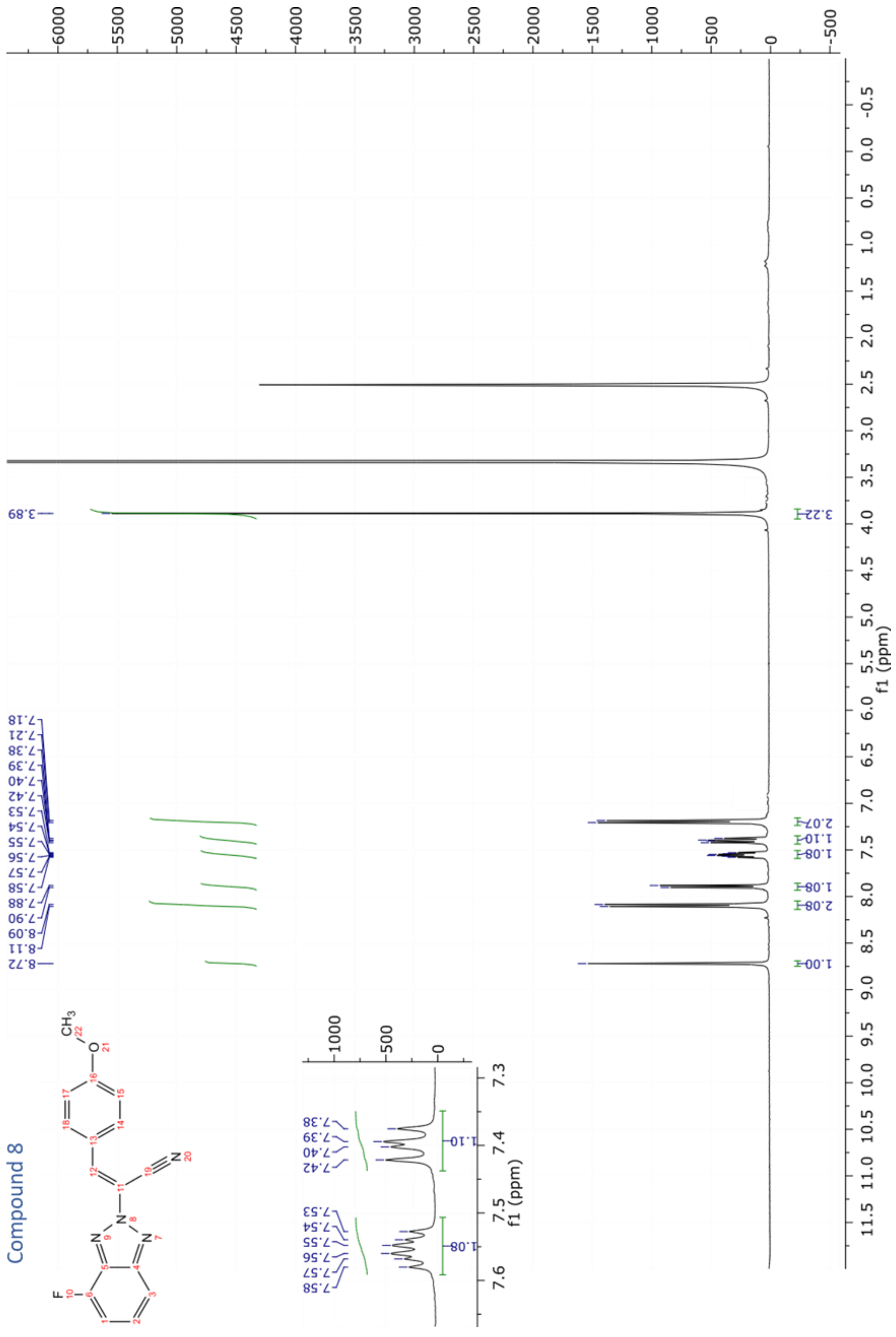


Figure S17. $^{1}$H-NMR spectrum of compound **8**.

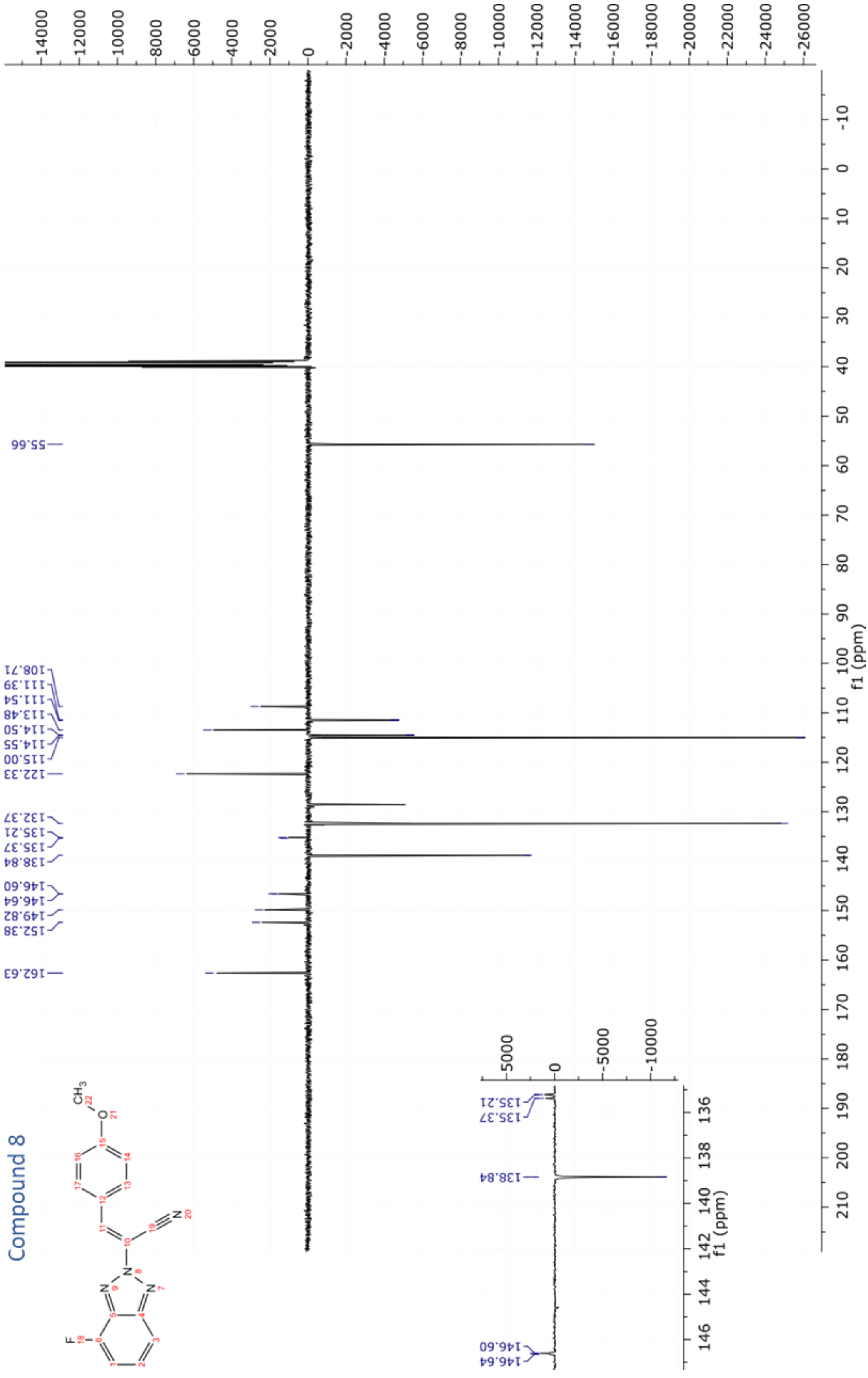


Figure S18. $^{13}$C-NMR spectrum of compound **8**.

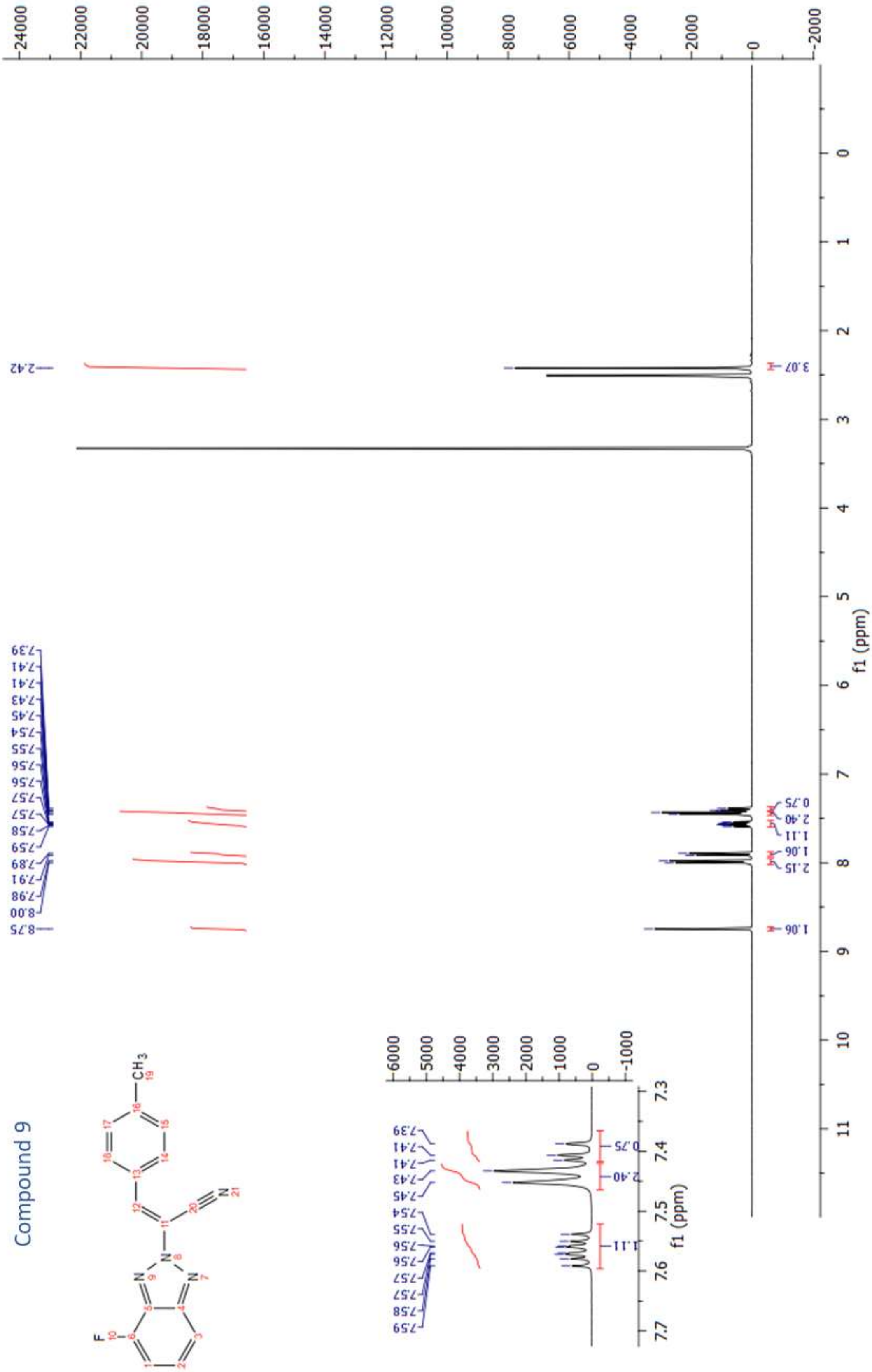


Figure S19. $^{1}$H-NMR spectrum of compound **9**.

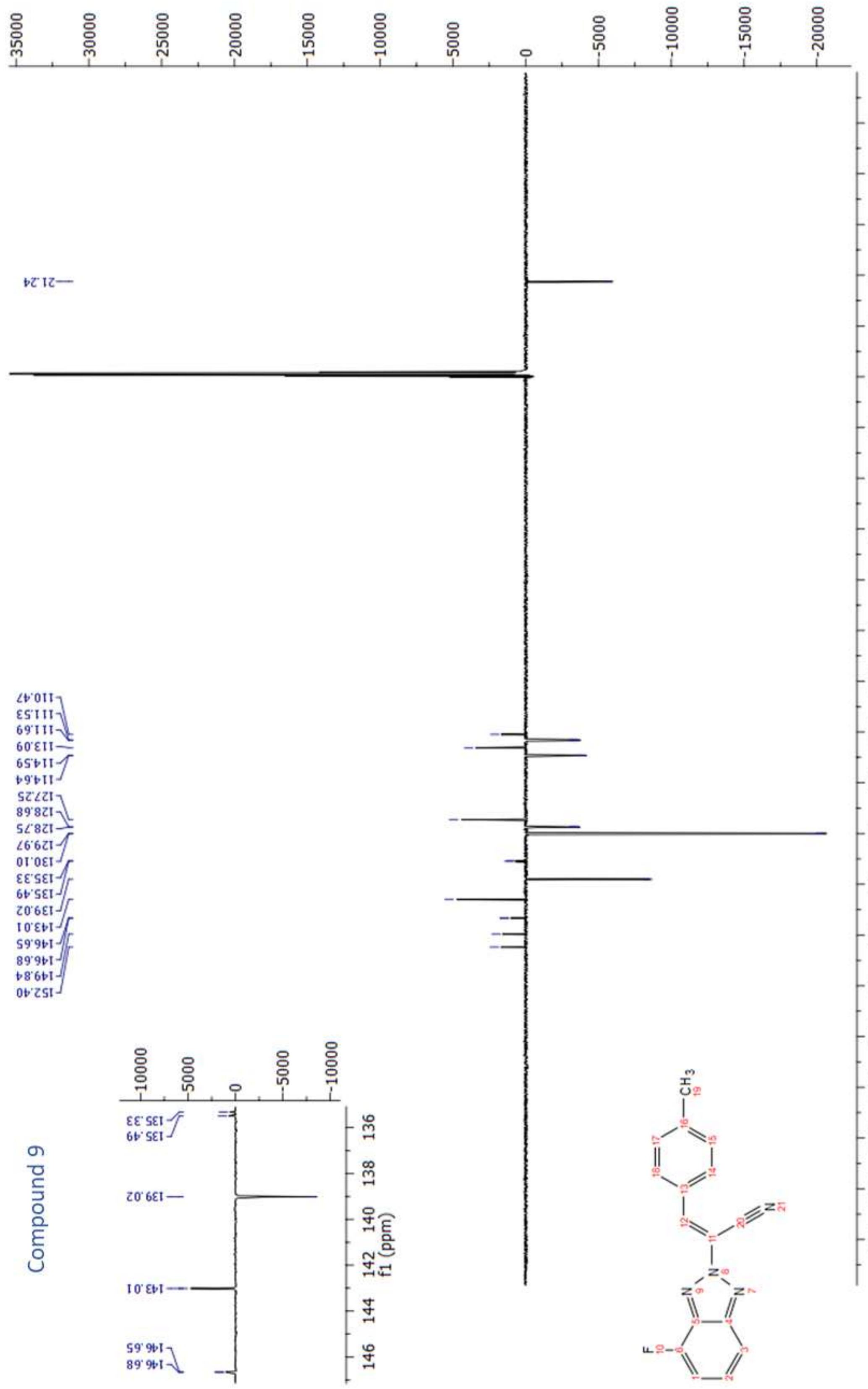


Figure S20. $^{13}C$-NMR spectrum of compound **9**.

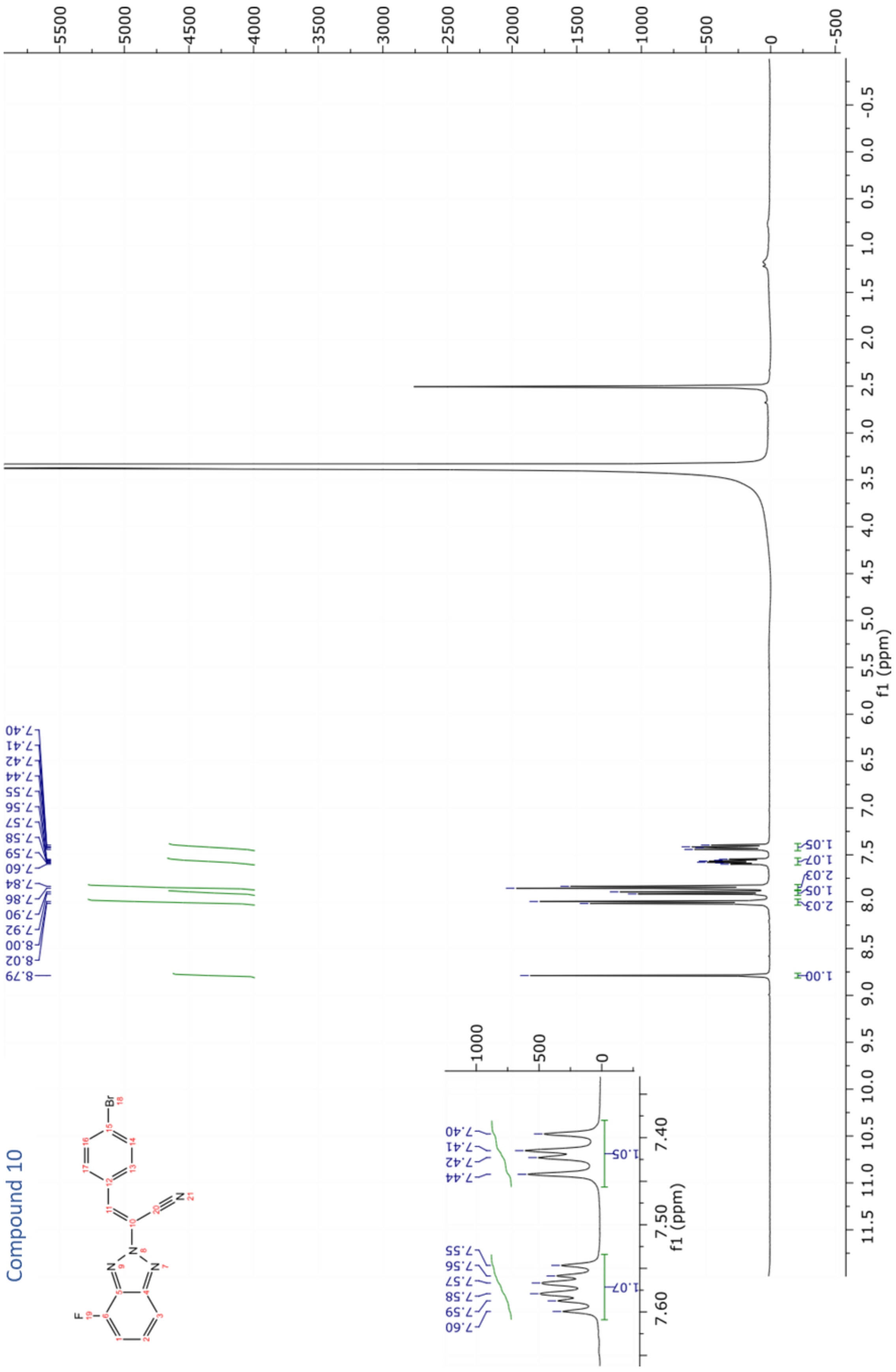


Figure S21. $^{1}$H-NMR spectrum of compound **10**.

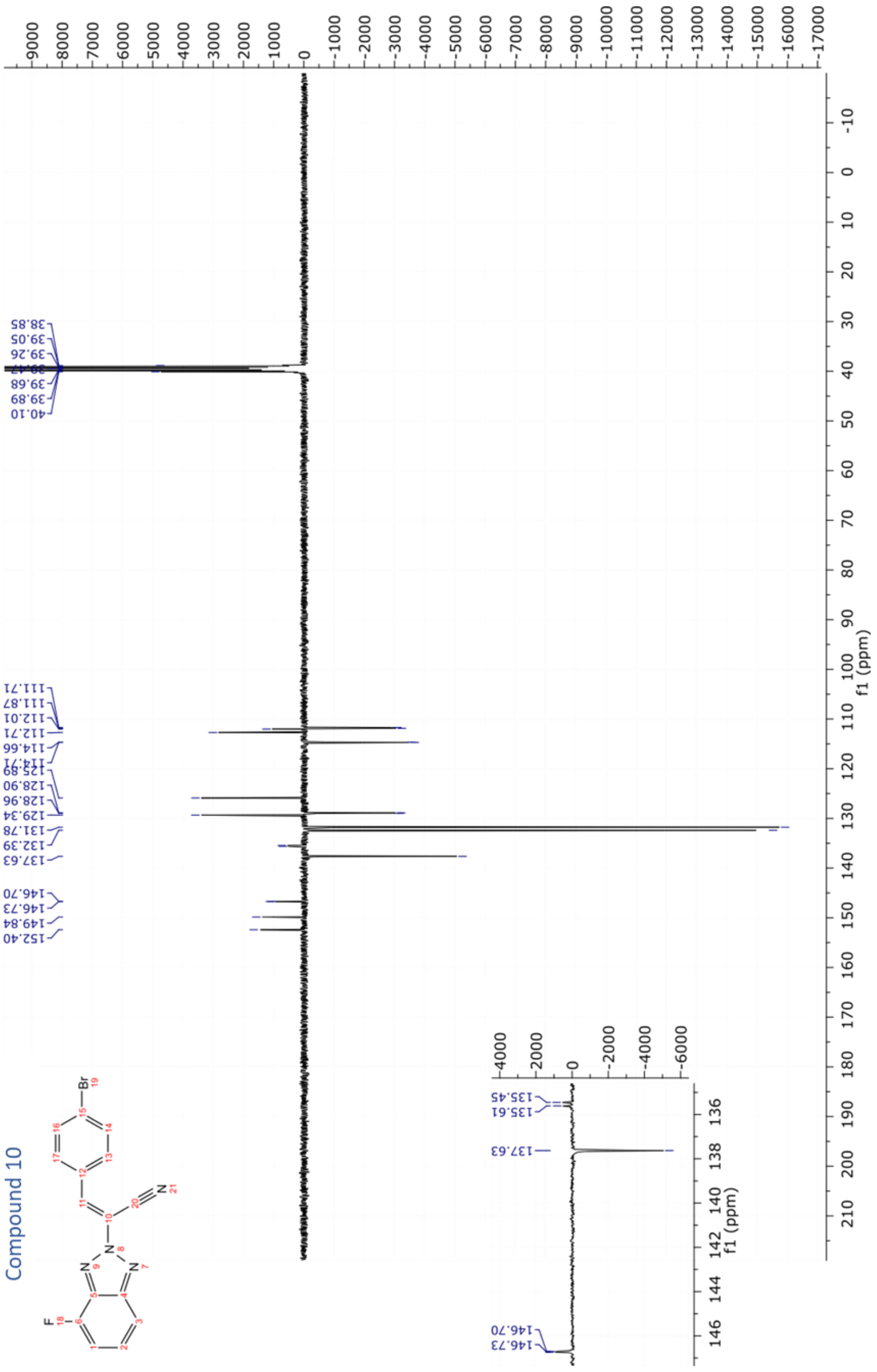


Figure S22. $^{13}$C-NMR spectrum of compound **10**.

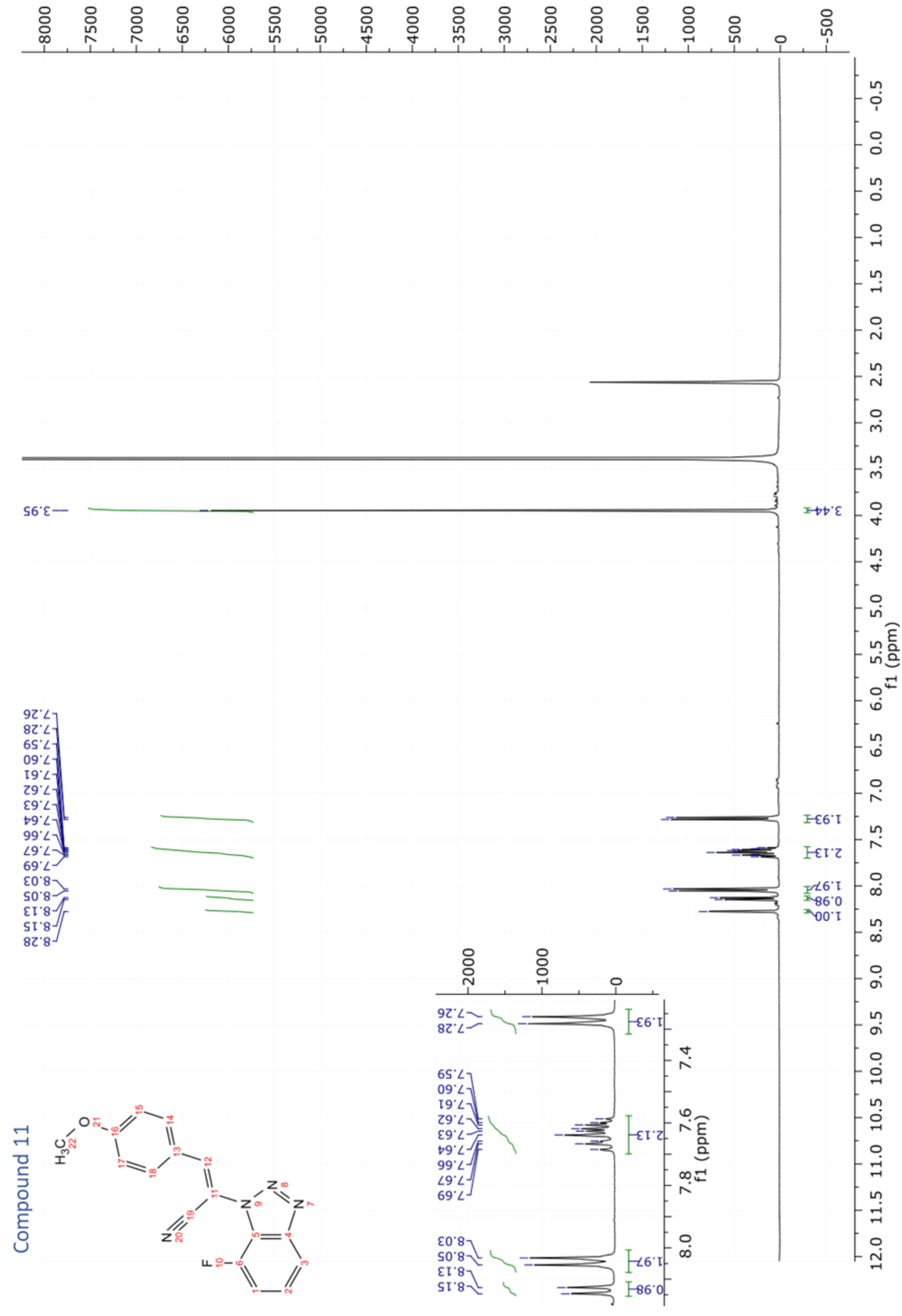


Figure S23. $^{1}$H-NMR spectrum of compound **11**.

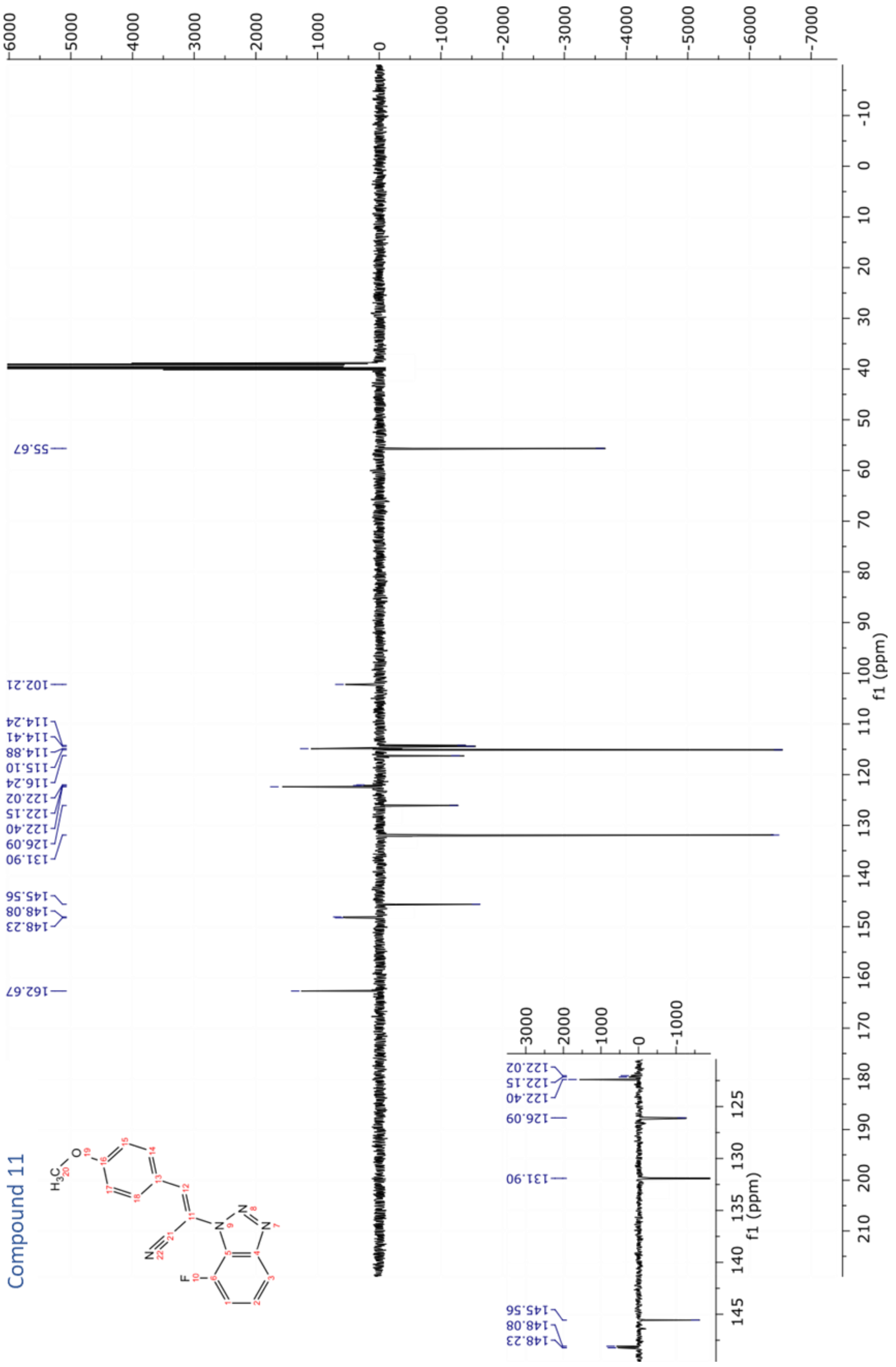


Figure S24. $^{13}$C-NMR spectrum of compound **11**.

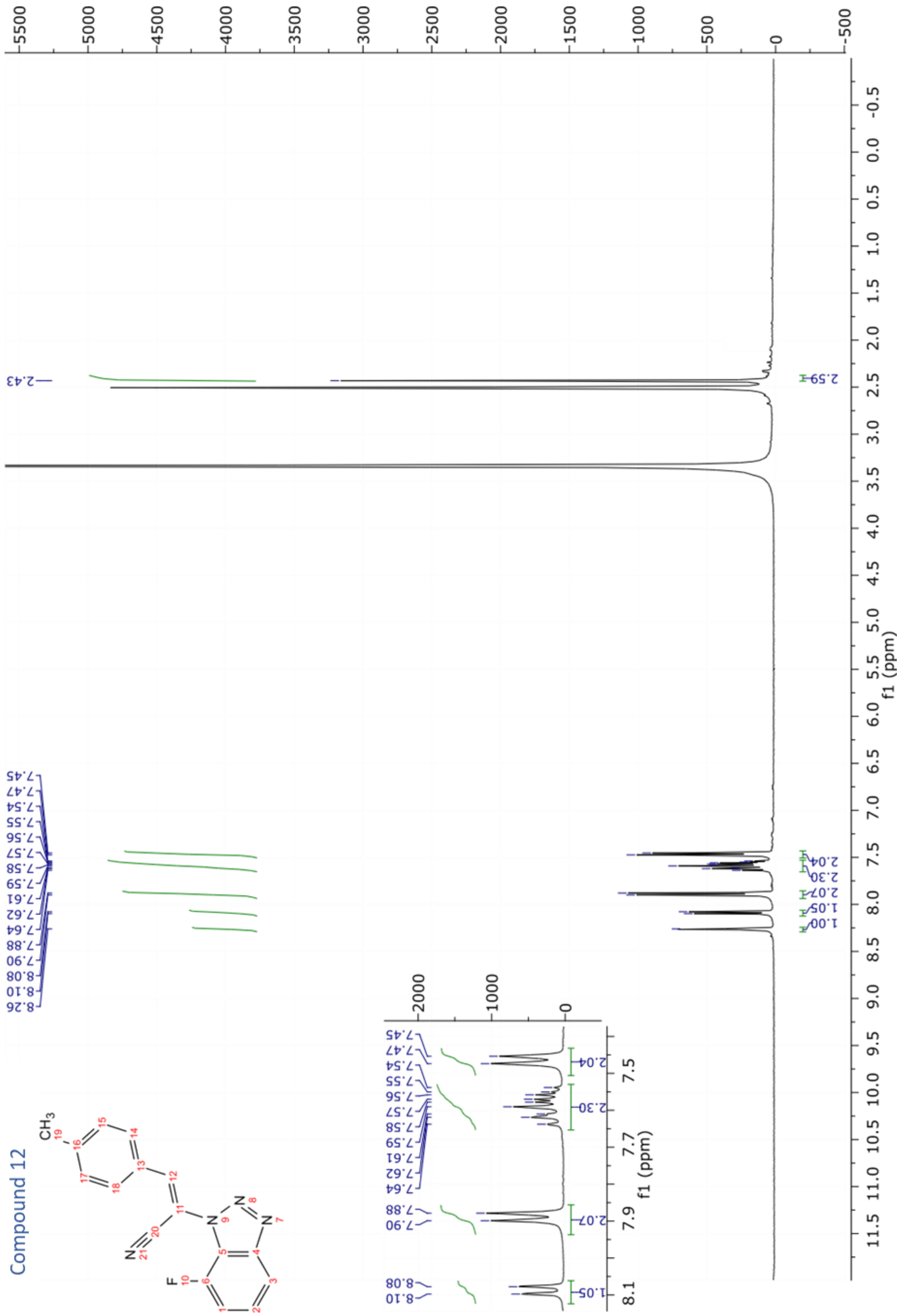


Figure S25. $^{1}$H-NMR spectrum of compound **12**.

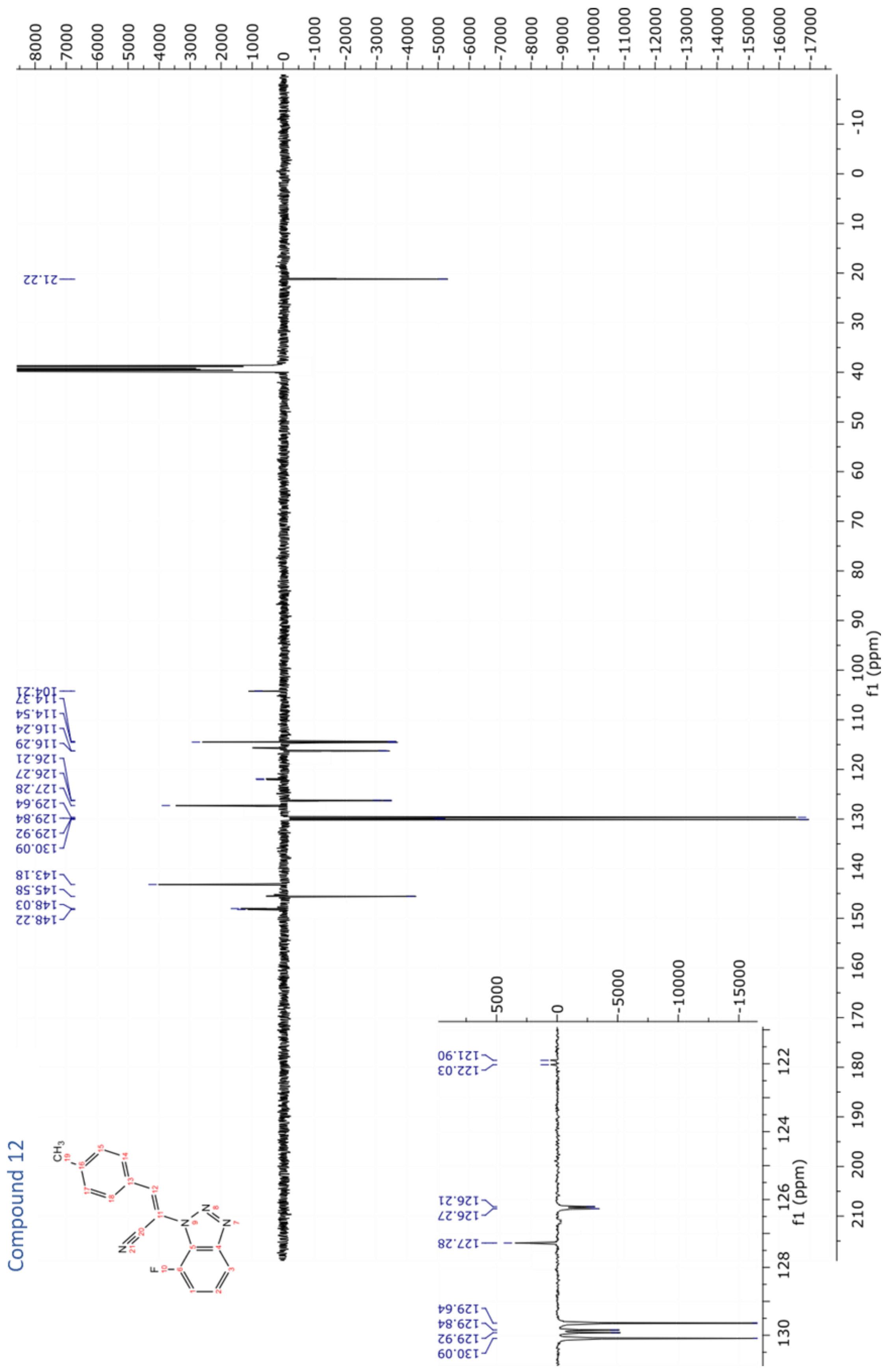


Figure S26. $^{13}$C-NMR spectrum of compound **12**.

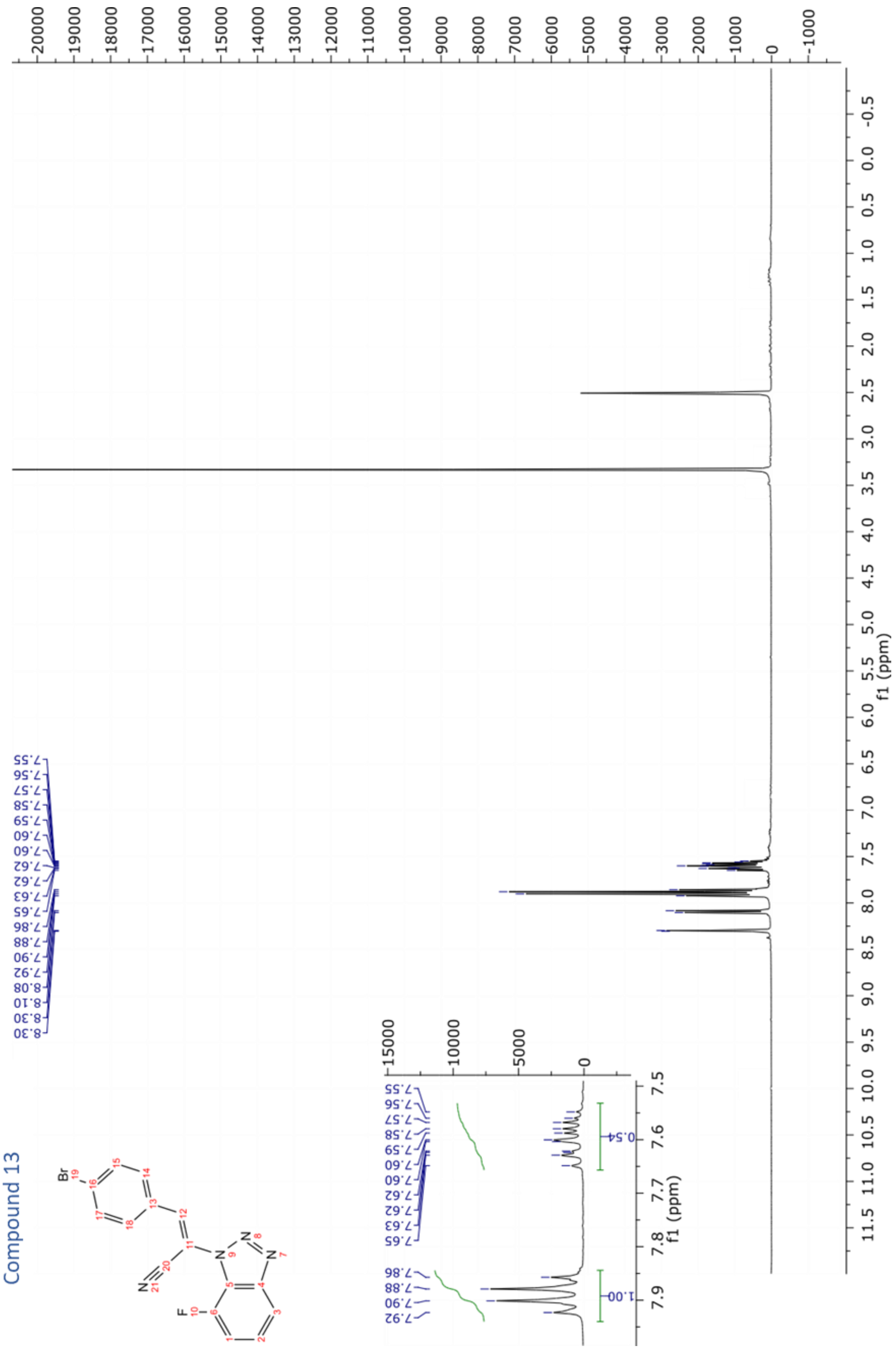


Figure S27. $^{1}$H-NMR spectrum of compound **13**.

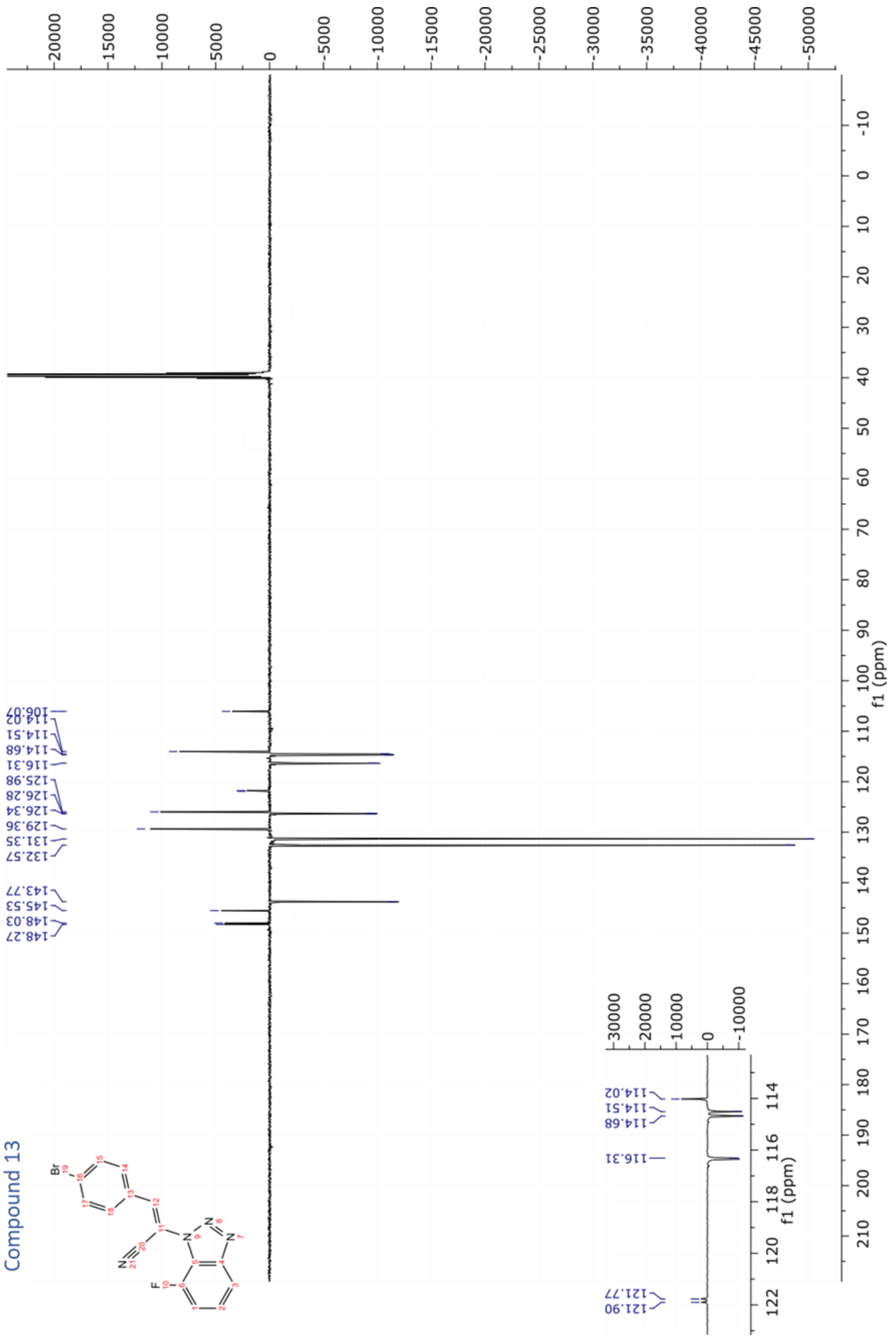


Figure S28. $^{13}$C-NMR spectrum of compound **13**.

**NCI60 *in vitro* screening**

Table S1. NCS numbers for compounds **5-13** (NCI60 screening).

| Label | NSC code |
|---|---|
| **5** | D - 822612 / 1 |
| **6** | D - 833304 / 1 |
| **7** | D - 816687 / 1 |
| **8** | D - 813352 / 1 |
| **9** | D - 813100 / 1 |
| **10** | D - 813355 / 1 |
| **11** | D - 813098 / 1 |
| **12** | D - 813353 / 1 |
| **13** | D - 813354 / 1 |

# National Cancer Institute Developmental Therapeutics Program
## In-Vitro Testing Results

| | | | |
|---|---|---|---|
| NSC : D - 822612 / 1 | Experiment ID : 2010RS45 | Test Type : 08 | Units : Molar |
| Report Date : November 14, 2020 | Test Date : October 19, 2020 | QNS : | MC : |
| COMI : FR15 | Stain Reagent : SRB Dual-Pass Related | SSPL : 1C0V | |

| | | | Log10 Concentration | | | | | | | | | | | | | |
|---|---|---|---|---|---|---|---|---|---|---|---|---|---|---|---|---|
| | Time | | Mean Optical Densities | | | | | Percent Growth | | | | | | | | |
| Panel/Cell Line | Zero | Ctrl | -8.0 | -7.0 | -6.0 | -5.0 | -4.0 | -8.0 | -7.0 | -6.0 | -5.0 | -4.0 | GI50 | | TGI | LC50 |
| Leukemia | | | | | | | | | | | | | | | | |
| CCRF-CEM | 0.402 | 2.205 | 2.053 | 1.787 | 0.736 | 0.669 | 0.668 | 92 | 77 | 18 | 15 | 15 | 2.88E-7 | | > 1.00E-4 | > 1.00E-4 |
| HL-60(TB) | 0.647 | 3.071 | 2.965 | 2.648 | 0.597 | 0.536 | 0.475 | 96 | 83 | -8 | -17 | -27 | 2.29E-7 | | 8.20E-7 | > 1.00E-4 |
| K-562 | 0.151 | 1.789 | 1.656 | 0.599 | 0.272 | 0.249 | 0.310 | 92 | 27 | 7 | 6 | 10 | 4.45E-8 | | > 1.00E-4 | > 1.00E-4 |
| MOLT-4 | 0.450 | 2.721 | 2.621 | 2.615 | 0.987 | 0.761 | 0.730 | 96 | 95 | 24 | 14 | 12 | 4.29E-7 | | > 1.00E-4 | > 1.00E-4 |
| RPMI-8226 | 0.514 | 1.973 | 2.018 | 1.990 | 0.682 | 0.648 | 0.771 | 103 | 101 | 12 | 9 | 18 | 3.72E-7 | | > 1.00E-4 | > 1.00E-4 |
| SR | 0.264 | 0.823 | 0.760 | 0.561 | 0.385 | 0.368 | 0.275 | 89 | 53 | 22 | 19 | 2 | 1.26E-7 | | > 1.00E-4 | > 1.00E-4 |
| Non-Small Cell Lung Cancer | | | | | | | | | | | | | | | | |
| A549/ATCC | 0.451 | 2.288 | 2.210 | 2.131 | 1.247 | 0.701 | 0.596 | 96 | 91 | 43 | 14 | 8 | 7.26E-7 | | > 1.00E-4 | > 1.00E-4 |
| EKVX | 0.641 | 1.861 | 1.797 | 1.745 | 1.085 | 0.866 | 0.793 | 95 | 90 | 36 | 18 | 12 | 5.60E-7 | | > 1.00E-4 | > 1.00E-4 |
| HOP-62 | 0.482 | 1.403 | 1.404 | 1.302 | 0.821 | 0.646 | 0.466 | 100 | 89 | 37 | 18 | -3 | 5.58E-7 | | 6.90E-5 | > 1.00E-4 |
| HOP-92 | 1.320 | 1.756 | 1.650 | 1.540 | 1.421 | 1.349 | 1.228 | 76 | 50 | 23 | 7 | -7 | 1.03E-7 | | 3.04E-5 | > 1.00E-4 |
| NCI-H226 | 0.632 | 1.600 | 1.504 | 1.457 | 1.159 | 1.021 | 1.006 | 90 | 85 | 54 | 40 | 39 | 2.05E-6 | | > 1.00E-4 | > 1.00E-4 |
| NCI-H23 | 0.783 | 2.632 | 2.569 | 2.421 | 1.391 | 0.915 | 0.576 | 97 | 89 | 33 | 7 | -26 | 4.92E-7 | | 1.63E-5 | > 1.00E-4 |
| NCI-H322M | 0.915 | 2.309 | 2.207 | 2.151 | 1.490 | 1.133 | 1.043 | 93 | 89 | 41 | 16 | 9 | 6.53E-7 | | > 1.00E-4 | > 1.00E-4 |
| NCI-H460 | 0.235 | 2.301 | 2.374 | 2.409 | 0.480 | 0.298 | 0.261 | 104 | 105 | 12 | 3 | 1 | 3.90E-7 | | > 1.00E-4 | > 1.00E-4 |
| NCI-H522 | 1.057 | 2.523 | 2.495 | 1.942 | 1.119 | 0.752 | 0.598 | 98 | 60 | 4 | -29 | -43 | 1.53E-7 | | 1.34E-6 | > 1.00E-4 |
| Colon Cancer | | | | | | | | | | | | | | | | |
| COLO 205 | 0.590 | 2.175 | 2.197 | 2.057 | 0.839 | 0.639 | 0.593 | 101 | 93 | 16 | 3 | | 3.58E-7 | | > 1.00E-4 | > 1.00E-4 |
| HCC-2998 | 0.473 | 1.916 | 1.789 | 1.755 | 1.184 | 0.496 | 0.520 | 91 | 89 | 49 | 2 | 3 | 9.60E-7 | | > 1.00E-4 | > 1.00E-4 |
| HCT-116 | 0.333 | 2.973 | 2.962 | 2.605 | 0.986 | 0.542 | 0.449 | 100 | 86 | 25 | 8 | 4 | 3.87E-7 | | > 1.00E-4 | > 1.00E-4 |
| HCT-15 | 0.342 | 2.366 | 2.279 | 1.365 | 0.832 | 0.529 | 0.551 | 96 | 51 | 24 | 9 | 10 | 1.05E-7 | | > 1.00E-4 | > 1.00E-4 |
| HT29 | 0.583 | 2.717 | 2.757 | 2.328 | 0.655 | 0.549 | 0.548 | 102 | 82 | 3 | -6 | -6 | 2.54E-7 | | 2.31E-6 | > 1.00E-4 |
| KM12 | 0.552 | 2.773 | 2.641 | 2.456 | 0.957 | 0.778 | 0.689 | 94 | 86 | 18 | 10 | 6 | 3.38E-7 | | > 1.00E-4 | > 1.00E-4 |
| SW-620 | 0.278 | 2.112 | 1.961 | 1.279 | 0.629 | 0.530 | 0.448 | 92 | 55 | 19 | 14 | 9 | 1.35E-7 | | > 1.00E-4 | > 1.00E-4 |
| CNS Cancer | | | | | | | | | | | | | | | | |
| SF-268 | 1.070 | 2.596 | 2.573 | 2.512 | 1.926 | 1.615 | 1.634 | 99 | 95 | 56 | 36 | 37 | 1.99E-6 | | > 1.00E-4 | > 1.00E-4 |
| SF-295 | 0.453 | 2.186 | 2.129 | 1.975 | 0.369 | 0.222 | 0.101 | 97 | 88 | -19 | -51 | -78 | 2.27E-7 | | 6.68E-7 | 9.32E-6 |
| SF-539 | 1.082 | 3.095 | 3.018 | 2.891 | 1.206 | 0.825 | 0.289 | 96 | 90 | 6 | -24 | -73 | 2.99E-7 | | 1.61E-6 | 3.39E-5 |
| SNB-19 | 0.533 | 2.077 | 1.939 | 1.917 | 0.919 | 0.735 | 0.405 | 91 | 90 | 25 | 13 | -24 | 4.10E-7 | | 2.25E-5 | > 1.00E-4 |
| SNB-75 | 1.401 | 2.401 | 2.155 | 2.104 | 1.022 | 0.888 | 1.012 | 75 | 70 | -27 | -37 | -28 | 1.62E-7 | | 5.27E-7 | > 1.00E-4 |
| U251 | 0.420 | 2.056 | 1.985 | 1.884 | 0.828 | 0.556 | 0.274 | 96 | 89 | 25 | 8 | -35 | 4.09E-7 | | 1.56E-5 | > 1.00E-4 |
| Melanoma | | | | | | | | | | | | | | | | |
| LOX IMVI | 0.373 | 2.398 | 2.344 | 2.130 | 0.946 | 0.430 | 0.263 | 97 | 87 | 28 | 3 | -29 | 4.25E-7 | | 1.22E-5 | > 1.00E-4 |
| M14 | 0.589 | 2.384 | 2.335 | 2.027 | 0.807 | 0.517 | 0.359 | 97 | 80 | 12 | -12 | -39 | 2.77E-7 | | 3.13E-6 | > 1.00E-4 |
| MDA-MB-435 | 0.617 | 2.745 | 2.695 | 1.039 | 0.519 | 0.321 | 0.228 | 98 | 20 | -16 | -48 | -63 | 4.10E-8 | | 3.58E-7 | 1.35E-5 |
| SK-MEL-2 | 1.161 | 2.338 | 2.396 | 2.181 | 1.430 | 1.297 | 0.957 | 105 | 87 | 23 | 12 | -18 | 3.75E-7 | | 2.49E-5 | > 1.00E-4 |
| SK-MEL-28 | 0.662 | 2.212 | 2.210 | 1.944 | 1.508 | 1.100 | 0.962 | 100 | 83 | 55 | 28 | 19 | 1.49E-6 | | > 1.00E-4 | > 1.00E-4 |
| SK-MEL-5 | 0.845 | 3.277 | 3.261 | 3.241 | 1.638 | 1.322 | 1.305 | 99 | 99 | 33 | 20 | 19 | 5.45E-7 | | > 1.00E-4 | > 1.00E-4 |
| UACC-257 | 0.943 | 2.339 | 2.324 | 2.136 | 1.647 | 1.557 | 1.404 | 99 | 85 | 50 | 44 | 33 | 1.16E-6 | | > 1.00E-4 | > 1.00E-4 |
| UACC-62 | 0.669 | 2.691 | 2.516 | 2.067 | 1.004 | 0.694 | 0.443 | 91 | 69 | 17 | 1 | -34 | 2.31E-7 | | 1.08E-5 | > 1.00E-4 |
| Ovarian Cancer | | | | | | | | | | | | | | | | |
| IGROV1 | 0.783 | 2.406 | 2.347 | 2.015 | 1.295 | 0.970 | 0.823 | 96 | 76 | 32 | 12 | 2 | 3.83E-7 | | > 1.00E-4 | > 1.00E-4 |
| OVCAR-3 | 0.566 | 1.781 | 1.812 | 1.488 | 0.674 | 0.461 | 0.353 | 103 | 76 | 9 | -19 | -38 | 2.43E-7 | | 2.10E-6 | > 1.00E-4 |
| OVCAR-4 | 0.824 | 1.674 | 1.688 | 1.650 | 1.448 | 1.283 | 1.278 | 102 | 97 | 73 | 54 | 53 | > 1.00E-4 | | > 1.00E-4 | > 1.00E-4 |
| OVCAR-5 | 0.546 | 1.681 | 1.630 | 1.612 | 0.984 | 0.569 | 0.501 | 95 | 94 | 39 | 2 | -8 | 6.22E-7 | | 1.58E-5 | > 1.00E-4 |
| OVCAR-8 | 0.748 | 2.794 | 2.779 | 2.743 | 1.386 | 0.784 | 0.585 | 99 | 98 | 31 | 2 | -22 | 5.20E-7 | | 1.19E-5 | > 1.00E-4 |
| NCI/ADR-RES | 0.583 | 2.076 | 2.077 | 1.466 | 0.667 | 0.489 | 0.582 | 100 | 59 | 6 | -16 | | 1.48E-7 | | 1.81E-6 | > 1.00E-4 |
| SK-OV-3 | 1.046 | 2.136 | 2.105 | 2.068 | 1.626 | 1.428 | 1.009 | 97 | 94 | 53 | 35 | -4 | 1.50E-6 | | 8.10E-5 | > 1.00E-4 |
| Renal Cancer | | | | | | | | | | | | | | | | |
| 786-0 | 0.692 | 2.569 | 2.510 | 2.415 | 1.546 | 1.019 | 1.003 | 97 | 92 | 45 | 17 | 17 | 7.99E-7 | | > 1.00E-4 | > 1.00E-4 |
| A498 | 1.488 | 2.195 | 2.054 | 1.938 | 1.301 | 0.945 | 0.705 | 80 | 64 | -13 | -37 | -53 | 1.51E-7 | | 6.84E-7 | 6.85E-5 |
| ACHN | 0.395 | 1.870 | 1.889 | 1.685 | 1.014 | 0.638 | 0.523 | 101 | 87 | 42 | 16 | 9 | 6.65E-7 | | > 1.00E-4 | > 1.00E-4 |
| CAKI-1 | 0.828 | 2.664 | 2.458 | 1.806 | 1.468 | 1.282 | 1.353 | 89 | 53 | 35 | 25 | 29 | 1.50E-7 | | > 1.00E-4 | > 1.00E-4 |
| RXF 393 | 1.169 | 1.875 | 1.817 | 1.674 | 0.834 | 0.626 | 0.596 | 92 | 71 | -29 | -46 | -49 | 1.64E-7 | | 5.17E-7 | > 1.00E-4 |
| SN12C | 0.639 | 2.482 | 2.372 | 2.314 | 1.311 | 0.973 | 0.963 | 94 | 91 | 36 | 18 | 18 | 5.63E-7 | | > 1.00E-4 | > 1.00E-4 |
| TK-10 | 0.933 | 1.794 | 1.865 | 1.933 | 1.420 | 1.027 | 0.602 | 108 | 116 | 57 | 11 | -35 | 1.39E-6 | | 1.72E-5 | > 1.00E-4 |
| UO-31 | 1.469 | 3.003 | 2.861 | 2.727 | 2.027 | 1.778 | 1.824 | 91 | 82 | 36 | 20 | 23 | 5.03E-7 | | > 1.00E-4 | > 1.00E-4 |
| Prostate Cancer | | | | | | | | | | | | | | | | |
| PC-3 | 0.618 | 2.348 | 2.296 | 1.886 | 0.986 | 0.950 | 0.779 | 97 | 73 | 21 | 19 | 9 | 2.80E-7 | | > 1.00E-4 | > 1.00E-4 |
| DU-145 | 0.548 | 2.263 | 2.329 | 2.295 | 0.981 | 0.669 | 0.596 | 104 | 102 | 25 | 7 | 3 | 4.75E-7 | | > 1.00E-4 | > 1.00E-4 |
| Breast Cancer | | | | | | | | | | | | | | | | |
| MCF7 | 0.484 | 2.358 | 2.197 | 2.209 | 1.000 | 0.790 | 0.868 | 91 | 92 | 28 | 16 | 20 | 4.48E-7 | | > 1.00E-4 | > 1.00E-4 |
| MDA-MB-231/ATCC | 0.861 | 1.828 | 1.833 | 1.699 | 1.252 | 0.662 | 0.416 | 100 | 87 | 40 | -23 | -52 | 6.21E-7 | | 4.33E-6 | 8.69E-5 |
| HS 578T | 1.407 | 2.212 | 2.137 | 2.153 | 1.453 | 1.216 | 1.283 | 91 | 93 | 6 | -14 | -9 | 3.09E-7 | | 1.98E-6 | > 1.00E-4 |
| BT-549 | 1.122 | 2.407 | 2.334 | 2.203 | 1.585 | 1.394 | 1.146 | 94 | 84 | 36 | 21 | 2 | 5.12E-7 | | > 1.00E-4 | > 1.00E-4 |
| T-47D | 1.067 | 2.339 | 2.267 | 2.132 | 1.557 | 1.267 | 1.170 | 94 | 84 | 38 | 16 | 8 | 5.57E-7 | | > 1.00E-4 | > 1.00E-4 |
| MDA-MB-468 | 0.969 | 2.056 | 1.999 | 1.980 | 1.588 | 0.640 | 0.783 | 95 | 93 | 57 | -34 | -19 | 1.19E-6 | | 4.23E-6 | > 1.00E-4 |

Figure S29. NCI60 screening on compound **5**.

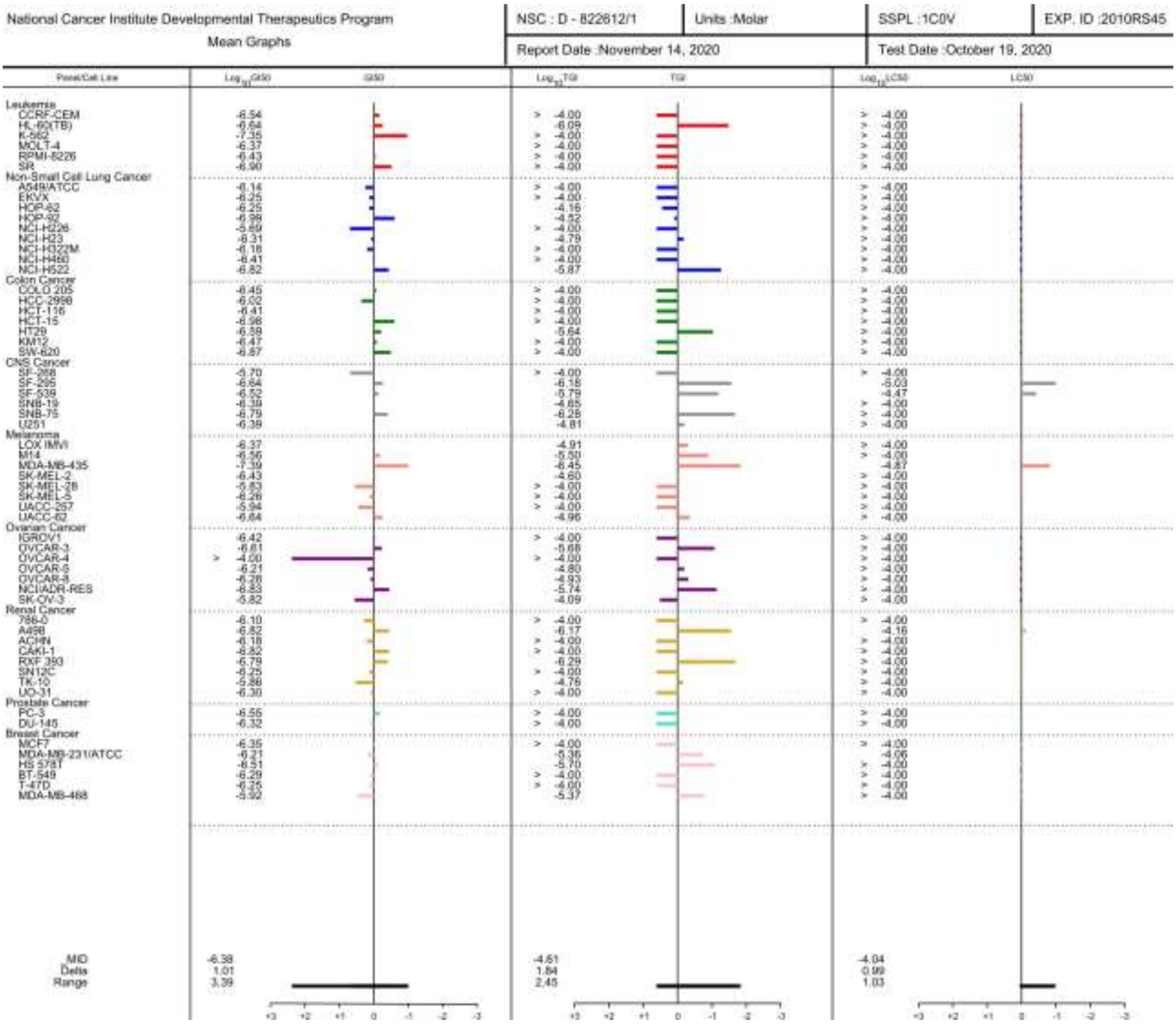


Figure S30. NCI60 screening on compound **5**.

# National Cancer Institute Developmental Therapeutics Program
## In-Vitro Testing Results

| NSC : D - 833304 / 1 | Experiment ID : 2205RS13 | Test Type : 08 | Units : Molar |
|---|---|---|---|
| Report Date : June 22, 2022 | Test Date : May 09, 2022 | QNS : | MC : |
| COMI : FR-11 | Stain Reagent : SRB Dual-Pass Related | SSPL : 1C0V | |

| | | | Log10 Concentration | | | | | | | | | | | | | |
|---|---|---|---|---|---|---|---|---|---|---|---|---|---|---|---|---|
| | Time | | Mean Optical Densities | | | | | Percent Growth | | | | | | | | |
| Panel/Cell Line | Zero | Ctrl | -8.0 | -7.0 | -6.0 | -5.0 | -4.0 | -8.0 | -7.0 | -6.0 | -5.0 | -4.0 | GI50 | TGI | LC50 |
| **Leukemia** | | | | | | | | | | | | | | | |
| CCRF-CEM | 0.522 | 1.936 | 1.899 | 1.668 | 0.532 | 0.447 | 0.496 | 97 | 81 | 1 | -14 | -5 | 2.43E-7 | 1.11E-6 | > 1.00E-4 |
| HL-60(TB) | 0.551 | 1.874 | 1.867 | 1.759 | 0.389 | 0.438 | 0.491 | 99 | 91 | -29 | -21 | -11 | 2.20E-7 | 5.70E-7 | > 1.00E-4 |
| K-562 | 0.171 | 1.452 | 1.481 | 0.686 | 0.247 | 0.202 | 0.252 | 102 | 40 | 6 | 2 | 6 | 6.94E-8 | > 1.00E-4 | > 1.00E-4 |
| MOLT-4 | 0.599 | 2.098 | 2.167 | 2.216 | 0.916 | 0.522 | 0.577 | 105 | 108 | 21 | -13 | -4 | 4.65E-7 | 4.18E-6 | > 1.00E-4 |
| RPMI-8226 | 0.700 | 2.713 | 2.574 | 2.325 | 0.877 | 0.557 | 0.606 | 93 | 81 | 9 | -21 | -13 | 2.67E-7 | 2.00E-6 | > 1.00E-4 |
| SR | 0.281 | 1.046 | 1.019 | 0.859 | 0.353 | 0.264 | 0.267 | 97 | 76 | 9 | -6 | -5 | 2.43E-7 | 4.06E-6 | > 1.00E-4 |
| **Non-Small Cell Lung Cancer** | | | | | | | | | | | | | | | |
| A549/ATCC | 0.308 | 1.725 | 1.739 | 1.684 | 0.677 | 0.404 | 0.394 | 101 | 97 | 26 | 7 | 6 | 4.60E-7 | > 1.00E-4 | > 1.00E-4 |
| EKVX | 0.716 | 2.147 | 2.036 | 1.907 | 1.411 | 0.941 | 0.852 | 92 | 83 | 49 | 16 | 9 | 9.08E-7 | > 1.00E-4 | > 1.00E-4 |
| HOP-62 | 1.044 | 2.612 | 2.410 | 2.383 | 1.416 | 1.021 | 0.713 | 87 | 85 | 24 | -2 | -32 | 3.75E-7 | 8.22E-6 | > 1.00E-4 |
| HOP-92 | 1.350 | 1.961 | 1.827 | 1.739 | 1.506 | 1.190 | 0.980 | 78 | 64 | 26 | -12 | -27 | 2.28E-7 | 4.82E-6 | > 1.00E-4 |
| NCI-H226 | 0.951 | 2.212 | 2.032 | 1.937 | 1.629 | 1.282 | 0.991 | 86 | 78 | 54 | 26 | 3 | 1.37E-6 | > 1.00E-4 | > 1.00E-4 |
| NCI-H23 | 0.473 | 1.568 | 1.485 | 1.447 | 0.754 | 0.607 | 0.445 | 92 | 89 | 26 | 12 | -6 | 4.12E-7 | 4.71E-5 | > 1.00E-4 |
| NCI-H322M | 0.730 | 1.974 | 1.826 | 1.814 | 1.384 | 0.771 | 0.637 | 88 | 87 | 53 | 3 | -13 | 1.13E-6 | 1.61E-5 | > 1.00E-4 |
| NCI-H460 | 0.292 | 2.674 | 2.792 | 2.693 | 0.679 | 0.272 | 0.177 | 105 | 101 | 16 | -7 | -39 | 3.99E-7 | 5.05E-6 | > 1.00E-4 |
| NCI-H522 | 1.043 | 2.699 | 2.620 | 1.869 | 0.686 | 0.996 | 0.786 | 95 | 50 | -34 | -5 | -25 | 9.95E-8 | 3.92E-7 | > 1.00E-4 |
| **Colon Cancer** | | | | | | | | | | | | | | | |
| COLO 205 | 0.677 | 2.005 | 2.008 | 2.056 | 0.592 | 0.335 | 0.198 | 100 | 104 | -13 | -51 | -71 | 2.90E-7 | 7.79E-7 | 9.69E-6 |
| HCC-2998 | 0.454 | 1.676 | 1.791 | 1.691 | 1.006 | 0.229 | 0.215 | 109 | 101 | 45 | -50 | -53 | 8.19E-7 | 3.00E-6 | 1.39E-5 |
| HCT-116 | 0.160 | 1.436 | 1.331 | 1.363 | 0.280 | 0.209 | 0.161 | 92 | 94 | 9 | 4 | 0 | 3.32E-7 | > 1.00E-4 | > 1.00E-4 |
| HCT-15 | 0.462 | 2.845 | 2.761 | 2.690 | 0.916 | 0.816 | 0.696 | 96 | 93 | 19 | 15 | 10 | 3.84E-7 | > 1.00E-4 | > 1.00E-4 |
| HT29 | 0.325 | 1.970 | 1.987 | 2.042 | 0.301 | 0.392 | 0.365 | 101 | 104 | -8 | 4 | 2 | 3.06E-7 | . | > 1.00E-4 |
| KM12 | 0.641 | 3.138 | 3.108 | 3.063 | 1.458 | 0.943 | 0.680 | 99 | 97 | 33 | 12 | 2 | 5.38E-7 | > 1.00E-4 | > 1.00E-4 |
| SW-620 | 0.340 | 2.441 | 2.389 | 2.065 | 0.764 | 0.466 | 0.434 | 98 | 82 | 20 | 6 | 4 | 3.30E-7 | > 1.00E-4 | > 1.00E-4 |
| **CNS Cancer** | | | | | | | | | | | | | | | |
| SF-268 | 0.941 | 2.726 | 2.712 | 2.621 | 1.838 | 1.181 | 1.057 | 99 | 94 | 50 | 13 | 6 | 1.01E-6 | > 1.00E-4 | > 1.00E-4 |
| SF-295 | 0.787 | 2.631 | 2.475 | 2.354 | 0.955 | 1.105 | 0.731 | 92 | 85 | 9 | 17 | -7 | 2.89E-7 | 5.08E-5 | > 1.00E-4 |
| SF-539 | 0.706 | 2.251 | 2.197 | 2.108 | 0.390 | 0.456 | 0.317 | 96 | 91 | -45 | -35 | -55 | 2.00E-7 | 4.67E-7 | 5.46E-5 |
| SNB-19 | 1.100 | 2.521 | 2.412 | 2.381 | 1.695 | 1.612 | 1.167 | 92 | 90 | 42 | 36 | 5 | 6.77E-7 | > 1.00E-4 | > 1.00E-4 |
| SNB-75 | 1.103 | 2.142 | 2.002 | 2.004 | 1.049 | 1.324 | 1.228 | 86 | 87 | -5 | 21 | 12 | 2.51E-7 | . | > 1.00E-4 |
| U251 | 0.316 | 1.503 | 1.487 | 1.425 | 0.507 | 0.357 | 0.323 | 99 | 93 | 16 | 3 | 1 | 3.64E-7 | > 1.00E-4 | > 1.00E-4 |
| **Melanoma** | | | | | | | | | | | | | | | |
| LOX IMVI | 0.396 | 2.565 | 2.318 | 2.150 | 0.913 | 0.303 | 0.153 | 89 | 81 | 24 | -23 | -61 | 3.48E-7 | 3.19E-6 | 5.01E-5 |
| MALME-3M | 0.793 | 1.691 | 1.622 | 1.519 | 1.060 | 1.028 | 0.892 | 92 | 81 | 30 | 26 | 11 | 4.01E-7 | > 1.00E-4 | > 1.00E-4 |
| M14 | 0.516 | 1.789 | 1.714 | 1.504 | 0.342 | 0.678 | 0.495 | 94 | 78 | -34 | 13 | -4 | 1.77E-7 | . | > 1.00E-4 |
| MDA-MB-435 | 0.417 | 2.136 | 2.179 | 0.767 | 0.233 | 0.506 | 0.383 | 102 | 20 | -44 | 5 | -8 | 4.36E-8 | . | > 1.00E-4 |
| SK-MEL-2 | 1.664 | 3.087 | 2.991 | 2.990 | 1.586 | 2.172 | 1.968 | 93 | 93 | -5 | 36 | 21 | 2.76E-7 | . | > 1.00E-4 |
| SK-MEL-28 | 0.893 | 2.325 | 2.269 | 2.142 | 1.584 | 1.200 | 1.180 | 96 | 87 | 48 | 21 | 20 | 9.01E-7 | > 1.00E-4 | > 1.00E-4 |
| SK-MEL-5 | 0.960 | 3.249 | 3.151 | 3.214 | 1.452 | 0.635 | 0.305 | 96 | 98 | 21 | -34 | -68 | 4.26E-7 | 2.44E-6 | 2.94E-5 |
| UACC-257 | 1.036 | 2.263 | 2.234 | 2.192 | 1.633 | 1.524 | 1.361 | 98 | 94 | 49 | 40 | 26 | 9.34E-7 | > 1.00E-4 | > 1.00E-4 |
| UACC-62 | 1.131 | 2.761 | 2.566 | 2.411 | 1.482 | 1.412 | 1.080 | 88 | 79 | 22 | 17 | -5 | 3.16E-7 | 6.18E-5 | > 1.00E-4 |
| **Ovarian Cancer** | | | | | | | | | | | | | | | |
| IGROV1 | 0.445 | 1.916 | 1.798 | 1.609 | 0.893 | 0.430 | 0.333 | 92 | 79 | 30 | -3 | -25 | 3.96E-7 | 7.95E-6 | > 1.00E-4 |
| OVCAR-3 | 0.521 | 1.810 | 1.804 | 1.719 | 0.480 | 0.487 | 0.346 | 100 | 93 | -8 | -7 | -34 | 2.67E-7 | 8.34E-7 | > 1.00E-4 |
| OVCAR-4 | 0.684 | 1.794 | 1.790 | 1.753 | 1.596 | 1.124 | 0.890 | 100 | 96 | 82 | 40 | 19 | 5.70E-6 | > 1.00E-4 | > 1.00E-4 |
| OVCAR-5 | 0.474 | 1.276 | 1.250 | 1.204 | 0.665 | 0.475 | 0.438 | 97 | 91 | 24 | 0 | -8 | 4.08E-7 | 1.04E-5 | > 1.00E-4 |
| OVCAR-8 | 0.499 | 2.344 | 2.282 | 2.223 | 0.705 | 0.356 | 0.330 | 97 | 93 | 11 | -29 | -34 | 3.37E-7 | 1.91E-6 | > 1.00E-4 |
| NCI/ADR-RES | 0.479 | 1.718 | 1.713 | 1.227 | 0.252 | 0.359 | 0.305 | 100 | 60 | -47 | -25 | -36 | 1.25E-7 | 3.63E-7 | > 1.00E-4 |
| SK-OV-3 | 1.197 | 2.063 | 2.104 | 2.060 | 1.512 | 1.409 | 1.118 | 105 | 100 | 36 | 24 | -7 | 6.08E-7 | 6.13E-5 | > 1.00E-4 |
| **Renal Cancer** | | | | | | | | | | | | | | | |
| 786-0 | 0.789 | 2.626 | 2.627 | 2.567 | 1.119 | 0.893 | 0.642 | 100 | 97 | 18 | 6 | -19 | 3.92E-7 | 1.71E-5 | > 1.00E-4 |
| A498 | 1.515 | 2.224 | 2.285 | 2.263 | 2.251 | 1.955 | 1.465 | 109 | 106 | 104 | 62 | -3 | 1.53E-5 | 8.89E-5 | > 1.00E-4 |
| ACHN | 0.450 | 1.899 | 1.818 | 1.751 | 1.012 | 0.637 | 0.496 | 94 | 90 | 39 | 13 | 3 | 6.02E-7 | > 1.00E-4 | > 1.00E-4 |
| CAKI-1 | 0.356 | 1.879 | 1.619 | 1.174 | 0.729 | 0.568 | 0.468 | 83 | 54 | 24 | 14 | 7 | 1.34E-7 | > 1.00E-4 | > 1.00E-4 |
| RXF 393 | 0.943 | 1.491 | 1.452 | 1.439 | 0.785 | 0.897 | 0.783 | 93 | 91 | -17 | -5 | -17 | 2.39E-7 | 6.97E-7 | > 1.00E-4 |
| SN12C | 0.933 | 2.775 | 2.719 | 2.607 | 1.471 | 0.641 | 0.375 | 97 | 91 | 29 | -31 | -60 | 4.60E-7 | 3.03E-6 | 4.52E-5 |
| TK-10 | 1.129 | 2.114 | 2.174 | 2.335 | 2.073 | 1.034 | 0.817 | 106 | 122 | 96 | -8 | -28 | 2.75E-6 | 8.30E-6 | > 1.00E-4 |
| UO-31 | 0.736 | 2.056 | 1.706 | 1.631 | 1.142 | 0.891 | 0.852 | 73 | 68 | 31 | 12 | 9 | 3.02E-7 | > 1.00E-4 | > 1.00E-4 |
| **Prostate Cancer** | | | | | | | | | | | | | | | |
| PC-3 | 0.618 | 2.158 | 1.990 | 1.883 | 1.018 | 0.909 | 0.683 | 89 | 82 | 26 | 19 | 4 | 3.73E-7 | > 1.00E-4 | > 1.00E-4 |
| DU-145 | 0.342 | 1.499 | 1.551 | 1.508 | 0.489 | 0.422 | 0.386 | 104 | 101 | 13 | 7 | 4 | 3.77E-7 | > 1.00E-4 | > 1.00E-4 |
| **Breast Cancer** | | | | | | | | | | | | | | | |
| MCF7 | 0.739 | 2.841 | 2.709 | 2.725 | 1.906 | 1.042 | 1.015 | 94 | 94 | 56 | 14 | 13 | 1.36E-6 | > 1.00E-4 | > 1.00E-4 |
| MDA-MB-231/ATCC | 0.532 | 1.196 | 1.137 | 1.138 | 0.711 | 0.368 | 0.348 | 91 | 91 | 27 | -31 | -35 | 4.39E-7 | 2.92E-6 | > 1.00E-4 |
| HS 578T | 1.533 | 2.572 | 2.413 | 2.352 | 1.446 | 1.692 | 1.708 | 85 | 79 | -6 | 15 | 17 | 2.19E-7 | . | > 1.00E-4 |
| BT-549 | 1.454 | 2.467 | 2.365 | 2.361 | 1.702 | 1.071 | 0.887 | 90 | 89 | 24 | -26 | -39 | 4.05E-7 | 3.03E-6 | > 1.00E-4 |
| T-47D | 1.022 | 2.200 | 2.065 | 1.977 | 1.825 | 1.013 | 1.059 | 89 | 81 | 68 | 0 | 3 | 1.83E-6 | . | > 1.00E-4 |
| MDA-MB-468 | 0.725 | 1.609 | 1.592 | 1.620 | 1.263 | 0.694 | 0.637 | 98 | 101 | 61 | -4 | -12 | 1.47E-6 | 8.58E-6 | > 1.00E-4 |

Figure S31. NCI60 screening on compound **6**.

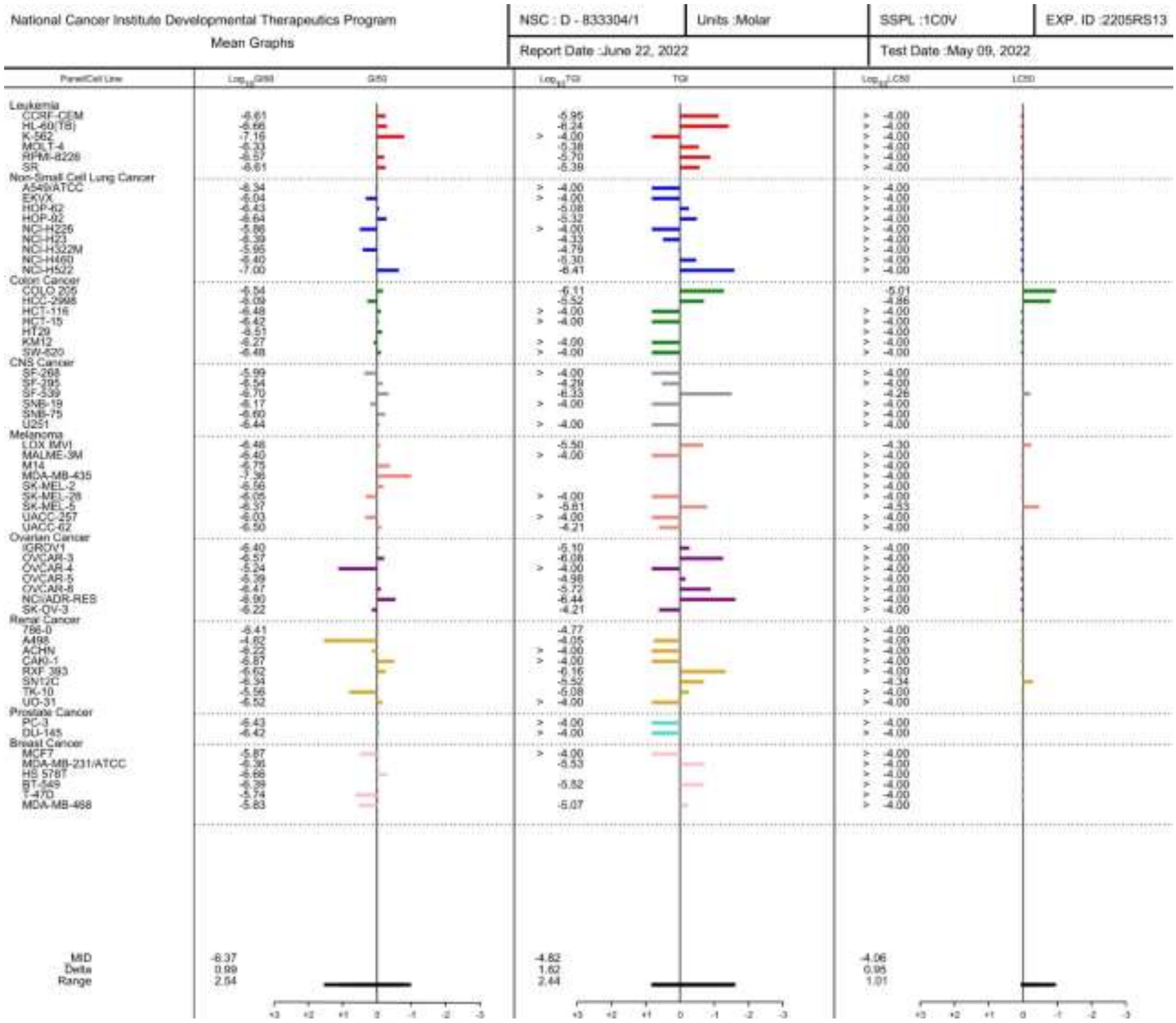

Figure S32. NCI60 screening on compound **6**.

# National Cancer Institute Developmental Therapeutics Program
## In-Vitro Testing Results

| | | | |
|---|---|---|---|
| NSC : D - 816687 / 1 | Experiment ID : 1909NS63 | Test Type : 08 | Units : Molar |
| Report Date : December 01, 2019 | Test Date : September 16, 2019 | QNS : | MC : |
| COMI : FR12 | Stain Reagent : SRB Dual-Pass Related | SSPL : 0JSG | |

| | | | Log10 Concentration | | | | | | | | | | | | | |
|---|---|---|---|---|---|---|---|---|---|---|---|---|---|---|---|---|
| | Time | | Mean Optical Densities | | | | | Percent Growth | | | | | | | | |
| Panel/Cell Line | Zero | Ctrl | -8.0 | -7.0 | -6.0 | -5.0 | -4.0 | -8.0 | -7.0 | -6.0 | -5.0 | -4.0 | GI50 | TGI | LC50 |
| Leukemia | | | | | | | | | | | | | | | |
| CCRF-CEM | 1.188 | 3.276 | 3.227 | 2.969 | 2.068 | 0.678 | 0.995 | 98 | 85 | 42 | -43 | -16 | 6.57E-7 | 3.13E-6 | > 1.00E-4 |
| HL-60(TB) | 0.883 | 3.130 | 3.223 | 3.236 | 2.688 | 0.934 | 1.061 | 104 | 105 | 80 | 2 | 8 | 2.45E-6 | > 1.00E-4 | > 1.00E-4 |
| K-562 | 0.077 | 1.873 | 2.001 | 1.926 | 0.306 | 0.196 | 0.249 | 107 | 103 | 13 | 7 | 10 | 3.86E-7 | > 1.00E-4 | > 1.00E-4 |
| MOLT-4 | 0.388 | 2.569 | 2.722 | 2.679 | 2.111 | 0.510 | 0.557 | 107 | 105 | 79 | 6 | 8 | 2.48E-6 | > 1.00E-4 | > 1.00E-4 |
| RPMI-8226 | 0.809 | 2.914 | 2.878 | 2.633 | 1.533 | 0.196 | 0.484 | 98 | 87 | 34 | -76 | -40 | 5.03E-7 | 2.05E-6 | . |
| SR | 0.265 | 2.062 | 2.142 | 2.095 | 0.651 | 0.319 | 0.429 | 104 | 102 | 21 | 3 | 9 | 4.41E-7 | > 1.00E-4 | > 1.00E-4 |
| Non-Small Cell Lung Cancer | | | | | | | | | | | | | | | |
| A549/ATCC | 0.310 | 2.065 | 2.130 | 2.061 | 1.494 | 0.422 | 0.564 | 104 | 100 | 67 | 6 | 14 | 1.93E-6 | > 1.00E-4 | > 1.00E-4 |
| EKVX | 0.952 | 2.067 | 1.788 | 1.735 | 1.669 | 1.015 | 1.197 | 75 | 70 | 64 | 6 | 22 | 1.75E-6 | > 1.00E-4 | > 1.00E-4 |
| HOP-62 | 0.758 | 2.564 | 2.310 | 2.304 | 2.108 | 0.597 | 0.971 | 86 | 86 | 75 | -21 | 12 | 1.81E-6 | . | > 1.00E-4 |
| HOP-92 | 1.427 | 2.002 | 1.881 | 1.756 | 1.542 | 1.274 | 1.099 | 79 | 57 | 20 | -11 | -23 | 1.56E-7 | 4.48E-6 | > 1.00E-4 |
| NCI-H226 | 0.872 | 2.371 | 2.113 | 2.087 | 1.871 | 0.722 | 1.120 | 83 | 81 | 67 | -17 | 17 | 1.58E-6 | . | > 1.00E-4 |
| NCI-H23 | 0.502 | 1.739 | 1.624 | 1.623 | 1.459 | 0.248 | 0.560 | 91 | 91 | 77 | -51 | 5 | 1.64E-6 | . | . |
| NCI-H322M | 1.587 | 3.125 | 3.034 | 3.000 | 3.003 | 1.552 | 2.259 | 94 | 92 | 92 | -2 | 44 | 2.79E-6 | . | > 1.00E-4 |
| NCI-H460 | 0.612 | 3.366 | 3.365 | 3.353 | 3.339 | 0.199 | 1.199 | 100 | 100 | 99 | -68 | 21 | 1.97E-6 | . | . |
| NCI-H522 | 0.943 | 2.736 | 2.608 | 2.540 | 1.255 | 0.382 | 0.696 | 93 | 89 | 17 | -60 | -26 | 3.51E-7 | 1.68E-6 | . |
| Colon Cancer | | | | | | | | | | | | | | | |
| COLO 205 | 0.484 | 2.211 | 2.208 | 2.282 | 1.827 | 0.187 | 0.507 | 100 | 104 | 78 | -61 | 1 | 1.58E-6 | . | . |
| HCC-2998 | 0.929 | 3.111 | 3.064 | 3.077 | 2.954 | 0.077 | 0.452 | 98 | 98 | 93 | -92 | -51 | 1.71E-6 | 3.18E-6 | 5.94E-6 |
| HCT-116 | 0.329 | 2.589 | 2.437 | 2.339 | 1.086 | 0.056 | 0.048 | 93 | 89 | 33 | -83 | -85 | 5.03E-7 | 1.94E-6 | 5.20E-6 |
| HCT-15 | 0.341 | 2.306 | 2.214 | 2.117 | 0.690 | 0.087 | 0.264 | 95 | 90 | 18 | -75 | -23 | 3.60E-7 | 1.56E-6 | . |
| HT29 | 0.230 | 1.878 | 1.965 | 2.010 | 0.368 | 0.066 | 0.216 | 105 | 108 | 8 | -71 | -6 | 3.82E-7 | 1.27E-6 | . |
| KM12 | 0.662 | 3.119 | 3.100 | 3.052 | 1.740 | 0.070 | 0.535 | 99 | 97 | 44 | -89 | -19 | 7.67E-7 | 2.13E-6 | . |
| SW-620 | 0.348 | 2.274 | 2.165 | 2.107 | 0.745 | 0.147 | 0.293 | 94 | 91 | 21 | -58 | -16 | 3.84E-7 | 1.83E-6 | . |
| CNS Cancer | | | | | | | | | | | | | | | |
| SF-268 | 0.871 | 2.443 | 2.340 | 2.314 | 2.245 | 1.051 | 1.236 | 93 | 92 | 87 | 11 | 23 | 3.11E-6 | > 1.00E-4 | > 1.00E-4 |
| SF-295 | 1.115 | 3.254 | 3.232 | 3.204 | 3.229 | 0.890 | 1.684 | 99 | 98 | 99 | -20 | 27 | 2.57E-6 | . | > 1.00E-4 |
| SF-539 | 1.275 | 3.138 | 3.032 | 3.000 | 3.021 | 0.848 | 1.169 | 94 | 93 | 94 | -33 | -8 | 2.21E-6 | 5.45E-6 | > 1.00E-4 |
| SNB-19 | 0.855 | 2.689 | 2.632 | 2.542 | 2.432 | 0.407 | 1.372 | 97 | 92 | 86 | -52 | 28 | 1.82E-6 | . | . |
| SNB-75 | 1.325 | 2.051 | 1.769 | 1.642 | 1.367 | 1.293 | 1.025 | 61 | 44 | 6 | -2 | -23 | 4.30E-8 | 5.04E-6 | > 1.00E-4 |
| U251 | 0.496 | 2.460 | 2.340 | 2.315 | 1.950 | 0.099 | 0.555 | 94 | 93 | 74 | -80 | 3 | 1.43E-6 | . | . |
| Melanoma | | | | | | | | | | | | | | | |
| LOX IMVI | 0.746 | 3.311 | 3.269 | 3.233 | 2.673 | 0.040 | 0.165 | 98 | 97 | 75 | -95 | -78 | 1.41E-6 | 2.77E-6 | 5.45E-6 |
| MALME-3M | 1.155 | 2.611 | 2.533 | 2.483 | 2.146 | 0.618 | 1.076 | 95 | 91 | 68 | -46 | -7 | 1.44E-6 | 3.93E-6 | > 1.00E-4 |
| M14 | 1.405 | 3.293 | 3.250 | 3.231 | 3.162 | 1.104 | 1.764 | 98 | 97 | 93 | -21 | 19 | 2.38E-6 | . | > 1.00E-4 |
| MDA-MB-435 | 0.555 | 2.577 | 2.640 | 2.501 | 0.231 | 0.087 | 0.400 | 103 | 96 | -58 | -84 | -28 | 1.99E-7 | 4.19E-7 | . |
| SK-MEL-2 | 1.374 | 3.096 | 3.032 | 3.044 | 2.807 | 1.042 | 1.635 | 96 | 97 | 83 | -24 | 15 | 2.04E-6 | . | > 1.00E-4 |
| SK-MEL-28 | 1.243 | 3.057 | 3.088 | 3.028 | 2.790 | 0.579 | 1.115 | 102 | 98 | 85 | -53 | -10 | 1.80E-6 | 4.12E-6 | . |
| SK-MEL-5 | 0.782 | 3.107 | 3.053 | 3.045 | 2.457 | 0.023 | 0.572 | 98 | 97 | 72 | -97 | -27 | 1.35E-6 | 2.67E-6 | . |
| UACC-257 | 1.427 | 3.090 | 3.056 | 3.057 | 2.866 | 1.187 | 1.750 | 98 | 98 | 87 | -17 | 19 | 2.26E-6 | . | > 1.00E-4 |
| UACC-62 | 0.828 | 2.987 | 2.922 | 2.798 | 1.742 | 0.137 | 0.361 | 97 | 91 | 42 | -84 | -56 | 6.97E-7 | 2.17E-6 | 5.42E-6 |
| Ovarian Cancer | | | | | | | | | | | | | | | |
| IGROV1 | 0.574 | 2.297 | 1.943 | 1.832 | 1.368 | 0.288 | 0.439 | 79 | 73 | 46 | -50 | -24 | 7.15E-7 | 3.02E-6 | > 1.00E-4 |
| OVCAR-3 | 0.466 | 1.654 | 1.645 | 1.594 | 0.431 | 0.093 | 0.204 | 99 | 95 | -8 | -80 | -56 | 2.75E-7 | 8.45E-7 | 3.85E-6 |
| OVCAR-4 | 1.192 | 2.714 | 2.720 | 2.679 | 2.356 | 0.987 | 1.251 | 100 | 98 | 76 | -17 | 4 | 1.92E-6 | . | > 1.00E-4 |
| OVCAR-5 | 0.776 | 1.694 | 1.639 | 1.678 | 1.656 | 0.348 | 0.806 | 94 | 98 | 96 | -55 | 3 | 2.01E-6 | . | . |
| OVCAR-8 | 0.859 | 3.201 | 3.169 | 3.199 | 2.837 | 1.077 | 1.242 | 99 | 100 | 84 | 9 | 16 | 2.87E-6 | > 1.00E-4 | > 1.00E-4 |
| NCI/ADR-RES | 0.658 | 2.381 | 2.429 | 2.314 | 1.037 | 0.636 | 0.799 | 103 | 96 | 22 | -3 | 8 | 4.19E-7 | . | > 1.00E-4 |
| SK-OV-3 | 2.179 | 2.810 | 2.699 | 2.686 | 2.698 | 2.465 | 2.643 | 82 | 80 | 82 | 45 | 74 | . | > 1.00E-4 | > 1.00E-4 |
| Renal Cancer | | | | | | | | | | | | | | | |
| 786-0 | 0.843 | 2.816 | 2.798 | 2.712 | 2.143 | 0.202 | 0.277 | 99 | 95 | 66 | -76 | -67 | 1.29E-6 | 2.91E-6 | 6.55E-6 |
| A498 | 2.140 | 2.888 | 2.837 | 2.692 | 2.609 | 1.471 | 1.442 | 93 | 74 | 63 | -31 | -33 | 1.36E-6 | 4.65E-6 | > 1.00E-4 |
| ACHN | 0.540 | 2.261 | 2.141 | 2.066 | 1.443 | 0.070 | 0.136 | 93 | 89 | 52 | -87 | -75 | 1.04E-6 | 2.38E-6 | 5.43E-6 |
| CAKI-1 | 1.046 | 2.809 | 2.395 | 2.290 | 1.645 | 0.753 | 1.282 | 77 | 71 | 34 | -28 | 13 | 3.65E-7 | . | > 1.00E-4 |
| RXF 393 | 1.250 | 1.809 | 1.786 | 1.714 | 1.449 | 0.093 | 0.654 | 96 | 83 | 36 | -93 | -48 | 4.95E-7 | 1.89E-6 | . |
| SN12C | 0.821 | 3.014 | 2.966 | 2.854 | 2.409 | 0.409 | 0.726 | 98 | 93 | 72 | -50 | -12 | 1.52E-6 | 3.90E-6 | . |
| TK-10 | 0.643 | 1.586 | 1.860 | 1.872 | 1.516 | 0.506 | 0.587 | 129 | 130 | 93 | -21 | -9 | 2.36E-6 | 6.50E-6 | > 1.00E-4 |
| UO-31 | 1.063 | 2.562 | 2.182 | 2.118 | 1.848 | 0.030 | 0.417 | 75 | 70 | 52 | -97 | -61 | 1.04E-6 | 2.24E-6 | 4.83E-6 |
| Prostate Cancer | | | | | | | | | | | | | | | |
| PC-3 | 0.566 | 2.048 | 1.945 | 1.879 | 1.030 | 0.174 | 0.262 | 93 | 89 | 31 | -69 | -54 | 4.72E-7 | 2.05E-6 | 6.43E-6 |
| DU-145 | 0.697 | 2.383 | 2.506 | 2.442 | 2.245 | 0.551 | 0.806 | 107 | 103 | 92 | -21 | 6 | 2.35E-6 | . | > 1.00E-4 |
| Breast Cancer | | | | | | | | | | | | | | | |
| MCF7 | 0.855 | 3.159 | 3.001 | 2.889 | 1.893 | 1.175 | 1.305 | 93 | 88 | 45 | 14 | 20 | 7.68E-7 | > 1.00E-4 | > 1.00E-4 |
| MDA-MB-231/ATCC | 0.902 | 2.063 | 1.905 | 1.801 | 1.603 | 0.569 | 0.700 | 86 | 77 | 60 | -37 | -22 | 1.28E-6 | 4.17E-6 | > 1.00E-4 |
| HS 578T | 1.654 | 2.743 | 2.651 | 2.650 | 2.565 | 1.874 | 1.854 | 92 | 92 | 84 | 20 | 18 | 3.39E-6 | > 1.00E-4 | > 1.00E-4 |
| BT-549 | 1.574 | 3.251 | 3.212 | 3.160 | 2.414 | 0.360 | 1.097 | 98 | 95 | 50 | -77 | -30 | 1.00E-6 | 2.47E-6 | . |
| T-47D | 0.899 | 2.085 | 1.973 | 1.901 | 1.155 | 0.808 | 0.854 | 90 | 84 | 22 | -10 | -5 | 3.53E-7 | 4.78E-6 | > 1.00E-4 |
| MDA-MB-468 | 0.921 | 2.116 | 2.183 | 2.147 | 1.254 | 0.412 | 0.742 | 106 | 103 | 28 | -55 | -19 | 5.05E-7 | 2.16E-6 | . |

Figure S33. NCI60 screening on compound **7**.

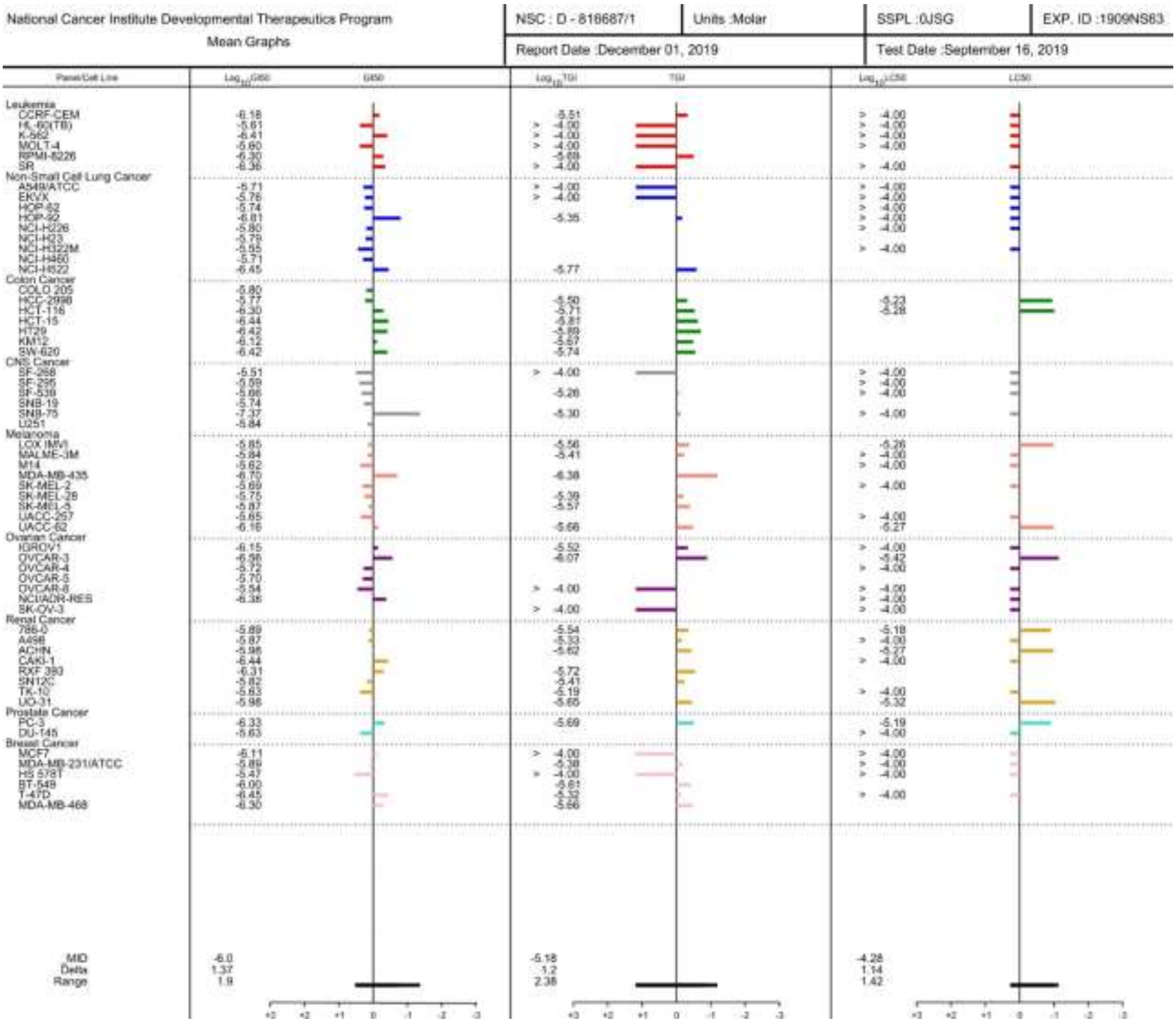


Figure S34. NCI60 screening on compound **7**.

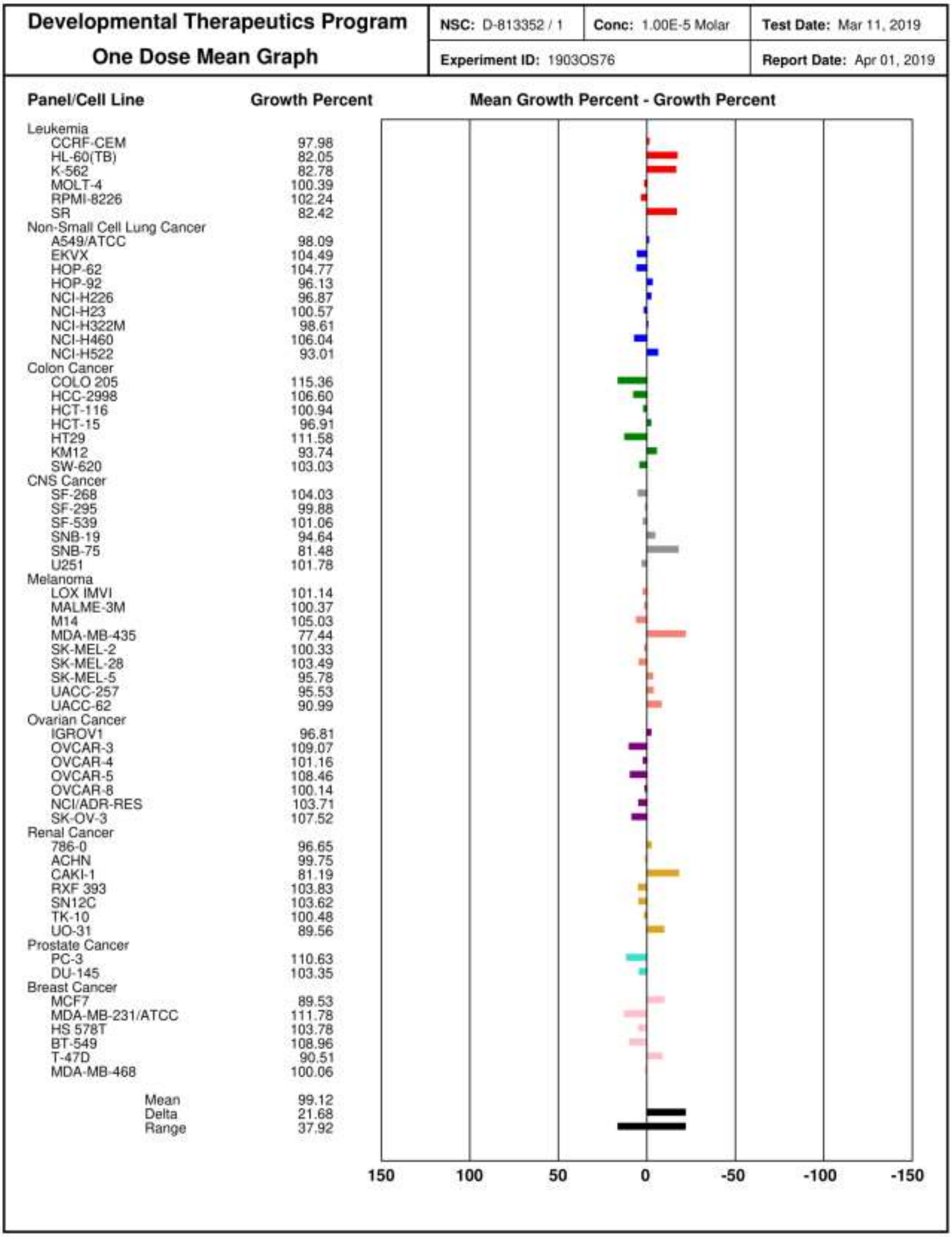


Figure S35. NCI60 screening on compound **8**.

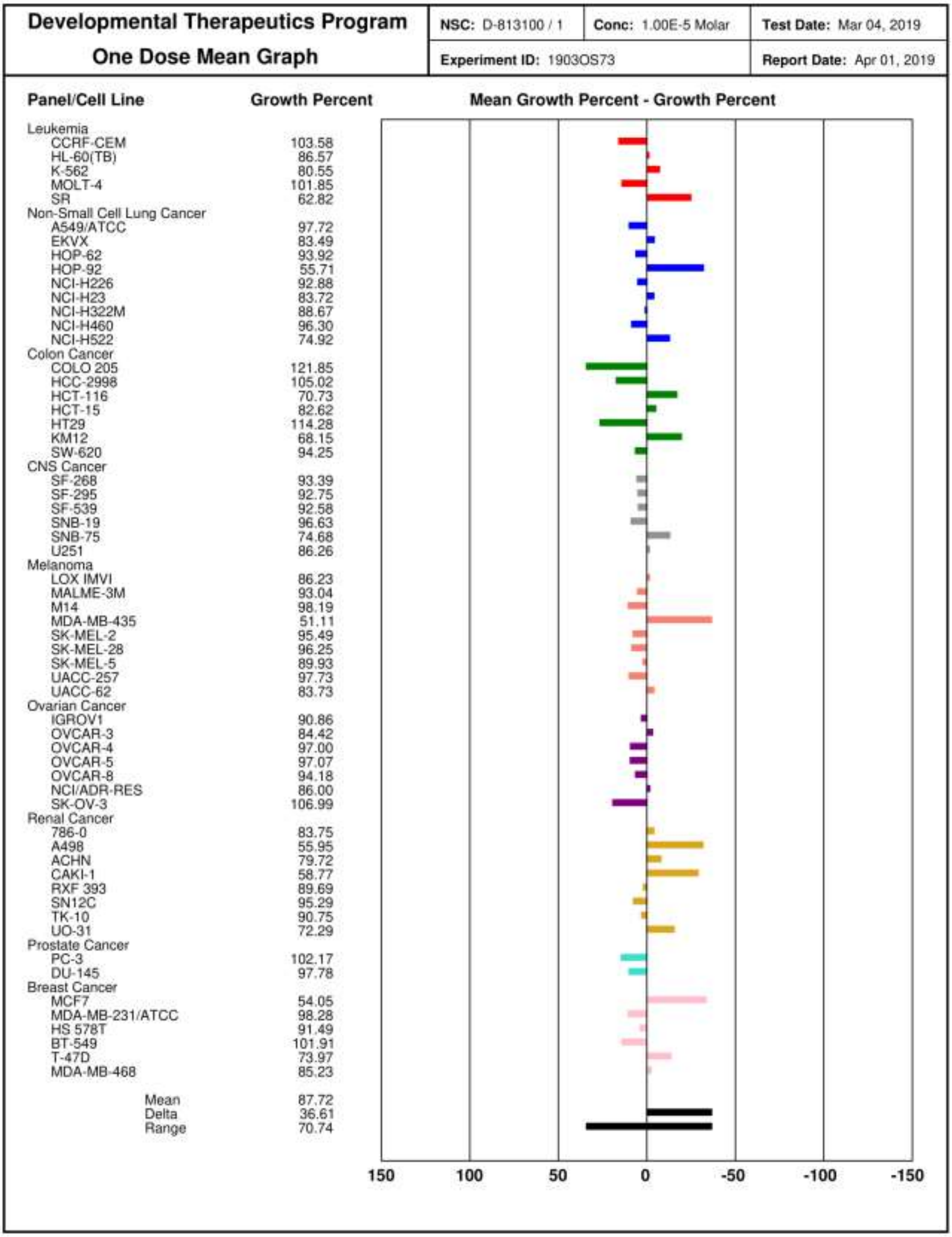


Figure S36. NCI60 screening on compound **9**.

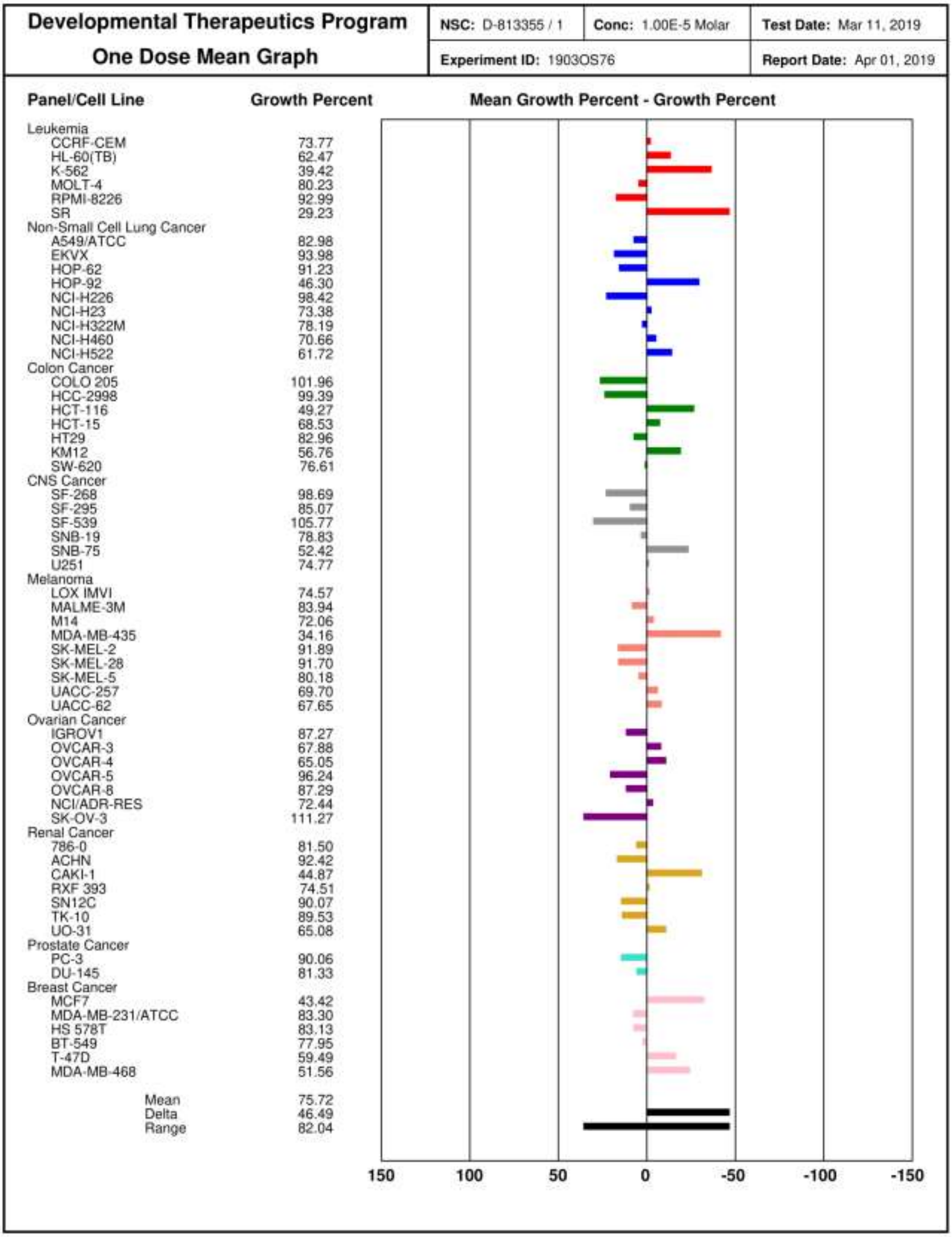


Figure S37. NCI60 screening on compound **10**.

# National Cancer Institute Developmental Therapeutics Program
## In-Vitro Testing Results

| | | | |
|---|---|---|---|
| NSC : D - 813098 / 1 | Experiment ID : 1904NS95 | Test Type : 08 | Units : Molar |
| Report Date : June 03, 2019 | Test Date : April 29, 2019 | QNS : | MC : |
| COMI : FR2 | Stain Reagent : SRB Dual-Pass Related | SSPL : 0JSG | |

| | | | Log10 Concentration | | | | | | | | | | | | |
|---|---|---|---|---|---|---|---|---|---|---|---|---|---|---|---|
| | Time | | Mean Optical Densities | | | | | Percent Growth | | | | | | | |
| Panel/Cell Line | Zero | Ctrl | -8.0 | -7.0 | -6.0 | -5.0 | -4.0 | -8.0 | -7.0 | -6.0 | -5.0 | -4.0 | GI50 | TGI | LC50 |
| **Leukemia** | | | | | | | | | | | | | | | |
| CCRF-CEM | 0.495 | 2.491 | 2.534 | 2.500 | 1.470 | 0.853 | 0.820 | 102 | 100 | 49 | 18 | 16 | 9.49E-7 | > 1.00E-4 | > 1.00E-4 |
| HL-60(TB) | 1.121 | 3.341 | 3.366 | 3.331 | 1.807 | 1.172 | 1.026 | 101 | 100 | 31 | 2 | -9 | 5.27E-7 | 1.62E-5 | > 1.00E-4 |
| K-562 | 0.276 | 2.546 | 2.576 | 2.441 | 0.557 | 0.358 | 0.421 | 101 | 95 | 12 | 4 | 6 | 3.52E-7 | > 1.00E-4 | > 1.00E-4 |
| MOLT-4 | 0.736 | 3.191 | 3.216 | 3.181 | 1.719 | 0.889 | 0.868 | 101 | 100 | 40 | 6 | 5 | 6.80E-7 | > 1.00E-4 | > 1.00E-4 |
| RPMI-8226 | 0.952 | 2.780 | 2.870 | 2.872 | 2.172 | 1.266 | 1.232 | 105 | 105 | 67 | 17 | 15 | 2.18E-6 | > 1.00E-4 | > 1.00E-4 |
| SR | 0.263 | 0.863 | 0.888 | 0.800 | 0.452 | 0.400 | 0.349 | 104 | 90 | 31 | 23 | 14 | 4.79E-7 | > 1.00E-4 | > 1.00E-4 |
| **Non-Small Cell Lung Cancer** | | | | | | | | | | | | | | | |
| A549/ATCC | 0.378 | 2.201 | 2.176 | 2.094 | 1.446 | 0.503 | 0.485 | 99 | 94 | 59 | 7 | 6 | 1.46E-6 | > 1.00E-4 | > 1.00E-4 |
| EKVX | 0.793 | 2.402 | 2.297 | 2.222 | 1.963 | 0.987 | 1.003 | 94 | 89 | 73 | 12 | 13 | 2.37E-6 | > 1.00E-4 | > 1.00E-4 |
| HOP-62 | 0.675 | 2.390 | 2.393 | 2.301 | 2.029 | 1.059 | 0.791 | 100 | 95 | 79 | 22 | 7 | 3.25E-6 | > 1.00E-4 | > 1.00E-4 |
| HOP-92 | 1.309 | 1.821 | 1.752 | 1.676 | 1.526 | 0.776 | 0.896 | 87 | 72 | 42 | -41 | -32 | 5.46E-7 | 3.23E-6 | > 1.00E-4 |
| NCI-H226 | 0.707 | 1.367 | 1.400 | 1.311 | 1.234 | 0.805 | 0.669 | 105 | 91 | 80 | 15 | -5 | 2.87E-6 | 5.38E-5 | > 1.00E-4 |
| NCI-H23 | 0.639 | 2.087 | 2.010 | 2.030 | 1.454 | 0.549 | 0.511 | 95 | 96 | 56 | -14 | -20 | 1.23E-6 | 6.29E-6 | > 1.00E-4 |
| NCI-H322M | 0.743 | 2.054 | 1.955 | 1.895 | 1.852 | 0.756 | 0.756 | 92 | 88 | 85 | 1 | 1 | 2.59E-6 | > 1.00E-4 | > 1.00E-4 |
| NCI-H460 | 0.182 | 1.386 | 1.389 | 1.391 | 0.674 | 0.145 | 0.140 | 100 | 100 | 41 | -21 | -23 | 7.03E-7 | 4.62E-6 | > 1.00E-4 |
| NCI-H522 | 0.894 | 2.489 | 2.427 | 2.164 | 1.178 | 1.125 | 1.050 | 96 | 80 | 18 | 14 | 10 | 3.01E-7 | > 1.00E-4 | > 1.00E-4 |
| **Colon Cancer** | | | | | | | | | | | | | | | |
| COLO 205 | 0.568 | 2.443 | 2.426 | 2.467 | 1.410 | 0.846 | 0.719 | 99 | 101 | 45 | 15 | 8 | 8.11E-7 | > 1.00E-4 | > 1.00E-4 |
| HCC-2998 | 0.975 | 2.902 | 2.831 | 2.733 | 2.364 | 0.855 | 0.919 | 96 | 91 | 72 | -12 | -6 | 1.83E-6 | 7.14E-6 | > 1.00E-4 |
| HCT-116 | 0.286 | 2.380 | 2.374 | 2.381 | 0.939 | 0.190 | 0.198 | 100 | 100 | 31 | -34 | -31 | 5.33E-7 | 3.02E-6 | > 1.00E-4 |
| HCT-15 | 0.259 | 1.779 | 1.707 | 1.588 | 0.644 | 0.339 | 0.335 | 95 | 87 | 25 | 5 | 5 | 4.01E-7 | > 1.00E-4 | > 1.00E-4 |
| HT29 | 0.367 | 2.331 | 2.335 | 2.394 | 0.688 | 0.536 | 0.512 | 100 | 103 | 16 | 9 | 7 | 4.10E-7 | > 1.00E-4 | > 1.00E-4 |
| KM12 | 0.535 | 2.165 | 2.193 | 2.188 | 1.016 | 0.453 | 0.417 | 102 | 101 | 30 | -15 | -22 | 5.19E-7 | 4.54E-6 | > 1.00E-4 |
| SW-620 | 0.293 | 1.774 | 1.723 | 1.647 | 0.626 | 0.344 | 0.325 | 97 | 91 | 22 | 3 | 2 | 3.99E-7 | > 1.00E-4 | > 1.00E-4 |
| **CNS Cancer** | | | | | | | | | | | | | | | |
| SF-268 | 0.638 | 1.741 | 1.735 | 1.743 | 1.437 | 0.757 | 0.696 | 100 | 100 | 72 | 11 | 5 | 2.31E-6 | > 1.00E-4 | > 1.00E-4 |
| SF-295 | 0.883 | 3.189 | 3.083 | 3.072 | 2.654 | 0.991 | 0.759 | 95 | 95 | 77 | 5 | -14 | 2.35E-6 | 1.78E-5 | > 1.00E-4 |
| SF-539 | 0.745 | 2.381 | 2.235 | 2.253 | 1.993 | 0.380 | 0.300 | 91 | 92 | 76 | -49 | -60 | 1.62E-6 | 4.06E-6 | 1.22E-5 |
| SNB-19 | 0.695 | 2.274 | 2.253 | 2.185 | 1.858 | 0.865 | 0.688 | 99 | 94 | 74 | 11 | -1 | 2.38E-6 | 8.21E-5 | > 1.00E-4 |
| SNB-75 | 1.037 | 1.948 | 1.801 | 1.785 | 0.831 | 0.802 | 0.738 | 84 | 82 | -20 | -23 | -29 | 2.06E-7 | 6.38E-7 | > 1.00E-4 |
| U251 | 0.296 | 1.721 | 1.634 | 1.613 | 1.198 | 0.447 | 0.356 | 94 | 92 | 63 | 11 | 4 | 1.79E-6 | > 1.00E-4 | > 1.00E-4 |
| **Melanoma** | | | | | | | | | | | | | | | |
| LOX IMVI | 0.244 | 1.855 | 1.839 | 1.799 | 0.959 | 0.309 | 0.271 | 99 | 97 | 44 | 4 | 2 | 7.80E-7 | > 1.00E-4 | > 1.00E-4 |
| MALME-3M | 0.712 | 1.855 | 1.808 | 1.724 | 1.199 | 0.854 | 0.744 | 96 | 89 | 43 | 12 | 3 | 6.90E-7 | > 1.00E-4 | > 1.00E-4 |
| M14 | 0.618 | 2.191 | 2.173 | 2.141 | 1.040 | 0.503 | 0.387 | 99 | 97 | 27 | -19 | -37 | 4.67E-7 | 3.89E-6 | > 1.00E-4 |
| MDA-MB-435 | 0.505 | 2.081 | 2.026 | 1.772 | 0.308 | 0.248 | 0.181 | 97 | 80 | -39 | -51 | -64 | 1.80E-7 | 4.71E-7 | 8.40E-6 |
| SK-MEL-2 | 1.287 | 2.564 | 2.618 | 2.513 | 1.792 | 1.380 | 1.107 | 104 | 96 | 40 | 7 | -14 | 6.53E-7 | 2.19E-5 | > 1.00E-4 |
| SK-MEL-28 | 0.739 | 2.242 | 2.223 | 2.194 | 1.627 | 1.144 | 0.976 | 99 | 97 | 59 | 27 | 16 | 1.92E-6 | > 1.00E-4 | > 1.00E-4 |
| SK-MEL-5 | 1.015 | 3.139 | 3.083 | 3.054 | 2.018 | 0.844 | 0.798 | 97 | 96 | 47 | -17 | -21 | 8.77E-7 | 5.46E-6 | > 1.00E-4 |
| UACC-257 | 1.272 | 2.713 | 2.674 | 2.607 | 2.456 | 2.185 | 1.969 | 97 | 93 | 82 | 63 | 48 | 7.78E-5 | > 1.00E-4 | > 1.00E-4 |
| UACC-62 | 0.958 | 3.083 | 2.944 | 2.892 | 1.669 | 1.029 | 0.923 | 93 | 91 | 33 | 3 | -4 | 5.16E-7 | 2.99E-5 | > 1.00E-4 |
| **Ovarian Cancer** | | | | | | | | | | | | | | | |
| IGROV1 | 0.540 | 2.196 | 2.125 | 2.042 | 1.277 | 0.561 | 0.515 | 96 | 91 | 44 | 1 | -5 | 7.59E-7 | 1.63E-5 | > 1.00E-4 |
| OVCAR-3 | 0.473 | 1.387 | 1.460 | 1.432 | 0.449 | 0.366 | 0.268 | 108 | 105 | -5 | -23 | -43 | 3.16E-7 | 8.99E-7 | > 1.00E-4 |
| OVCAR-4 | 0.711 | 1.518 | 1.479 | 1.456 | 1.195 | 0.928 | 0.802 | 95 | 92 | 60 | 27 | 11 | 2.00E-6 | > 1.00E-4 | > 1.00E-4 |
| OVCAR-5 | 0.540 | 1.459 | 1.439 | 1.434 | 1.301 | 0.309 | 0.345 | 98 | 97 | 83 | -43 | -36 | 1.82E-6 | 4.56E-6 | > 1.00E-4 |
| OVCAR-8 | 0.450 | 2.356 | 2.313 | 2.276 | 1.682 | 0.738 | 0.690 | 98 | 96 | 65 | 15 | 13 | 1.97E-6 | > 1.00E-4 | > 1.00E-4 |
| NCI/ADR-RES | 0.624 | 2.104 | 2.110 | 2.015 | 0.871 | 0.531 | 0.569 | 100 | 94 | 17 | -15 | -9 | 3.71E-7 | 3.36E-6 | > 1.00E-4 |
| SK-OV-3 | 0.868 | 2.063 | 2.130 | 2.028 | 1.898 | 1.212 | 1.066 | 106 | 97 | 86 | 29 | 17 | 4.27E-6 | > 1.00E-4 | > 1.00E-4 |
| **Renal Cancer** | | | | | | | | | | | | | | | |
| 786-0 | 0.555 | 2.331 | 2.311 | 2.125 | 1.868 | 0.762 | 0.568 | 99 | 88 | 74 | 12 | 1 | 2.42E-6 | > 1.00E-4 | > 1.00E-4 |
| A498 | 1.210 | 1.976 | 2.002 | 1.922 | 1.655 | 0.765 | 0.671 | 103 | 93 | 58 | -37 | -45 | 1.22E-6 | 4.10E-6 | > 1.00E-4 |
| ACHN | 0.370 | 1.600 | 1.614 | 1.643 | 1.057 | 0.456 | 0.404 | 101 | 103 | 56 | 7 | 3 | 1.31E-6 | > 1.00E-4 | > 1.00E-4 |
| CAKI-1 | 0.773 | 2.733 | 2.626 | 2.525 | 1.633 | 0.786 | 0.711 | 95 | 89 | 44 | 1 | -8 | 7.33E-7 | 1.18E-5 | > 1.00E-4 |
| RXF 393 | 0.776 | 1.471 | 1.501 | 1.395 | 1.181 | 0.666 | 0.514 | 104 | 89 | 58 | -14 | -34 | 1.30E-6 | 6.37E-6 | > 1.00E-4 |
| SN12C | 0.651 | 2.476 | 2.411 | 2.404 | 1.882 | 0.769 | 0.718 | 96 | 96 | 67 | 6 | 4 | 1.93E-6 | > 1.00E-4 | > 1.00E-4 |
| TK-10 | 0.767 | 1.806 | 1.728 | 1.665 | 1.670 | 0.907 | 0.717 | 92 | 86 | 87 | 13 | -7 | 3.18E-6 | 4.72E-5 | > 1.00E-4 |
| UO-31 | 0.642 | 1.811 | 1.672 | 1.636 | 1.483 | 0.552 | 0.595 | 88 | 85 | 72 | -14 | -7 | 1.80E-6 | 6.87E-6 | > 1.00E-4 |
| **Prostate Cancer** | | | | | | | | | | | | | | | |
| PC-3 | 0.618 | 2.322 | 2.305 | 2.212 | 1.651 | 0.796 | 0.743 | 99 | 94 | 61 | 10 | 7 | 1.63E-6 | > 1.00E-4 | > 1.00E-4 |
| DU-145 | 0.389 | 1.265 | 1.256 | 1.223 | 0.936 | 0.269 | 0.247 | 99 | 95 | 62 | -31 | -37 | 1.36E-6 | 4.67E-6 | > 1.00E-4 |
| **Breast Cancer** | | | | | | | | | | | | | | | |
| MCF7 | 0.576 | 2.773 | 2.618 | 2.604 | 1.313 | 0.861 | 0.803 | 93 | 92 | 34 | 13 | 10 | 5.24E-7 | > 1.00E-4 | > 1.00E-4 |
| MDA-MB-231/ATCC | 0.752 | 1.655 | 1.638 | 1.582 | 1.342 | 0.402 | 0.372 | 98 | 92 | 65 | -47 | -51 | 1.37E-6 | 3.84E-6 | 7.12E-5 |
| HS 578T | 0.857 | 1.609 | 1.530 | 1.538 | 1.375 | 0.716 | 0.741 | 89 | 91 | 69 | -16 | -14 | 1.66E-6 | 6.41E-6 | > 1.00E-4 |
| BT-549 | 1.598 | 2.497 | 2.515 | 2.595 | 2.186 | 1.821 | 1.447 | 102 | 111 | 65 | 25 | -9 | 2.39E-6 | 5.29E-5 | > 1.00E-4 |
| T-47D | 1.002 | 2.571 | 2.448 | 2.357 | 1.544 | 1.283 | 1.251 | 92 | 86 | 35 | 18 | 16 | 5.03E-7 | > 1.00E-4 | > 1.00E-4 |
| MDA-MB-468 | 0.819 | 1.859 | 1.844 | 1.749 | 1.084 | 0.734 | 0.653 | 99 | 89 | 25 | -10 | -20 | 4.14E-7 | 5.12E-6 | > 1.00E-4 |

Figure S38. NCI60 screening on compound **11**.

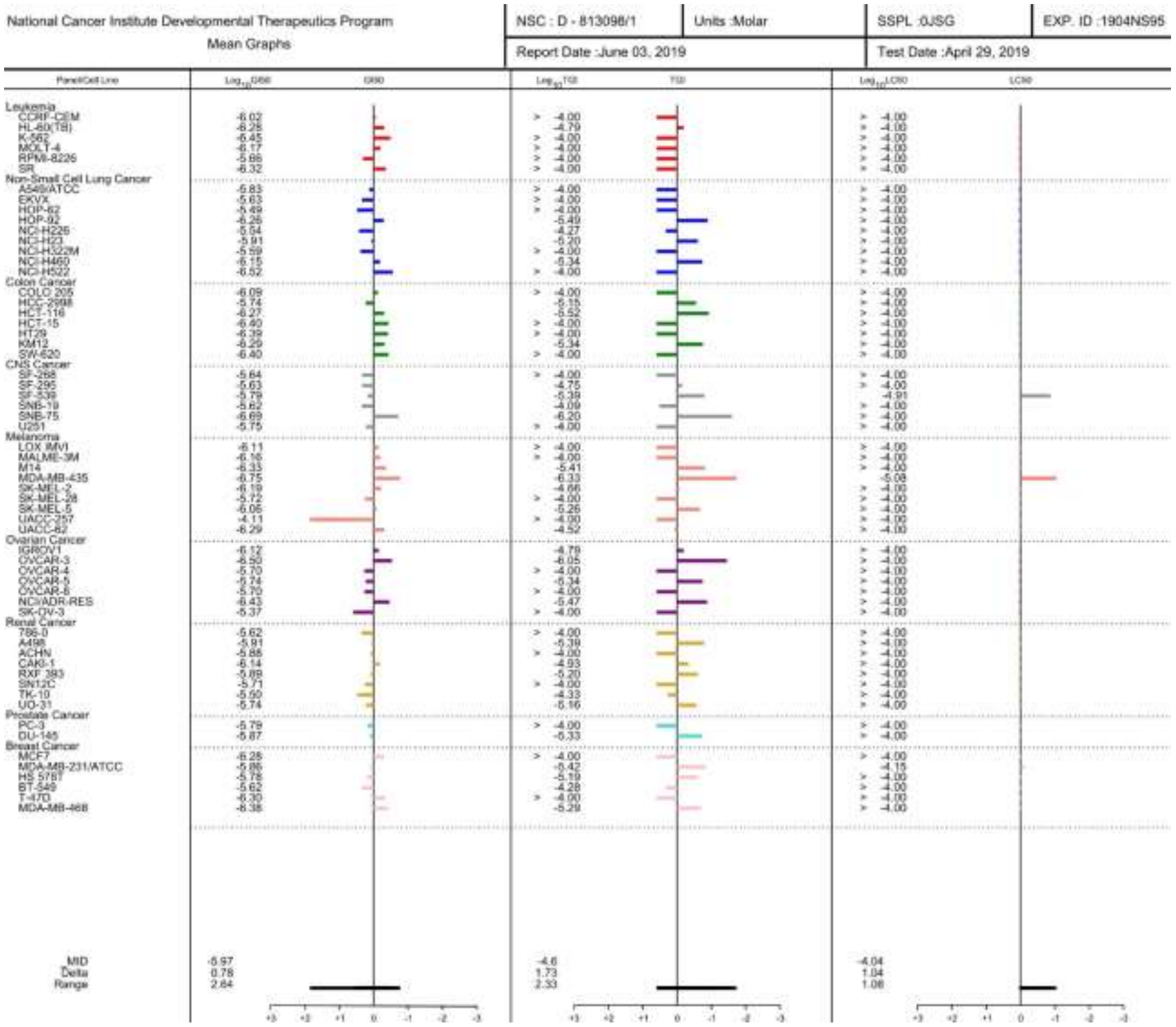


Figure S39. NCI60 screening on compound **11**.

# National Cancer Institute Developmental Therapeutics Program
## In-Vitro Testing Results

| | | | |
|---|---|---|---|
| NSC : D - 813353 / 1 | Experiment ID : 1907RS38 | Test Type : 08 | Units : Molar |
| Report Date : September 02, 2019 | Test Date : July 22, 2019 | QNS : | MC : |
| COMI : FR5 | Stain Reagent : SRB Dual-Pass Related | SSPL : 0JSG | |

| | | | Log10 Concentration | | | | | | | | | | | | | |
|---|---|---|---|---|---|---|---|---|---|---|---|---|---|---|---|---|
| | Time | | Mean Optical Densities | | | | | Percent Growth | | | | | | | | |
| Panel/Cell Line | Zero | Ctrl | -8.0 | -7.0 | -6.0 | -5.0 | -4.0 | -8.0 | -7.0 | -6.0 | -5.0 | -4.0 | GI50 | TGI | LC50 |
| **Leukemia** | | | | | | | | | | | | | | | |
| CCRF-CEM | 0.527 | 2.608 | 2.625 | 2.557 | 0.754 | 0.579 | 0.556 | 101 | 98 | 11 | 2 | 1 | 3.54E-7 | > 1.00E-4 | > 1.00E-4 |
| HL-60(TB) | 0.804 | 2.663 | 2.536 | 2.592 | 0.687 | 0.722 | 0.714 | 93 | 96 | -15 | -10 | -11 | 2.61E-7 | 7.39E-7 | > 1.00E-4 |
| K-562 | 0.208 | 2.040 | 2.004 | 1.297 | 0.426 | 0.350 | 0.253 | 98 | 59 | 12 | 8 | 2 | 1.58E-7 | > 1.00E-4 | > 1.00E-4 |
| MOLT-4 | 0.620 | 2.587 | 2.483 | 2.465 | 0.910 | 0.611 | 0.655 | 95 | 94 | 15 | -2 | 2 | 3.58E-7 | . | > 1.00E-4 |
| RPMI-8226 | 0.822 | 2.605 | 2.552 | 2.611 | 0.975 | 0.719 | 0.562 | 97 | 100 | 9 | -13 | -32 | 3.54E-7 | 2.55E-6 | > 1.00E-4 |
| SR | 0.312 | 1.138 | 1.006 | 0.708 | 0.449 | 0.331 | 0.250 | 84 | 48 | 17 | 2 | -20 | 8.78E-8 | 1.26E-5 | > 1.00E-4 |
| **Non-Small Cell Lung Cancer** | | | | | | | | | | | | | | | |
| A549/ATCC | 0.510 | 2.246 | 2.147 | 2.150 | 1.166 | 0.724 | 0.376 | 94 | 94 | 38 | 12 | -26 | 6.09E-7 | 2.08E-5 | > 1.00E-4 |
| EKVX | 0.862 | 2.591 | 2.413 | 2.506 | 1.661 | 1.197 | 0.361 | 90 | 95 | 46 | 19 | -58 | 8.37E-7 | 1.78E-5 | 7.84E-5 |
| HOP-92 | 1.006 | 1.665 | 1.545 | 1.551 | 1.263 | 0.788 | 0.662 | 82 | 83 | 39 | -22 | -34 | 5.58E-7 | 4.38E-6 | > 1.00E-4 |
| NCI-H226 | 0.990 | 1.713 | 1.670 | 1.721 | 1.492 | 0.865 | 0.657 | 94 | 101 | 69 | -13 | -34 | 1.72E-6 | 7.01E-6 | > 1.00E-4 |
| NCI-H23 | 0.548 | 1.811 | 1.734 | 1.890 | 1.010 | 0.703 | 0.026 | 94 | 106 | 37 | 12 | -95 | 6.42E-7 | 1.30E-5 | 3.79E-5 |
| NCI-H322M | 0.777 | 2.110 | 2.031 | 2.081 | 1.552 | 0.762 | 0.238 | 94 | 98 | 58 | -2 | -69 | 1.37E-6 | 9.29E-6 | 5.15E-5 |
| NCI-H460 | 0.224 | 2.110 | 2.013 | 2.071 | 0.360 | 0.185 | 0.039 | 95 | 98 | 7 | -18 | -83 | 3.38E-7 | 1.95E-6 | 3.14E-5 |
| NCI-H522 | 1.148 | 2.638 | 2.465 | 2.075 | 1.442 | 1.304 | 0.578 | 88 | 62 | 20 | 10 | -50 | 1.94E-7 | 1.49E-5 | > 1.00E-4 |
| **Colon Cancer** | | | | | | | | | | | | | | | |
| COLO 205 | 0.509 | 2.122 | 2.077 | 2.232 | 0.482 | 0.319 | 0.174 | 97 | 107 | -5 | -37 | -66 | 3.21E-7 | 8.97E-7 | 2.78E-5 |
| HCC-2998 | 0.853 | 2.716 | 2.394 | 2.702 | 2.086 | 0.784 | 0.082 | 83 | 99 | 66 | -8 | -90 | 1.65E-6 | 7.78E-6 | 3.23E-5 |
| HCT-116 | 0.264 | 2.145 | 2.124 | 1.989 | 0.461 | 0.315 | 0.027 | 99 | 92 | 10 | 3 | -90 | 3.26E-7 | 1.07E-5 | 3.70E-5 |
| HCT-15 | 0.318 | 2.347 | 2.130 | 1.714 | 0.610 | 0.344 | 0.110 | 89 | 69 | 14 | 1 | -65 | 2.21E-7 | 1.05E-5 | 5.87E-5 |
| HT29 | 0.232 | 1.655 | 1.628 | 1.711 | 0.358 | 0.280 | 0.094 | 98 | 104 | 9 | 3 | -60 | 3.69E-7 | 1.13E-5 | 7.02E-5 |
| KM12 | 0.483 | 2.313 | 2.341 | 2.258 | 0.673 | 0.573 | 0.097 | 102 | 97 | 10 | 5 | -80 | 3.49E-7 | 1.14E-5 | 4.43E-5 |
| SW-620 | 0.392 | 2.530 | 2.439 | 2.393 | 0.729 | 0.485 | 0.117 | 96 | 94 | 16 | 4 | -70 | 3.63E-7 | 1.14E-5 | 5.35E-5 |
| **CNS Cancer** | | | | | | | | | | | | | | | |
| SF-268 | 0.694 | 2.139 | 2.042 | 2.054 | 1.260 | 0.821 | 0.190 | 93 | 94 | 39 | 9 | -73 | 6.34E-7 | 1.28E-5 | 5.26E-5 |
| SF-295 | 1.049 | 3.326 | 3.247 | 3.334 | 1.521 | 0.971 | 0.209 | 97 | 100 | 21 | -7 | -80 | 4.29E-7 | 5.43E-6 | 3.85E-5 |
| SF-539 | 0.696 | 2.450 | 2.342 | 2.410 | 0.640 | 0.555 | 0.052 | 94 | 98 | -8 | -20 | -93 | 2.82E-7 | 8.38E-7 | 2.58E-5 |
| SNB-19 | 0.525 | 1.847 | 1.746 | 1.825 | 0.902 | 0.874 | 0.101 | 92 | 98 | 29 | 26 | -81 | 4.92E-7 | 1.76E-5 | 5.15E-5 |
| SNB-75 | 0.803 | 1.456 | 1.313 | 1.320 | 0.603 | 0.802 | 0.185 | 78 | 79 | -25 | . | -77 | 1.91E-7 | 5.76E-7 | 4.46E-5 |
| U251 | 0.462 | 2.042 | 1.883 | 1.922 | 0.995 | 0.681 | 0.076 | 90 | 92 | 34 | 14 | -84 | 5.28E-7 | 1.39E-5 | 4.52E-5 |
| **Melanoma** | | | | | | | | | | | | | | | |
| LOX IMVI | 0.674 | 3.256 | 3.201 | 3.187 | 1.460 | 0.350 | 0.108 | 98 | 97 | 30 | -48 | -84 | 5.10E-7 | 2.44E-6 | 1.13E-5 |
| MALME-3M | 0.756 | 1.750 | 1.675 | 1.570 | 1.159 | 1.019 | 0.100 | 92 | 82 | 41 | 26 | -87 | 5.90E-7 | 1.71E-5 | 4.73E-5 |
| M14 | 0.457 | 1.781 | 1.702 | 1.662 | 0.367 | 0.563 | 0.156 | 94 | 91 | -20 | 8 | -66 | 2.34E-7 | . | 6.08E-5 |
| MDA-MB-435 | 0.683 | 2.706 | 2.622 | 1.856 | 0.297 | 0.807 | 0.108 | 96 | 58 | -57 | 6 | -84 | 1.17E-7 | . | . |
| SK-MEL-2 | 1.570 | 3.200 | 3.146 | 3.183 | 2.058 | 1.849 | 0.462 | 97 | 99 | 30 | 17 | -71 | 5.12E-7 | 1.57E-5 | 5.82E-5 |
| SK-MEL-28 | 0.766 | 2.001 | 2.130 | 1.897 | 1.389 | 1.031 | 0.364 | 110 | 92 | 50 | 21 | -52 | 1.03E-6 | 1.95E-5 | 9.26E-5 |
| SK-MEL-5 | 1.077 | 3.189 | 3.072 | 3.097 | 1.679 | 0.979 | 0.012 | 94 | 96 | 29 | -9 | -99 | 4.79E-7 | 5.73E-6 | 2.85E-5 |
| UACC-257 | 0.875 | 2.073 | 1.991 | 1.953 | 1.522 | 1.172 | 0.726 | 93 | 90 | 54 | 25 | -17 | 1.37E-6 | 3.92E-5 | > 1.00E-4 |
| UACC-62 | 0.801 | 2.727 | 2.638 | 2.450 | 1.154 | 0.880 | 0.158 | 95 | 86 | 18 | 4 | -80 | 3.38E-7 | 1.12E-5 | 4.38E-5 |
| **Ovarian Cancer** | | | | | | | | | | | | | | | |
| IGROV1 | 0.465 | 1.916 | 1.920 | 1.912 | 0.875 | 0.471 | 0.203 | 100 | 100 | 28 | . | -56 | 4.96E-7 | 1.02E-5 | 7.70E-5 |
| OVCAR-3 | 0.620 | 1.858 | 1.824 | 1.839 | 0.463 | 0.449 | 0.050 | 97 | 98 | -25 | -28 | -92 | 2.46E-7 | 6.24E-7 | 2.23E-5 |
| OVCAR-4 | 0.627 | 1.440 | 1.391 | 1.410 | 1.070 | 0.781 | 0.353 | 94 | 96 | 54 | 19 | -44 | 1.33E-6 | 2.00E-5 | > 1.00E-4 |
| OVCAR-5 | 0.722 | 1.973 | 1.955 | 1.937 | 1.224 | 0.674 | 0.434 | 99 | 97 | 40 | -7 | -40 | 6.71E-7 | 7.19E-6 | > 1.00E-4 |
| OVCAR-8 | 0.584 | 2.522 | 2.446 | 2.452 | 1.273 | 0.821 | 0.757 | 96 | 96 | 36 | 12 | 9 | 5.78E-7 | > 1.00E-4 | > 1.00E-4 |
| NCI/ADR-RES | 0.835 | 2.589 | 2.466 | 2.399 | 0.814 | 0.837 | 0.412 | 93 | 89 | -3 | . | -51 | 2.67E-7 | . | 9.68E-5 |
| SK-OV-3 | 0.843 | 1.777 | 1.764 | 1.792 | 1.295 | 1.139 | 0.750 | 99 | 102 | 48 | 32 | -11 | 9.31E-7 | 5.51E-5 | > 1.00E-4 |
| **Renal Cancer** | | | | | | | | | | | | | | | |
| 786-0 | 0.916 | 2.673 | 2.565 | 2.522 | 1.231 | 1.076 | 0.069 | 94 | 91 | 18 | 9 | -92 | 3.66E-7 | 1.23E-5 | 3.82E-5 |
| A498 | 1.638 | 2.422 | 2.133 | 2.250 | 1.535 | 1.218 | 0.154 | 63 | 78 | -6 | -26 | -91 | 2.15E-7 | 8.42E-7 | 2.37E-5 |
| ACHN | 0.361 | 1.411 | 1.353 | 1.455 | 0.695 | 0.292 | 0.111 | 95 | 104 | 32 | -19 | -69 | 5.61E-7 | 4.20E-6 | 4.12E-5 |
| CAKI-1 | 0.606 | 2.119 | 1.935 | 1.571 | 1.073 | 0.841 | 0.212 | 88 | 64 | 31 | 16 | -65 | 2.63E-7 | 1.56E-5 | 6.51E-5 |
| RXF 393 | 0.993 | 1.970 | 1.858 | 1.881 | 0.739 | 0.908 | 0.075 | 89 | 91 | -26 | -9 | -92 | 2.24E-7 | 6.03E-7 | 3.12E-5 |
| SN12C | 0.637 | 2.348 | 2.320 | 2.341 | 1.164 | 0.719 | 0.472 | 98 | 100 | 31 | 5 | -26 | 5.26E-7 | 1.43E-5 | > 1.00E-4 |
| TK-10 | 0.756 | 1.606 | 1.448 | 1.550 | 1.163 | 0.738 | 0.306 | 81 | 93 | 48 | -2 | -60 | 8.99E-7 | 8.97E-6 | 6.80E-5 |
| UO-31 | 0.717 | 1.747 | 1.616 | 1.630 | 1.164 | 0.840 | 0.190 | 87 | 89 | 43 | 12 | -74 | 7.14E-7 | 1.38E-5 | 5.31E-5 |
| **Prostate Cancer** | | | | | | | | | | | | | | | |
| PC-3 | 0.553 | 1.908 | 1.819 | 1.848 | 0.897 | 0.780 | 0.197 | 93 | 96 | 25 | 17 | -64 | 4.46E-7 | 1.61E-5 | 6.65E-5 |
| DU-145 | 0.466 | 1.713 | 1.728 | 1.704 | 0.475 | 0.541 | 0.088 | 101 | 99 | 1 | 6 | -81 | 3.16E-7 | 1.17E-5 | 4.39E-5 |
| **Breast Cancer** | | | | | | | | | | | | | | | |
| MCF7 | 0.488 | 2.578 | 2.300 | 2.463 | 0.995 | 0.659 | 0.359 | 87 | 94 | 24 | 8 | -26 | 4.30E-7 | 1.72E-5 | > 1.00E-4 |
| MDA-MB-231/ATCC | 0.639 | 1.555 | 1.475 | 1.507 | 1.011 | 0.609 | 0.348 | 91 | 95 | 41 | -5 | -46 | 6.69E-7 | 7.87E-6 | > 1.00E-4 |
| HS 578T | 0.834 | 1.891 | 1.766 | 1.842 | 1.010 | 0.887 | 0.558 | 88 | 95 | 17 | 5 | -33 | 3.77E-7 | 1.35E-5 | > 1.00E-4 |
| BT-549 | 1.253 | 2.627 | 2.551 | 2.634 | 1.704 | 1.067 | 0.482 | 94 | 100 | 33 | -15 | -62 | 5.57E-7 | 4.88E-6 | 5.65E-5 |
| MDA-MB-468 | 0.922 | 2.087 | 1.966 | 2.049 | 1.481 | 0.995 | 0.245 | 90 | 97 | 48 | 6 | -73 | 9.08E-7 | 1.20E-5 | 5.08E-5 |

Figure S40. NCI60 screening on compound **12**.

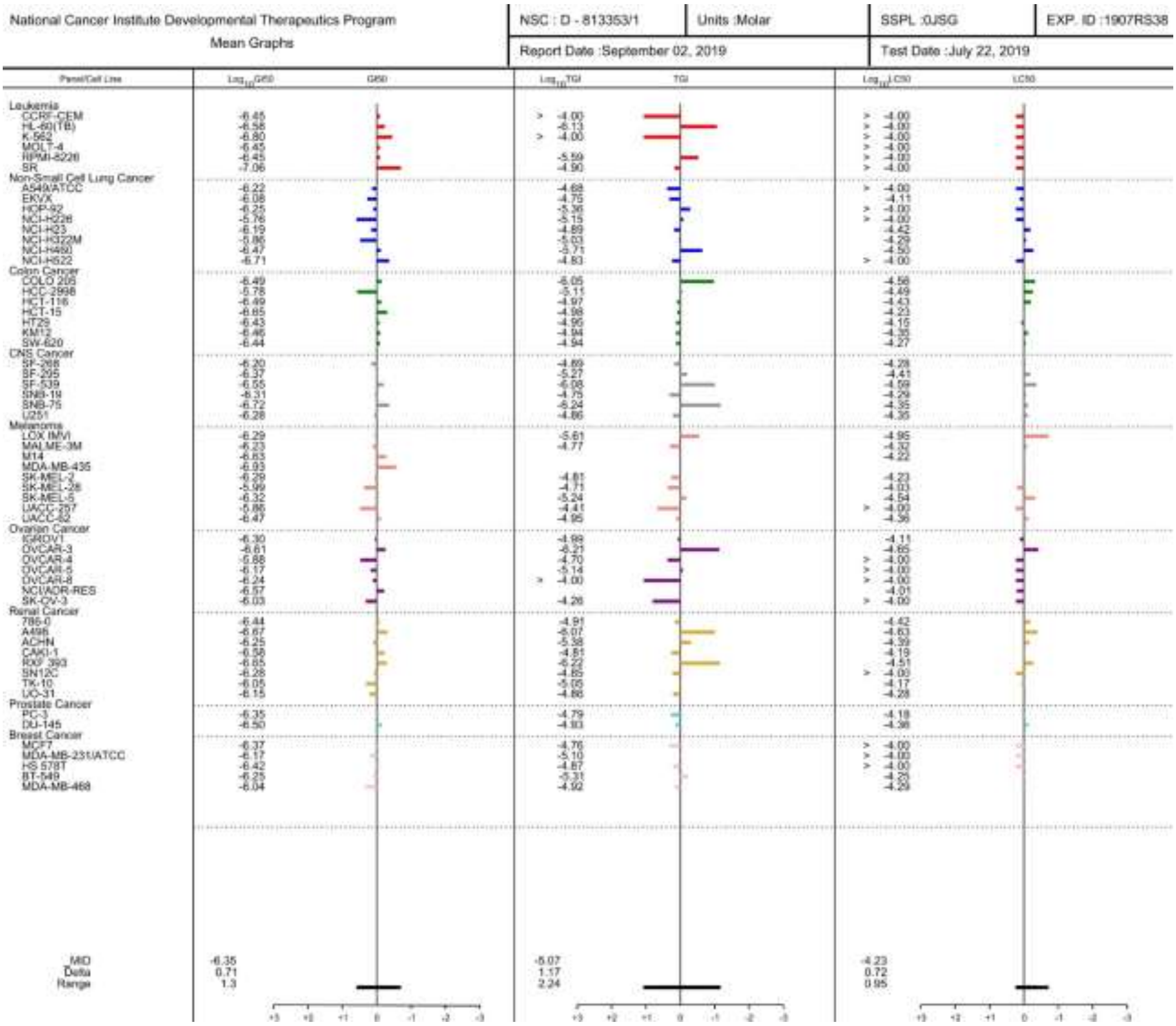


Figure S41. NCI60 screening on compound **12**.

# National Cancer Institute Developmental Therapeutics Program
## In-Vitro Testing Results

| | | | |
|---|---|---|---|
| NSC : D - 813354 / 1 | Experiment ID : 1905NS00 | Test Type : 08 | Units : Molar |
| Report Date : June 03, 2019 | Test Date : May 06, 2019 | QNS : | MC : |
| COMI : FR9 | Stain Reagent : SRB Dual-Pass Related | SSPL : 0JSG | |

| | | | Log10 Concentration | | | | | | | | | | | | | | |
|---|---|---|---|---|---|---|---|---|---|---|---|---|---|---|---|---|---|
| | Time | | Mean Optical Densities | | | | | Percent Growth | | | | | | | | | |
| Panel/Cell Line | Zero | Ctrl | -8.0 | -7.0 | -6.0 | -5.0 | -4.0 | -8.0 | -7.0 | -6.0 | -5.0 | -4.0 | GI50 | TGI | LC50 |
| Leukemia | | | | | | | | | | | | | | | |
| CCRF-CEM | 0.392 | 2.236 | 2.234 | 2.269 | 0.793 | 0.472 | 0.429 | 100 | 102 | 22 | 4 | 2 | 4.44E-7 | > 1.00E-4 | > 1.00E-4 |
| HL-60(TB) | 0.974 | 3.150 | 3.018 | 3.046 | 2.689 | 0.933 | 1.036 | 94 | 95 | 79 | -4 | 3 | 2.22E-6 | . | > 1.00E-4 |
| K-562 | 0.262 | 2.397 | 2.442 | 2.352 | 0.609 | 0.285 | 0.450 | 102 | 98 | 16 | 1 | 9 | 3.86E-7 | > 1.00E-4 | > 1.00E-4 |
| MOLT-4 | 0.579 | 2.988 | 2.800 | 2.770 | 2.377 | 0.568 | 0.631 | 92 | 91 | 75 | -2 | 2 | 2.10E-6 | . | > 1.00E-4 |
| RPMI-8226 | 0.482 | 1.524 | 1.475 | 1.479 | 0.710 | 0.420 | 0.319 | 95 | 96 | 22 | -13 | -34 | 4.16E-7 | 4.25E-6 | > 1.00E-4 |
| SR | 0.465 | 1.989 | 1.718 | 1.744 | 0.883 | 0.449 | 0.512 | 82 | 84 | 27 | -4 | 3 | 3.98E-7 | . | > 1.00E-4 |
| Non-Small Cell Lung Cancer | | | | | | | | | | | | | | | |
| A549/ATCC | 0.507 | 2.711 | 2.631 | 2.616 | 2.412 | 0.618 | 0.322 | 96 | 96 | 86 | 5 | -37 | 2.80E-6 | 1.32E-5 | > 1.00E-4 |
| EKVX | 0.867 | 2.588 | 2.479 | 2.521 | 2.394 | 0.033 | 0.036 | 94 | 96 | 89 | -96 | -96 | 1.62E-6 | 3.02E-6 | 5.62E-6 |
| HOP-62 | 0.661 | 2.507 | 2.377 | 2.417 | 2.212 | 0.619 | 0.507 | 93 | 95 | 84 | -6 | -23 | 2.38E-6 | 8.49E-6 | > 1.00E-4 |
| HOP-92 | 1.406 | 2.040 | 1.855 | 1.928 | 1.690 | 0.360 | 0.710 | 71 | 82 | 45 | -74 | -50 | 7.27E-7 | 2.38E-6 | . |
| NCI-H226 | 0.905 | 1.643 | 1.562 | 1.653 | 1.485 | 0.296 | 0.036 | 89 | 101 | 79 | -67 | -96 | 1.57E-6 | 3.45E-6 | 7.61E-6 |
| NCI-H23 | 0.679 | 2.400 | 2.295 | 2.351 | 1.946 | 0.153 | 0.056 | 94 | 97 | 74 | -77 | -92 | 1.43E-6 | 3.07E-6 | 6.58E-6 |
| NCI-H322M | 0.822 | 2.282 | 2.202 | 2.263 | 2.143 | 0.127 | 0.006 | 95 | 99 | 90 | -85 | -99 | 1.70E-6 | 3.29E-6 | 6.34E-6 |
| NCI-H460 | 0.316 | 2.688 | 2.770 | 2.800 | 2.560 | 0.136 | 0.017 | 103 | 105 | 95 | -57 | -95 | 1.97E-6 | 4.21E-6 | 9.00E-6 |
| NCI-H522 | 0.843 | 2.125 | 2.036 | 2.063 | 0.757 | 0.348 | 0.458 | 93 | 95 | -10 | -59 | -46 | 2.68E-7 | 7.99E-7 | . |
| Colon Cancer | | | | | | | | | | | | | | | |
| COLO 205 | 0.369 | 1.812 | 1.922 | 2.060 | 1.618 | 0.233 | 0.332 | 108 | 117 | 87 | -37 | -10 | 1.98E-6 | 5.02E-6 | > 1.00E-4 |
| HCC-2998 | 0.890 | 2.957 | 2.779 | 2.914 | 2.592 | 0.142 | 0.164 | 91 | 98 | 82 | -84 | -82 | 1.56E-6 | 3.13E-6 | 6.24E-6 |
| HCT-116 | 0.372 | 3.159 | 2.997 | 3.085 | 2.365 | 0.064 | 0.239 | 94 | 97 | 71 | -83 | -36 | 1.38E-6 | 2.91E-6 | . |
| HCT-15 | 0.304 | 1.922 | 1.836 | 1.830 | 0.774 | 0.074 | 0.078 | 95 | 94 | 29 | -76 | -75 | 4.78E-7 | 1.89E-6 | 5.69E-6 |
| HT29 | 0.303 | 1.889 | 1.864 | 2.085 | 0.776 | 0.142 | 0.186 | 98 | 112 | 30 | -53 | -39 | 5.69E-7 | 2.29E-6 | . |
| KM12 | 0.634 | 2.995 | 3.017 | 3.000 | 2.435 | 0.187 | 0.054 | 101 | 100 | 76 | -71 | -91 | 1.51E-6 | 3.31E-6 | 7.24E-6 |
| SW-620 | 0.311 | 1.717 | 1.742 | 1.727 | 0.674 | 0.112 | 0.018 | 102 | 101 | 26 | -64 | -94 | 4.75E-7 | 1.94E-6 | 6.96E-6 |
| CNS Cancer | | | | | | | | | | | | | | | |
| SF-268 | 0.594 | 1.990 | 1.915 | 1.982 | 1.832 | 0.156 | 0.242 | 95 | 99 | 89 | -74 | -59 | 1.73E-6 | 3.51E-6 | 7.14E-6 |
| SF-295 | 0.774 | 3.060 | 2.919 | 3.038 | 2.975 | 0.253 | 0.045 | 94 | 99 | 96 | -67 | -94 | 1.92E-6 | 3.88E-6 | 7.84E-6 |
| SF-539 | 0.811 | 2.465 | 2.456 | 2.607 | 2.465 | 0.116 | 0.103 | 99 | 109 | 100 | -86 | -87 | 1.86E-6 | 3.45E-6 | 6.42E-6 |
| SNB-19 | 0.543 | 2.151 | 2.062 | 2.163 | 1.896 | 0.131 | 0.103 | 94 | 101 | 84 | -76 | -81 | 1.63E-6 | 3.35E-6 | 6.88E-6 |
| SNB-75 | 0.908 | 1.553 | 1.430 | 1.486 | 1.385 | 0.111 | 0.081 | 81 | 90 | 74 | -88 | -91 | 1.41E-6 | 2.87E-6 | 5.84E-6 |
| U251 | 0.435 | 2.202 | 2.096 | 2.114 | 1.893 | 0.054 | 0.169 | 94 | 95 | 83 | -88 | -61 | 1.55E-6 | 3.06E-6 | 6.01E-6 |
| Melanoma | | | | | | | | | | | | | | | |
| LOX IMVI | 0.433 | 2.664 | 2.464 | 2.596 | 1.267 | 0.022 | 0.154 | 91 | 97 | 37 | -95 | -64 | 6.14E-7 | 1.92E-6 | 4.58E-6 |
| MALME-3M | 0.980 | 2.475 | 2.456 | 2.443 | 2.173 | 0.486 | 0.763 | 99 | 98 | 80 | -50 | -22 | 1.69E-6 | 4.10E-6 | . |
| M14 | 0.554 | 2.117 | 2.054 | 2.043 | 1.438 | 0.247 | 0.336 | 96 | 95 | 57 | -55 | -39 | 1.14E-6 | 3.20E-6 | . |
| MDA-MB-435 | 0.495 | 2.268 | 2.262 | 2.228 | 0.475 | 0.083 | 0.050 | 100 | 98 | -4 | -83 | -90 | 2.94E-7 | 9.13E-7 | 3.81E-6 |
| SK-MEL-2 | 1.310 | 2.575 | 2.540 | 2.602 | 2.385 | 0.857 | 0.295 | 97 | 102 | 85 | -35 | -77 | 1.96E-6 | 5.14E-6 | 2.29E-5 |
| SK-MEL-28 | 0.771 | 2.327 | 2.362 | 2.471 | 2.193 | 0.299 | 0.072 | 102 | 109 | 91 | -61 | -91 | 1.87E-6 | 3.97E-6 | 8.43E-6 |
| SK-MEL-5 | 0.861 | 3.014 | 2.917 | 3.025 | 2.488 | 0.033 | 0.004 | 96 | 101 | 76 | -96 | -100 | 1.41E-6 | 2.75E-6 | 5.38E-6 |
| UACC-257 | 1.063 | 2.601 | 2.539 | 2.575 | 2.278 | 0.760 | 0.838 | 96 | 98 | 79 | -29 | -21 | 1.86E-6 | 5.43E-6 | > 1.00E-4 |
| UACC-62 | 0.970 | 3.154 | 3.063 | 3.085 | 2.414 | 0.423 | 0.028 | 96 | 97 | 66 | -56 | -97 | 1.35E-6 | 3.46E-6 | 8.86E-6 |
| Ovarian Cancer | | | | | | | | | | | | | | | |
| IGROV1 | 0.414 | 1.968 | 1.907 | 2.007 | 1.420 | 0.166 | 0.211 | 96 | 102 | 65 | -60 | -49 | 1.31E-6 | 3.30E-6 | . |
| OVCAR-3 | 0.638 | 2.126 | 2.153 | 2.114 | 1.331 | 0.043 | 0.029 | 102 | 99 | 47 | -93 | -96 | 8.61E-7 | 2.15E-6 | 4.90E-6 |
| OVCAR-4 | 0.611 | 1.531 | 1.530 | 1.551 | 1.382 | 0.181 | 0.054 | 100 | 102 | 84 | -70 | -91 | 1.65E-6 | 3.49E-6 | 7.37E-6 |
| OVCAR-5 | 0.651 | 1.603 | 1.532 | 1.641 | 1.531 | 0.142 | 0.049 | 93 | 104 | 92 | -78 | -93 | 1.77E-6 | 3.48E-6 | 6.84E-6 |
| OVCAR-8 | 0.632 | 2.848 | 2.730 | 2.793 | 2.476 | 0.696 | 0.515 | 95 | 97 | 83 | 3 | -19 | 2.59E-6 | 1.36E-5 | > 1.00E-4 |
| NCI/ADR-RES | 0.679 | 2.294 | 2.148 | 2.284 | 0.956 | 0.453 | 0.425 | 91 | 99 | 17 | -33 | -37 | 3.98E-7 | 2.19E-6 | > 1.00E-4 |
| SK-OV-3 | 0.685 | 1.974 | 1.913 | 1.915 | 1.899 | 1.067 | 0.206 | 95 | 95 | 94 | 30 | -70 | 4.83E-6 | 1.98E-5 | 6.31E-5 |
| Renal Cancer | | | | | | | | | | | | | | | |
| 786-0 | 0.683 | 2.716 | 2.586 | 2.712 | 2.284 | 0.070 | 0.290 | 94 | 100 | 79 | -90 | -58 | 1.48E-6 | 2.93E-6 | 5.80E-6 |
| A498 | 2.179 | 2.859 | 2.860 | 2.808 | 2.852 | 0.897 | 0.044 | 100 | 92 | 99 | -59 | -98 | 2.04E-6 | 4.23E-6 | 8.79E-6 |
| ACHN | 0.404 | 1.795 | 1.774 | 1.805 | 1.364 | 0.168 | 0.066 | 98 | 101 | 69 | -58 | -84 | 1.41E-6 | 3.48E-6 | 8.59E-6 |
| CAKI-1 | 1.158 | 3.179 | 3.078 | 3.083 | 2.987 | 0.705 | 0.011 | 95 | 95 | 90 | -39 | -99 | 2.05E-6 | 4.99E-6 | 1.52E-5 |
| RXF 393 | 0.939 | 1.581 | 1.549 | 1.566 | 1.288 | 0.237 | 0.430 | 95 | 98 | 54 | -75 | -54 | 1.08E-6 | 2.64E-6 | 6.43E-6 |
| SN12C | 0.616 | 2.574 | 2.451 | 2.592 | 2.203 | 0.112 | 0.063 | 94 | 101 | 81 | -82 | -90 | 1.55E-6 | 3.14E-6 | 6.38E-6 |
| TK-10 | 0.880 | 2.146 | 1.927 | 2.035 | 2.175 | 0.145 | 0.423 | 83 | 91 | 102 | -84 | -52 | 1.91E-6 | 3.55E-6 | 6.60E-6 |
| UO-31 | 0.563 | 1.765 | 1.677 | 1.751 | 1.482 | 0.002 | 0.097 | 93 | 99 | 76 | -100 | -83 | 1.41E-6 | 2.72E-6 | 5.22E-6 |
| Prostate Cancer | | | | | | | | | | | | | | | |
| PC-3 | 0.477 | 2.121 | 2.058 | 2.100 | 1.278 | 0.246 | 0.171 | 96 | 99 | 49 | -49 | -64 | 9.43E-7 | 3.17E-6 | 1.24E-5 |
| DU-145 | 0.452 | 1.673 | 1.702 | 1.783 | 1.458 | 0.006 | 0.013 | 102 | 109 | 82 | -99 | -97 | 1.51E-6 | 2.85E-6 | 5.38E-6 |
| Breast Cancer | | | | | | | | | | | | | | | |
| MCF7 | 0.544 | 2.559 | 2.270 | 2.348 | 1.120 | 0.175 | 0.242 | 86 | 90 | 29 | -68 | -56 | 4.45E-7 | 1.98E-6 | 6.53E-6 |
| MDA-MB-231/ATCC | 0.715 | 1.811 | 1.799 | 1.754 | 1.620 | 0.211 | 0.111 | 99 | 95 | 83 | -71 | -85 | 1.63E-6 | 3.46E-6 | 7.34E-6 |
| HS 578T | 1.078 | 2.002 | 1.900 | 1.956 | 1.898 | 0.984 | 0.805 | 89 | 95 | 89 | -9 | -25 | 2.50E-6 | 8.14E-6 | > 1.00E-4 |
| BT-549 | 1.125 | 2.336 | 2.215 | 2.236 | 2.025 | 0.260 | 0.486 | 90 | 92 | 74 | -77 | -57 | 1.45E-6 | 3.10E-6 | 6.64E-6 |
| T-47D | 0.600 | 1.883 | 1.720 | 1.799 | 1.275 | 0.429 | 0.479 | 87 | 93 | 53 | -29 | -20 | 1.08E-6 | 4.44E-6 | > 1.00E-4 |
| MDA-MB-468 | 0.586 | 1.363 | 1.347 | 1.346 | 0.625 | 0.136 | 0.137 | 98 | 98 | 5 | -77 | -77 | 3.28E-7 | 1.15E-6 | 4.70E-6 |

Figure S42. NCI60 screening on compound **13**.

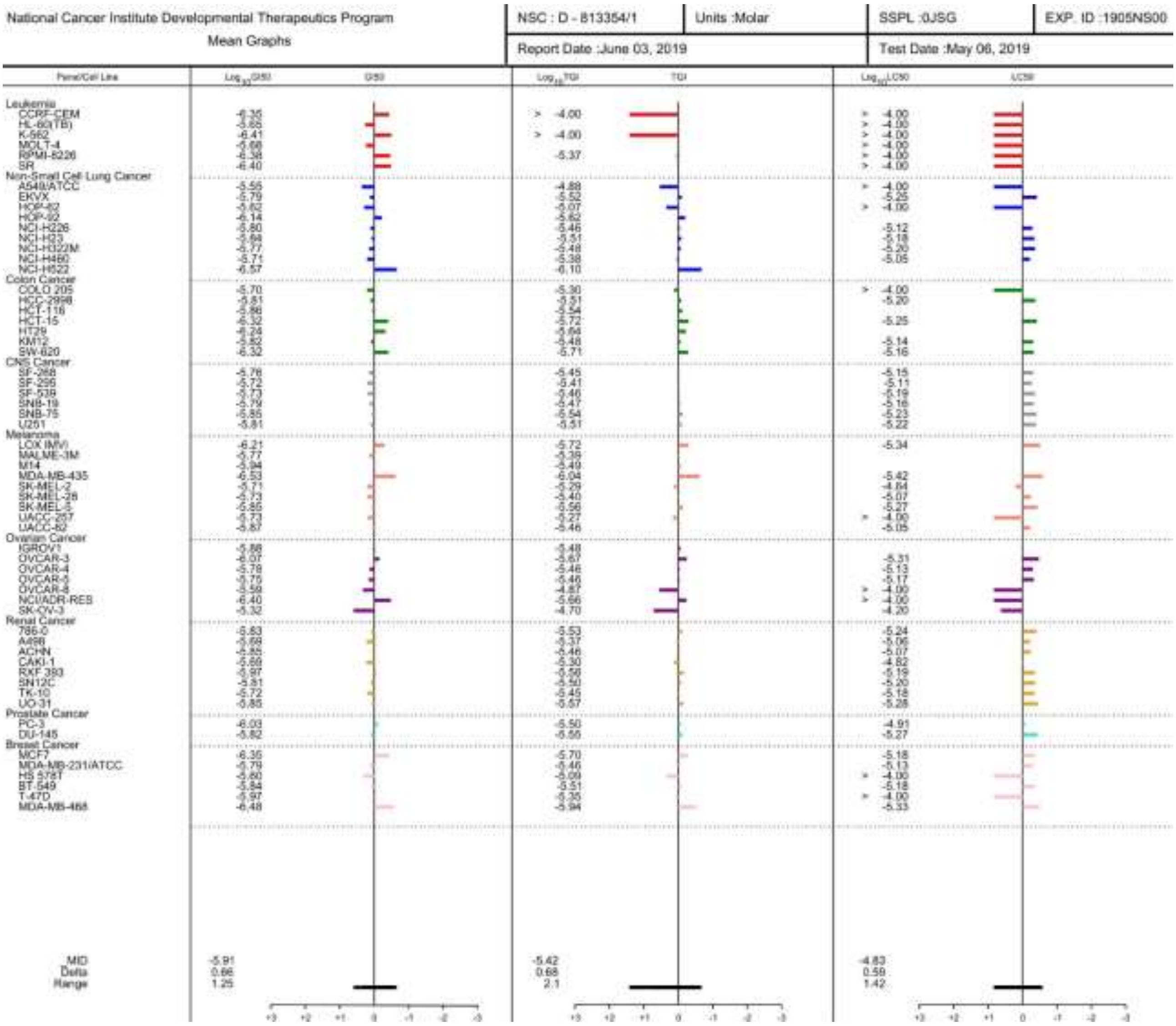


Figure S43. NCI60 screening on compound **13**.

| Developmental Therapeutics Program<br>One Dose Mean Graph | NSC: D-822613 / 1 | Conc: 1.00E-5 Molar | Test Date: Jan 27, 2020 |
|---|---|---|---|
| | Experiment ID: 2001OS11 | | Report Date: Feb 19, 2020 |

| Panel/Cell Line | Growth Percent |
|---|---|
| Leukemia | |
| CCRF-CEM | 17.60 |
| HL-60(TB) | 1.65 |
| K-562 | 11.88 |
| MOLT-4 | 26.24 |
| RPMI-8226 | 6.88 |
| Non-Small Cell Lung Cancer | |
| A549/ATCC | 86.62 |
| EKVX | 24.88 |
| HOP-62 | 53.23 |
| HOP-92 | -24.96 |
| NCI-H226 | 62.64 |
| NCI-H23 | 27.74 |
| NCI-H322M | 89.92 |
| NCI-H460 | 35.53 |
| NCI-H522 | 18.25 |
| Colon Cancer | |
| COLO 205 | 34.27 |
| HCC-2998 | 42.68 |
| HCT-116 | 10.44 |
| HCT-15 | 10.25 |
| HT29 | 27.49 |
| KM12 | 62.13 |
| SW-620 | 21.53 |
| CNS Cancer | |
| SF-268 | 67.45 |
| SF-295 | 93.43 |
| SF-539 | 36.56 |
| SNB-19 | 80.70 |
| SNB-75 | -5.04 |
| U251 | 78.87 |
| Melanoma | |
| LOX IMVI | 14.88 |
| MALME-3M | 22.62 |
| M14 | 35.09 |
| MDA-MB-435 | 28.97 |
| SK-MEL-2 | 52.82 |
| SK-MEL-28 | 37.08 |
| SK-MEL-5 | 58.68 |
| UACC-257 | 52.09 |
| UACC-62 | 36.48 |
| Ovarian Cancer | |
| IGROV1 | 26.49 |
| OVCAR-3 | 35.34 |
| OVCAR-4 | 38.71 |
| OVCAR-5 | 31.79 |
| OVCAR-8 | 41.70 |
| NCI/ADR-RES | 23.80 |
| SK-OV-3 | 90.15 |
| Renal Cancer | |
| 786-0 | 56.36 |
| A498 | 67.67 |
| ACHN | 16.90 |
| CAKI-1 | 41.51 |
| RXF 393 | -2.13 |
| SN12C | 17.67 |
| TK-10 | 34.36 |
| UO-31 | 41.37 |
| Prostate Cancer | |
| PC-3 | 48.71 |
| DU-145 | 2.26 |
| Breast Cancer | |
| MCF7 | 19.32 |
| MDA-MB-231/ATCC | 31.69 |
| HS 578T | 47.11 |
| BT-549 | 16.99 |
| T-47D | 23.49 |
| MDA-MB-468 | 31.91 |
| Mean | 35.94 |
| Delta | 60.90 |
| Range | 118.39 |

Mean Growth Percent - Growth Percent

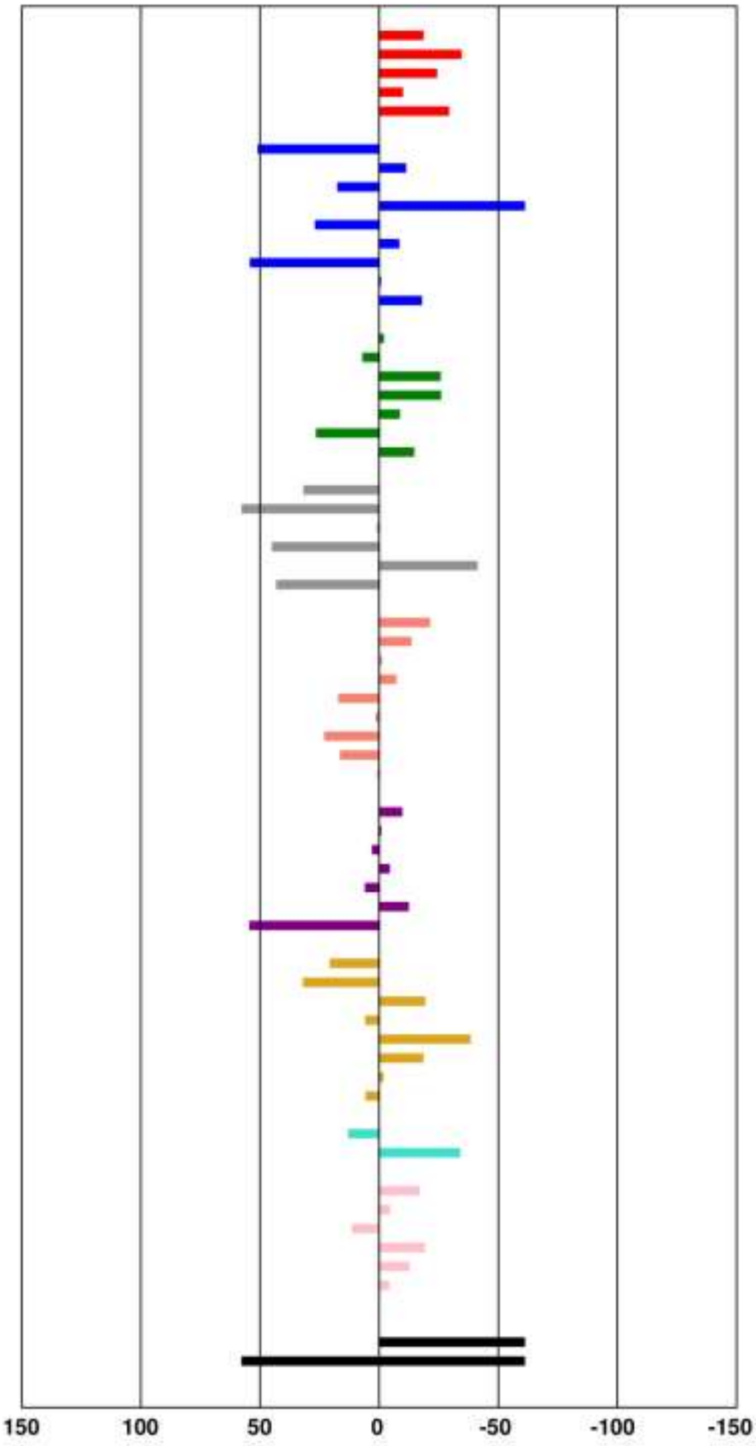


Figure S44. NCI60 screening on compound **13**.
